# Chromatin-Associated Proteins Revealed by SILAC-Proteomic Analysis Exhibit a High Likelihood of Requirement for Growth Fitness under DNA Damage Stress

**DOI:** 10.1155/2012/630409

**Published:** 2012-08-01

**Authors:** Han Wang, Pornpimol Tipthara, Lei Zhu, Suk Yean Poon, Kai Tang, Jianhua Liu

**Affiliations:** ^1^School of Biological Science, Nanyang Technological University, Singapore 637551; ^2^Systems Biology, Genome Institute of Singapore, Singapore 138672; ^3^Department of Biochemistry, Yong Loo Lin School of Medicine, National University of Singapore, Singapore 119077

## Abstract

Chromatin-associated nonhistone proteins (CHRAPs) are readily collected from the DNaseI digested crude chromatin preparation. In this study, we show that the absolute abundance-based label-free quantitative proteomic analysis fail to identify potential CHRAPs from the CHRAP-prep. This is because that the most-highly abundant cytoplasmic proteins such as ribosomal proteins are not effectively depleted in the CHRAP-prep. Ribosomal proteins remain the top-ranked abundant proteins in the CHRAP-prep. On the other hand, we show that relative abundance-based SILAC-mediated quantitative proteomic analysis is capable of discovering the potential CHRAPs in the CHRAP-prep when compared to the whole-cell-extract. Ribosomal proteins are depleted from the top SILAC ratio-ranked proteins. In contrast, nucleus-localized proteins or potential CHRAPs are enriched in the top SILAC-ranked proteins. Consistent with this, gene-ontology analysis indicates that CHRAP-associated functions such as transcription, regulation of chromatin structures, and DNA replication and repair are significantly overrepresented in the top SILAC-ranked proteins. Some of the novel CHRAPs are confirmed using the traditional method. Notably, phenotypic assessment reveals that the top SILAC-ranked proteins exhibit the high likelihood of requirement for growth fitness under DNA damage stress. Taken together, our results indicate that the SILAC-mediated proteomic approach is capable of determining CHRAPs without prior knowledge.

## 1. Background

Chromatin is a complex of DNA and proteins, in which the histones H2A, H2B, H3, and H4 are the major protein constituents [1, 2]. Chromatin remodeling through posttranslational modification of histones plays an important role in modulation of DNA-protein interaction and thus regulates various biological processes such as replication, DNA damage repair, and transcription [3]. Hence, identification of the chromatin associated nonhistone proteins (CHRAPs) would permit understanding the molecular mechanisms for chromatin remodeling and regulation of various biological processes.

Fission yeast is a useful model for analysis of RNA interference (RNAi) directed heterochromatin formation [4, 5]. Many CHRAPs have been identified by using the high-throughput proteomic analysis of protein complexes purified through the chromatin immunoprecipitation (ChIP) coupled with the tandem affinity protein purification (TAP) tagging method in which the known CHRAP is used as bait [6–12]. However, it is limited to the identification of the CHRAPs that are associated with the complexes containing the previously known CHRAPs.

A traditional assay for testing whether a protein of interest is associated with the chromatin includes the preparation of CHRAPs extracts (or CHRAP-prep) through collection of the released proteins from the DNaseI digested crude chromatin and western blot analysis [13–16]. By using this method, components of the origin recognition complex such as Orc1, Orc2, and Orc5 are found to be associated with the chromatin throughout the cell cycle [13, 14]. On the other hand, the ATR-like kinase Rad3 and the mitotic activator phosphatase Cdc25 are found to be temporally associated with chromatins upon DNA damage [15, 16]. The relative level of a CHRAP of interest in CHRAP-prep is clearly higher than that of whole cell extract (WCE) [13–16]. Nevertheless, it is unclear if highly abundant cytoplasm-localized proteins are effectively depleted from the CHRAP-prep. Effectively, depletion of the highly abundant non-CHRAPs such as ribosomal proteins is essential for *de novo* identification of CHRAPs through proteomic analysis of CHRAP-prep based on their absolute abundances.

We found that the top ranked proteins by levels of abundance in CHRAP-prep were predominated by the ribosomal proteins, suggesting that the highly abundant non-CHRAPs are not effectively removed in CHRAP-prep. Hence, simply based on the level of protein abundance in CHRAP-prep by using the high-throughput proteomic analysis is unlikely to reveal CHRAP candidates without prior knowledge. SILAC (stable isotope labeling with amino acids in cell culture)-mediated proteomic analysis has shown to permit the quantitative analysis of the relative protein levels between those labeled with and without heavy isotopes [17], allowing estimation of ratios between individual protein levels in CHRAP-prep versus WCE. By applying the SILAC-mediated high-throughput proteomic analysis, we show, in this study, that the highly abundant non-CHRAP ribosomal proteins are significantly depleted in the top ranked proteins by SILAC-ratio between CHRAP-prep and WCE. The top ranked proteins by SILAC-ratio are enriched for potential CHRAP candidates such as nucleus-localized proteins and CHRAP-associated functions such as chromatin structure organization, DNA replication and repair, transcription according to gene ontology analysis. Phenotypic assessment shows that the SILAC-enriched but not depleted nucleus-localized proteins exhibit the high likelihood of requirement for growth fitness in MMS (methyl methanesulfonate)-induced DNA damage stress. Taken together, our results indicate that SILAC-mediated proteomic analysis of CHRAP-prep is capable of identifying CHRAP candidates without prior knowledge. We propose that our approach can be complementary to the ChIP method coupled with TAP-tagging for identification of CHRAP-interacting partners.

## 2. Results and Discussion

**Figure 1:**
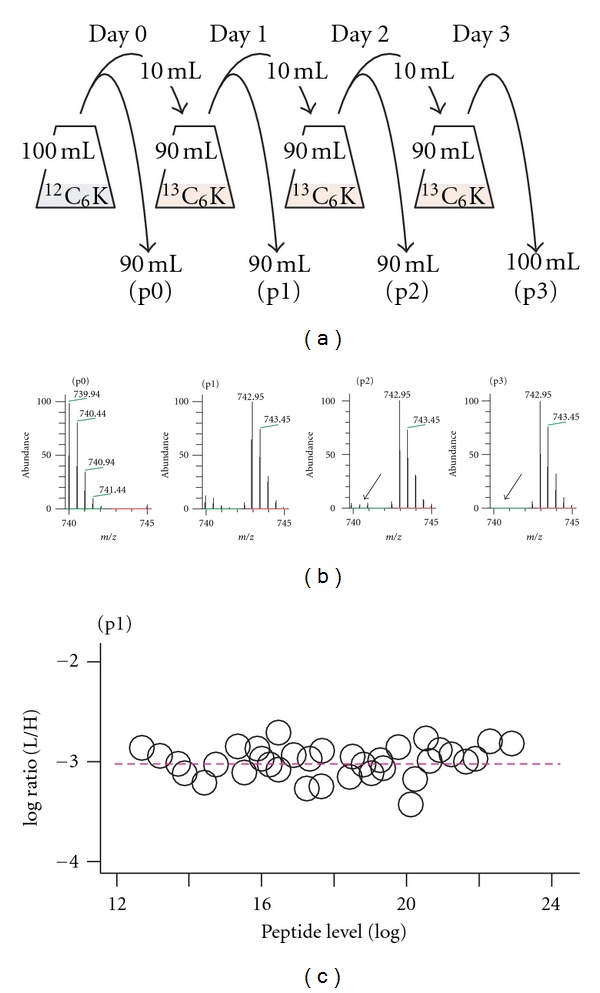
Heavy lysine is efficiently incorporated in fission yeast. (a) A schematic diagram shows the consecutive subculturing of cells in heavy lysine containing medium. (b) An MS spectrum of the Eno1 peptide AVGNVNNIIAPVVK. The spectrum of the peptide resulted from passages p0, p1, p2, and p3 is shown. Green and red lines indicate the *m/z* of light and heavy peptides, respectively. Arrow indicates the position of light peptide undetected in p2 and p3 cells. (c) The scatter plot shows the ratio of light and heavy peptides in the first passage (sample p1). *X*- and *Y*-axis indicate the abundance of individual peptides and the ratio of light and heavy peptide, respectively. Each dot represents a peptide.

In the SILAC proteomic analysis, SILAC-labeled samples are required to be fully incorporated with the heavy stable isotope-coupled lysine (e.g., ^13^C_6_-lysine or heavy-lysine) or arginine (e.g., ^13^C_6_-arginine or heavy-arginine) or both. To avoid the arginine-conversion problem [18], we applied heavy lysine alone in this study. The rate of incorporation with heavy-lysine in cells after various numbers of passages or subcultures in minimal medium supplemented with heavy-lysine was tested. In this test, each subculture was maintained for a day (i.e., equivalent to ~3 generations) before subsequent subculturing (Figure 1(a)).

The relative level of light versus heavy peptides/proteins was exemplified by the Eno1 peptide AVGNVNNIIAPVVK in various subcultures. As expected, no Eno1 peptide was detected to contain heavy-lysine in the initial culture (p0) prior to subculturing with the heavy lysine-containing medium (Figure 1(b), see p0). On the other hand, ~90% of the peptides were detected to contain heavy-lysine in the first subculture (p1) with the heavy lysine-containing medium (Figure 1(b), see p1). Hardly any light lysine-containing peptides were detected in the second (p2) or the third (p3) subcultures (Figure 1(b), see p2 and p3). This result indicates that heavy lysine is effectively incorporated into cellular protein in fission yeast.

To further test whether the heavy lysine was uniformly incorporated in all peptides/proteins besides Eno1, a slice of SDS-PAGE gel containing proteins derived from the first subculture was subjected to LC-MS/MS analysis. The ratio between light and heavy peptide levels in all of the ~100 peptides detected was found to be close to −3 in log 2 scale, indicating that the heavy lysine is uniformly incorporated in all proteins in the culture (Figure 1(c)). To ascertain proteins were fully incorporated with heavy lysine, cells derived from the third subculture (i.e., ~9 generations in the heavy-lysine-containing medium) were applied for SILAC analysis in this study.

**Figure 2:**
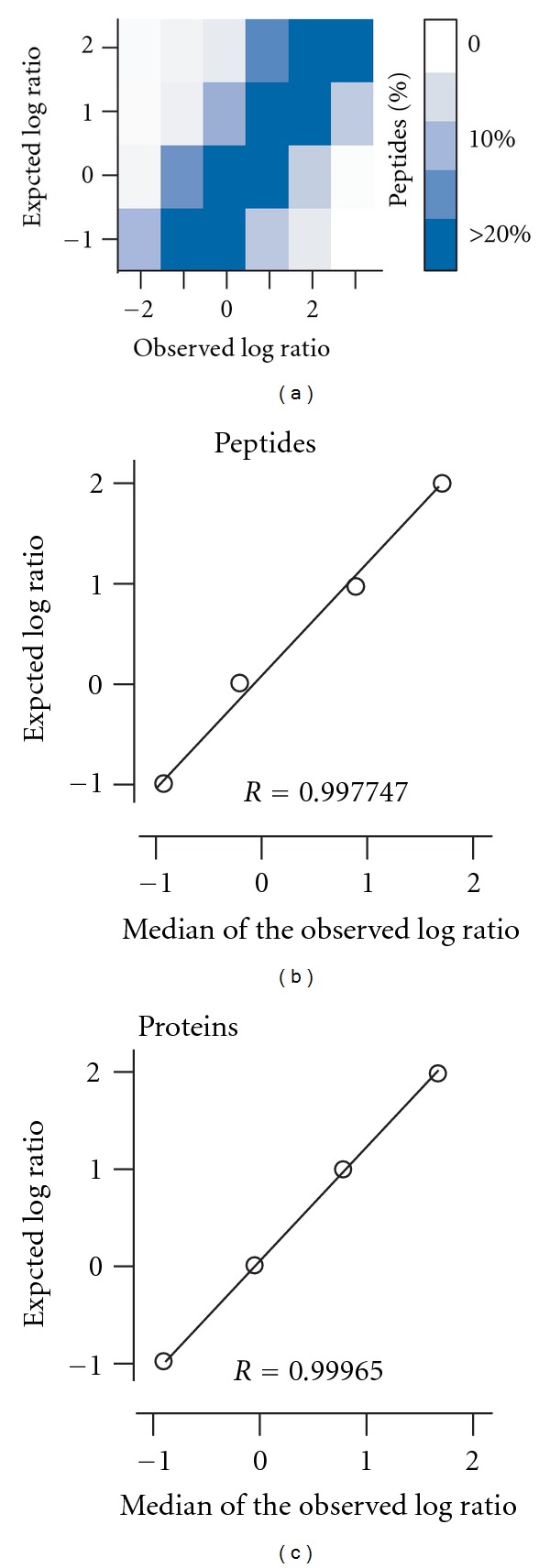
High sensitivity of peptide/protein ratio detection by SILAC analysis. (a) Distribution of detected ratios by SILAC is correlated with the expected ratio in the given samples. *X*- and *Y*-axis indicate the detected peptide ratios by SILAC and the expected ratio of the given samples, respectively. Color key is shown in (a). (b) The median of detected peptide ratios by SILAC is highly correlated with the expected ratio in the given samples. The correlation coefficient (*R*) is shown. (c) The median of protein ratios is highly correlated with the expected ratio.

Next, we wanted to assess the sensitivity by varying the ratios of light and heavy peptides/proteins in the given premixed samples. A slice of SDS-PAGE gel (i.e., containing >100 peptides) from each given sample was subjected to the proteomic analysis after in-gel trypsinization. To this end, the distribution of ratios of all peptides detected in SILAC analysis correlated well with the expected ratio in the given samples, suggesting that our SILAC protocol is adequate for the quantitative proteomic analysis (Figure 2(a)). This is consistent with the notion that SILAC methodology is excellent for quantitative proteomic analysis [17]. The correlation was apparent when the medians of ratios of all peptides detected in SILAC analysis were compared to the expected ratios of the given samples (Figure 2(b)). To estimate the ratios between light and heavy proteins, the median of the unique peptides levels was applied (see Section 4). Based on this estimation, protein ratios detected in SILAC analysis correlated well with the expected ratios of the given samples (Figure 2(c)). Hence, the median of unique peptide levels was used to estimate the level of proteins in this study.

**Figure 3:**
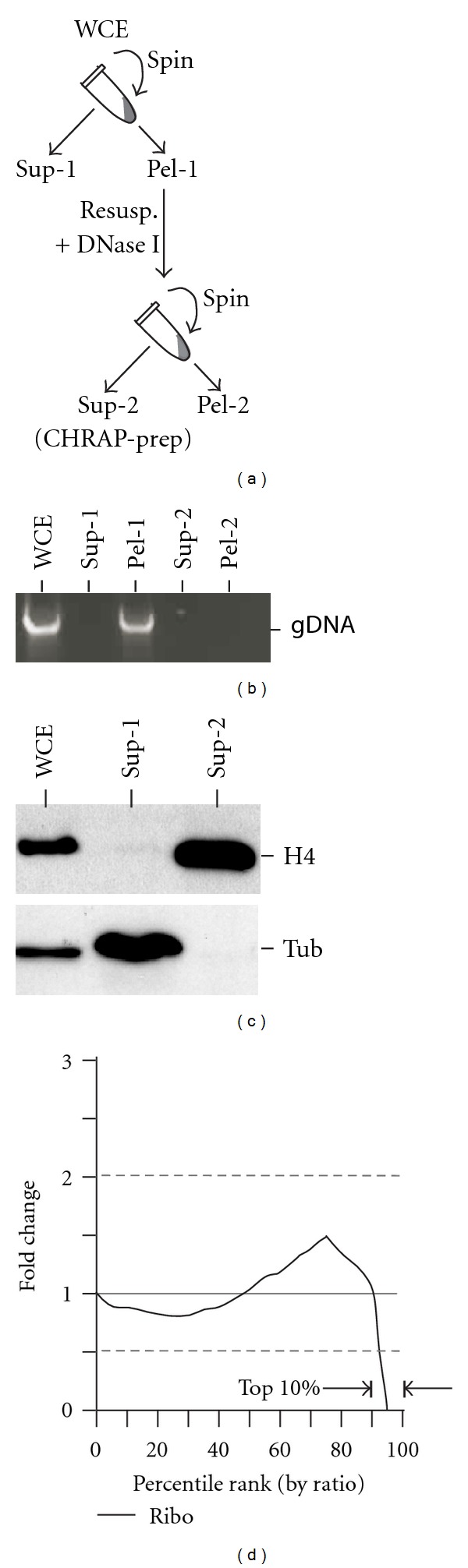
Abundant ribosomal proteins are effectively depleted in CHRAP-prep by SILAC. (a) A schematic diagram shows the steps of CHRAP preparation. WCE stands for whole cell extract; sup for supernatant; pel for pellet; and resusp. for resuspension. (b) The image of the agarose gel shows the presence or absence of genomic DNA (gDNA) in various samples indicated as in (a). (c) The image of western blot shows the presence or absence of histone H4 and soluble protein tubulin (tub). (d) Top 10% ranked proteins by SILAC ratios are depleted of ribosomal proteins. *X*-axis indicates the percentile ranks by SILAC ratios and *Y*-axis indicates the level of the ribosomal proteins.

It has been shown that a given proteins can be tested for its association with the chromatins by enriching the CHRAPs [13, 14]. Based on the protocols, we obtained CHRAP-prep (Figure 3(a)). It was clear that soluble proteins such as tubulin were depleted only in the soluble fraction (sup1) and chromatin-associated proteins such as histone H4 were enriched in the CHRAP-prep (or sup2) (Figures 3(b) and 3(c)). To test if the most abundant proteins in CHRAP-prep were CHRAPs, CHRAP-prep was subjected to the non-SILAC protein analysis. We found that the top 10% ranked proteins by absolute abundance in CHRAP-prep were overrepresented by the non-CHRAP ribosomal proteins when compared to the background level (i.e., 51.4% versus 14.9%; *P* value = 2.21*e* − 07) (see Supplementary Table S3 in Supplementary Material available online at doi:10.1155/2012/630409 and also see Section 4). This result indicates that the highly abundant non-CHRAPs ribosomal proteins are not effectively removed in CHRAP-prep.

We judged that the absolute abundance of the majority enriched CHRAPs might not be higher than that of the depleted ribosomal proteins due to their high abundance prior to the enrichment. On the other hand, CHRAPs would be top ranked by comparing levels of enrichment or ratios between CHRAP-prep (after enrichment) and WCE (before enrichment). To test this possibility, equal amounts of CHRAP-prep and heavy lysine-labeled WCE proteins were mixed and subjected to LC-MS/MS analysis using LTQ Orbitrap mass spectrometry (see Section 4).

A total of 507 proteins were identified based on the presence of paired light (i.e., CHRAP-prep) and heavy (i.e., WCE) peptides after selection of high quality peptide through the Trans-Proteomic Pipeline (http://tools.proteomecenter.org/) (Supplementary Table S2; see Section 4). By ranking the proteins using SILAC-ratios, we found that the occurrence of ribosomal proteins in the top 10% (or top 50) ranked proteins was ~35% lower than the background level (6% versus 9.5%). We found that the ribosomal protein occurrence in the top 10% ranked proteins by SILAC ratios was significantly lower than that in the top 10% ranked proteins by absolute abundances (6% versus 51.4%; *P*-value = 9.32*e* − 12). This result indicates that the abundant ribosomal proteins can be effectively reduced from the top 10% ranked proteins in SILAC analysis (Figure 3(d)).

We noted that the occurrence of ribosomal proteins in the second top 10% ranked proteins by SILAC ratios was not reduced when compared to the background level (i.e., 14% versus 9.5%). This result suggests that the enrichment for CHRAPs in the second top 10% ranked proteins was less effective. Hence, only the first top 10% ranked proteins by SILAC ratio were considered for enriching CHRAP candidates and were further analyzed in this study.

**Figure 4:**
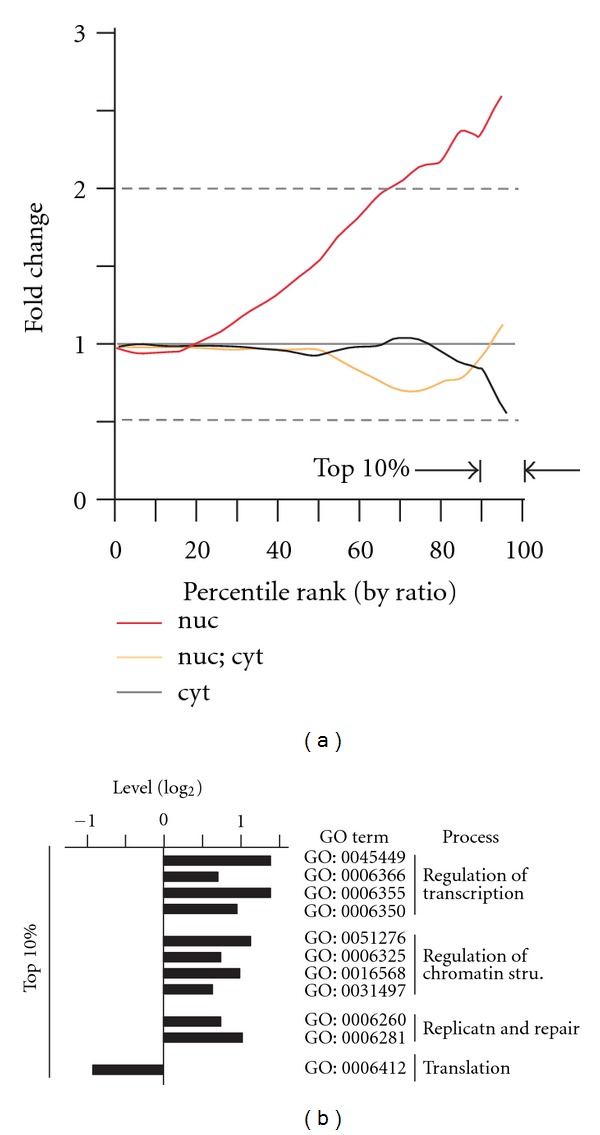
Top ranked proteins by SILAC ratios are enriched for nucleus-localized proteins and CHRAP-associated functions. (a) Nucleus-localized proteins are enriched in the top 10% ranked proteins by SILAC. Display is identical to Figure 3(d). Levels of nucleus-localized (nuc), dual-localized (nuc; cyt), and cytoplasm-localized (cyt) proteins are shown. (b) Top 10% ranked proteins by SILAC are enriched for CHRAP-associated functions based on gene ontology analysis.

CHRAPs would have nucleus-localization due to their association with chromatin. We, therefore, wanted to examine if the nucleus-localized proteins were particularly enriched in the top ranked proteins by SILAC ratios. Based on the subcellular localization characterized by Matsuyama et al. [19] and gene ontology (http://www.geneontology.org/), we found that the nucleus-localized proteins were significantly enriched in the top 10% ranked proteins by SILAC ratios when compared to the background level (i.e., 24% versus 9.47%; *P*-value = 2.0*e* − 03) (Figure 4(a)). This result supports the notion that the top 10% ranked proteins by SILAC ratios are enriched for CHARP candidates, suitable for CHRAP discovery without prior knowledge.

Next, we wanted to know if the CHRAP-associated functions such as chromatin modification and DNA replication and repair would be enriched in the top ranked proteins by SILAC ratios. Based on the gene ontology (i.e., biological process terms), gene functions such as transcription, chromatin modification, DNA replication and repair were over-represented in the top 10% ranked proteins (*P* value < 0.05) (Figure 4(b)). These results are consistent with a notion that the top ranked proteins by SILAC were enriched for CHRAP functions.

In the top 10% ranked proteins by SILAC ratios, we found Psm3, Cbh2, and C27f1.06c that are involved in chromosome organization and chromatin remodeling; Ddb1, Msh3, Spp1, and Uve1 that are involved in DNA replication and repair, and Eri1, C947.08c, Rpb1, and C530.05 that are involved in transcription (see Supplementary Table S2). These proteins would represent a small subset of CHRAPs that are relatively soluble, abundant, and constitutively associated with the chromatin. Deep analysis of CHRAP-prep using SILAC proteomics should allow identifying more proteins with CHRAP-associated functions without prior knowledge.

We also found Prp16, Smb1, and Mug161 that are involved in proteolysis and Ubr1, P8b7.11, and Rpt2 that are involved in mRNA splicing in the top ranked proteins by SILAC ratios. In fact, it is not unusual to find components of the proteolysis machinery that are associated with the chromatin. For instance, the ubiquitin E3 ligase Pcu4 is found to be associated with the RNA-induced transcriptional silencing (RITS) complex involved in heterochromatin assembly [20]; and the E3 ligase Ubr1 is associated with the Set1 complex involved in histone H3 methylation [21, 22]. On the other hand, proteins involved in mRNA splicing have been found to be assembled into the cotranscriptional spliceosome on chromosome [23].

**Figure 5:**
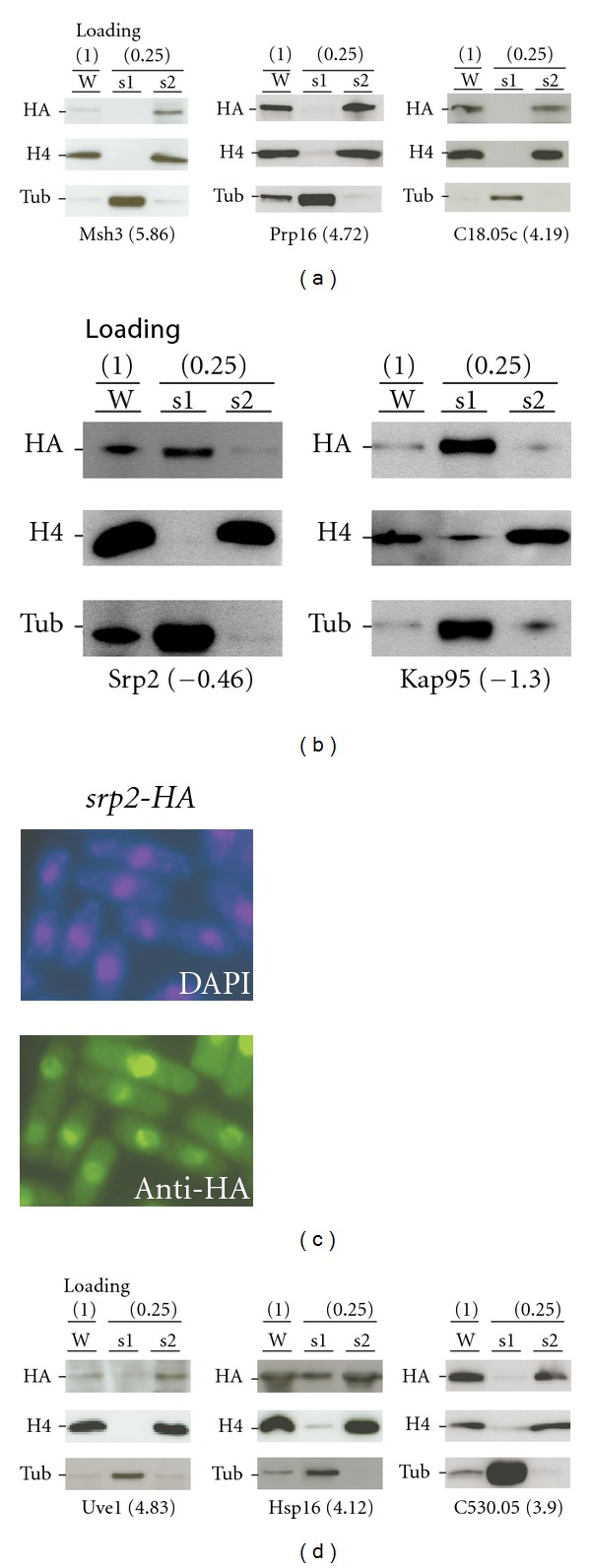
Traditional CHRAP assay to validate SILAC-enriched/depleted proteins. (a) SILAC-enriched nucleus localized proteins are association with the chromatin. Loading quantity of various protein samples such as whole cell extract (W), supernatant-1 (s1) and supernatant-2 (s2; the CHRAP-prep) is indicated in parentheses on the top. Tested proteins and their SILAC ratio in parentheses are indicated at the bottom. (b) SILAC-depleted proteins are unassociated with the chromatin. The display is identical to (a). (c) Subcellular localization of the HA-tagged Srp2 proteins. (d) SILAC-enriched dual-localized proteins are associated with the chromatin. The display is identical to (a).

We noted that some nucleus-localized proteins were not listed in the top 10% ranked proteins by SILAC ratios. Those nucleus-localized proteins might not be associated with the chromatins. To test this possibility, randomly selected 3 top SILAC-ranked proteins Msh3, Prp16, and C18.05c (e.g., their SILAC ratios were 5.86, 4.72, and 4.19 in log 2 scale, resp.) and 2 other proteins Srp2 and Kap95 (e.g., their SILAC ratios are −0.46 and −1.30 in log 2 scale) (see Supplementary Table S2) were subjected to the traditional assay for CHRAPs (see Section 4). The analysis indicated that the SILAC most enriched nucleus-localized proteins were the true CHRAPs (Figure 5(a)). On the other hand, the SILAC depleted nucleus-localized proteins were not CHRAPs (Figure 5(b)). To ascertain that the subcellular localization of the HA epitope-tagged protein used in the traditional CHRAP assay would not be altered by the epitope, we performed the indirect immunofluorescence microscopic analysis (Figure 5(c)). Clearly, the HA-tagged proteins remained to be nuclear. Hence, we conclude that the SILAC enriched nucleus-localized proteins are CHRAP candidates.

Some of the dual-localized proteins were found in the top ranked proteins by SILAC ratios (see Supplementary Table S2). To test if they were the true CHRAPs, the 3 randomly selected proteins Uve1, Hsp16, and C530.05 were subjected to the traditional CHRAP assay. The analysis indicated that all 3 proteins exhibited apparent enrichment in the CHRAP-prep when compared to WCE (Figure 5(d)). On the other hand, the presence in the soluble fraction was detected in 2 out of 3 dual-localized proteins, consistent with their dual subcellular localization. This result indicates that most of the top ranked proteins by SILAC ratios are true CHRAP candidates.

Of the 507 proteins identified in the SILAC analysis, 413 were found to be either SILAC enriched (i.e., log 2 SILAC-ratio > 0.585) or depleted (i.e., log 2 SILAC-ratio < −0.585). We wanted to test if the SILAC-enriched proteins have a likelihood of requirement for growth fitness in DNA damage stress, one of the CHRAP-associated functions. For this reason, 188 (~45.5%) *S. pombe* gene deletion strains from the Bioneer deletion strains set (version 1) were subjected to the phenotypic assessment using the mini-growth curve assay [24]. Level of growth fitness under MMS stress was estimated by the growth fitness score  (GFS_MMS_)  that was calculated based on the difference of  *T*_50_ (the time at the half-maximal concentration) between cultures supplemented with and without MMS (see Section 4).

**Figure 6:**
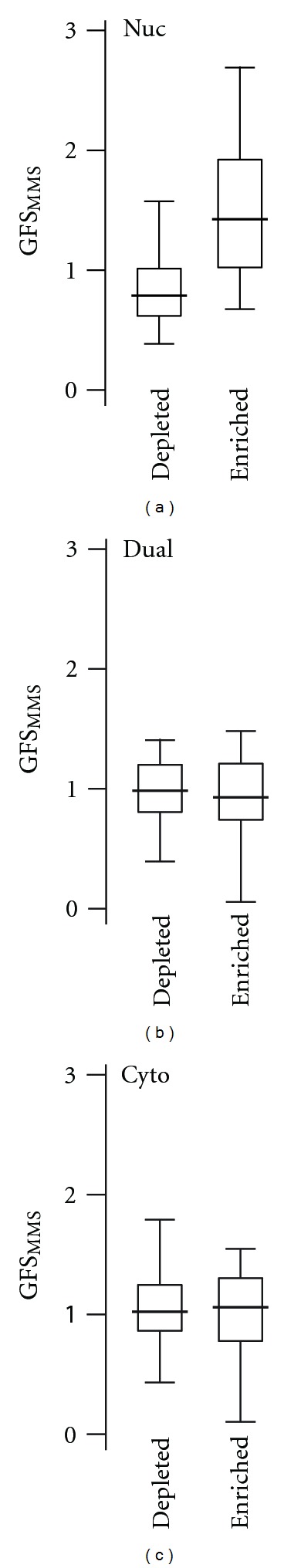
SILAC-enriched nucleus-localized proteins exhibit the high likelihood of requirement for growth fitness under DNA damage stress. The boxplots show the level of growth fitness in various mutant strains. Cells containing a deletion allele of the SILAC-enriched or depleted proteins are indicated. Nucleus-localized (Nuc), dual-localized (Dual), or cytoplasm-localized (Cyto) are shown in (a), (b), or (c), respectively.

The growth fitness score GFS_MMS_ was proportional to the level of requirement for growth fitness under MMS stress. It was apparent that, among the nucleus-localized proteins, the median GFS_MMS_ of the SILAC-enriched proteins was significantly higher than that of the SILAC-depleted ones (1.43 versus 0.79; *P* value < 0.05; Supplementary Table S4). This result indicates that the top SILAC-ranked nucleus-localized proteins are *bona fide* CHRAP candidates that exhibit a high likelihood of requirement for growth fitness under DNA damage stress (Figure 6(a)). On the other hand, among the cytoplasm-localized or dual-localized proteins, the median GFS_MMS_ of the SILAC-enriched proteins showed no apparent differences from that of the SILAC-depleted ones (i.e., 0.93 versus 0.98 or 1.05 versus 1.02) (Figures 6(b) and 6(c)). This is consistent with the observation that the cytoplasm-localized or dual-localized proteins were not overrepresented in the top ranked proteins by SILAC ratios (see Figure 4(a)). Hence, SILAC proteomic analysis of CHRAP-prep is capable of identifying CHRAP candidates without prior knowledge. 

We noted that, however, hardly any chromo-domain or bromo-domain containing chromatin remodelers are found in our SILAC analysis of CHRAP-prep. This is probably a result of using the physiological salt concentration in this analysis (see Section 4). It is known that extraction of chromo-domain and bromo-domain proteins requires high salt concentrations [25]. Alternatively, these proteins could also escape the detection in LC-MS/MS due to their relatively low abundance and high level of interference from the cytoplasm-localized abundant proteins. It could be improved by better chromatographic separation of the trypsinized peptides using a longer column or UPLC separation before mass spectrometric analysis. Modification of CHRAP preparation and improvement of SILAC proteomic analysis should allow identification of the chromo-domain and bromo-domain proteins in future studies.

## 3. Conclusion

We show that the CHRAP-prep used in traditional assays for CHRAPs is predominated by the abundant cytoplasmic proteins such as ribosomal proteins based on the absolute abundance of proteins. On the other hand, we show that proteomic analysis of CHRAP-prep together with the SILAC-labeled WCE is able to effectively deplete the ribosomal proteins from the top ranked proteins by SILAC ratios. Significantly, we show that the top ranked proteins by SILAC ratios enrich for nucleus-localized proteins that display a high likelihood of requirement for growth fitness under DNA damage stress. Hence, the SILAC-mediated proteomic analysis is capable of determining CHRAPs without prior knowledge. We propose the method shown in this study can be complementary to the proteomic analysis of protein complexes purified via ChIP with TAP-tagged CHRAPs for identification of CHRAP-interacting partners.

## 4. Methods

### 4.1. Strain Construction and Cell Culture Manipulation

Strains used in this study are listed in Table 1 except for Bioneer deletion strains (Bioneer Corporation, Daejeon, Korea). The strain *lys1-131* was used in preparation of the SILAC-labeled cells. Hemagglutinin (HA)-tagged strains for western blot analysis were constructed based on the protocol reported previously [26]. The Bioneer deletion strains used in this study are listed in Supplementary Table S1. Cultures in minimal medium (MM) supplemented with normal or heavy lysine (^13^C_6_-lysine; Cat. No. CLM-2247-0.25; Cambridge Isotope Laboratories, Andover, MA, USA) was used in proteomic analysis. Cultures in rich medium (YES) supplemented with or without methyl methanesulfonate (MMS) at the final concentration of 1 mM were used in growth fitness assays.

### 4.2. Enrichment of Chromatin-Associated Proteins (CHRAPs)

To enrich the CHRAPs, CHRAP-prep was obtained as described elsewhere with some modification [13, 14]. In brief, ~500 mL (for LC-MS/MS analysis) or 50–100mL (for western blot analysis) log-phase growth cells (OD_600_ = ~0.8)  were harvested and washed once with STOP Buffer (0.9% NaCl, 1 mM NaN_3_, 50 mM NaF, 10 mM EDTA). The washed cells were then protoplasted by resuspending in protoplast buffer (35.5 mM BME, 50 mM sodium citrate, 40 mM EDTA, and 1.2 M sorbitol) supplemented with 8 mg/mL zymolyase-20T (MP Biomedicals Inc., Solon, OH, USA). Protoplasting of cells were monitored frequently under microscope (protoplasts turned dark when treated with 1% SDS). The reaction was stopped by addition of an equal volume of ice-cold 1.2 M sorbitol pH 7.5 when ~90% of the cells were protoplasted. The washed protoplasts were resuspended in 450 *μ*L 1.2 M sorbitol for generating CHRAPs-prep or snap-frozen in liquid nitrogen and stored at −80°C for later use. The resuspended protoplasts were lysed by addition of 50 *μ*L 10x lysis buffer (500 mM KAc, 20 mM MgCl_2_, 200 mM HEPES pH 7.9) supplemented with the 1x complete protease inhibitors EDTA-free tablet (Roche, Basel, Switzerland) and 1% Triton X-100 (TX-100) (Promega, Fitchburg, WI, USA). The lysate was incubated on ice for 10 min with occasional mixing. Ten percent of the lysate was preserved as whole cell extract (WCE). The remaining was centrifuged at 12,000 g for 15 min at 4°C. Supernatant (sup-1) containing soluble proteins was transferred to a fresh tube and the pellet (pel-1) was washed twice and resuspended in lysis buffer without TX-100 to yield the crude chromatin extract. The resulting crude chromatin fraction was digested with DNaseI (Stratagene, La Jolla, CA, USA) at the concentration of 10 unit/*μ*g DNA in digestion buffer (40 mM Tris-HCl pH 7.5, 6 mM MgCl_2_, 150 mM NaCl, 2 mM CaCl_2_ and protease inhibitors) at 37°C for 30 min with vigorous shaking.The DNaseI-digested crude chromatin extract was centrifuged at 14,000 g for 5 min. The supernatant (sup-2) was referred as the CHRAP-prep and separated from the pellet (pel-2).

### 4.3. SDS-PAGE and Western Blot Analysis

A desired amount of proteins was taken and mixed with standard loading buffer for SDS-PAGE analysis. Proteins in gel were electrotransferred onto nitrocellulose membranes for probing with primary antibodies against HA (Santa Cruz Biotechnology, Santa Cruz, CA, USA), histone H4 (Upstate Biotechnology, Lake Placid, NY, USA), and **β**-tubulin (TAT1 antibody; a gift of K. Gull, University of Oxford, London, UK). Secondary antibodies conjugated with horse radish peroxidase (GE Healthcare, Piscataway, NJ, USA) were used for detection of chemiluminescence signals using the ECL Plus System (GE Healthcare).

### 4.4. Sample Preparation and LC-MS/MS Analysis

Prior to MS analysis, protein samples were fractionated in SDS-PAGE gels. Gels were sliced into ~50 pieces from top to bottom of a lane. Proteins in gel slices were destained and trypsinized in-gel in 25 mM NH_4_HCO_3_ supplemented with 12.5 ng/*μ*L trypsin (Promega, Madison, WI, USA). The resulting peptides were cleaned using C18 ZipTip (Millipore, Medford, MA, USA) and ready for mass spectrometry analysis.

Mass spectrometry analysis was performed using a nanoflow high-performance liquid chromatography (HPLC) system (Eksigent, Dublin, CA) connected to a hybrid LTQ-Orbitrap (Thermo Scientific, Bremen, Germany) equipped with a nanoelectrospray ion source (Thermo Scientific). The peptides were separated with a 15 cm long and 75 *μ*m inner diameter PicoFrit column with an integrated tip (New Objective Inc, Woburn, MA) packed with 4 *μ*m reverse-phase C12 resins (Jupiter Proteo Phenomenex, Torrance, CA, USA). HPLC mobile phase consists of (A) 2% acetonitrile 0.1% formic acid and (B) 98% acetonitrile 0.1% formic acid. Approximately 10 *μ*L peptide solution was loaded onto a nano trap column (300SB-C18, Agilent) with 100% mobile phase A and washed for 10 min at a flow rate of 20 *μ*L/min. The trap column was then brought in-line with the nano column using the CN2 nano volume switching valve (VICI Valco Cheminert, Switzerland) and the peptides were eluted by 2–35% mobile phase B over 70 min and 35–90% over 6 min with a constant flow rate of 300 nL/min. Finally the column was washed for 10 min with high concentration of organic solvent (90% mobile phase B) and re-equilibrate with another 15 min with 98% mobile-phase A prior to loading of the next sample. Eluted peptides from HPLC column were directly electrosprayed into the LTQ-Orbitrap mass spectrometer for analysis. The spray voltage was set to 2.0 kV and the temperature of the heated capillary was set to 250°C. The MS instrument was operated in a data-dependent mode by automatically switching between the full survey scan and MS/MS acquisition. High resolution precursor spectra (*m/z* 300–2,000) were acquired in the Orbitrap with resolution of 60,000 at *m/z* 400 (after accumulation to a target value of 10^6^ ions in the linear ion trap). The 5 most intense ions with ion intensity above 1,000 counts and charged state  ≥2  were sequentially isolated for fragmentation in the linear ion trap using collision induced dissociation (CID; normalized collision energy 35%, activation Q 0.250, and activation time 30 ms) at a target value of 10,000 ions. The dynamic exclusion list was restricted to a maximum retention period of 90 sec and a relative mass window of 10 ppm. The MS and MS/MS spectra were recorded by the mass spectrometer as raw files using the Xcalibur software 2.0SR2 (Thermo Fisher Scientific).

### 4.5. SILAC-Mediated Proteomic Data Analysis

TPP (Trans-Proteomic Pipeline version 4.2.1, Seattle Proteome Centre, Institute of Systems Biology, Seattle, WA, USA) was used to perform database searching and peptide assignment and validation. For this purpose, all RAW spectra files were converted to mzXML-format. Uninterpreted MS/MS spectra were searched against the *S. pombe* protein databases UniProt Knowledgebase, including Swiss-Prot and TrEMBL using SEQUEST algorithm [27]. The database includes forward protein (target) and reverse protein (decoy) sequences that were generated by Bioworks 3.3.1 (Thermo Scientific). Search parameters used in this study were the requirement of tryptic cleavage (allowing 1 missed cleavage site), minimum peptide length of 7 amino acids, maximal precursor ions mass deviation of 10 ppm, peptide mass tolerance of ±0.5 Da, static modification on Cys of  +57.0215 Da, differential modification on Met of 15.9945 Da, and heavy isotope coupling on Lys of  +6.0201 Da. The output was in pepXML-format. All assigned peptides were validated using PeptideProphet [28] and the cutoff was set to the probability of 90% or greater. The qualified peptides (probability ≥ 90%) were quantified by the XPRESS software [29]. The elution profile of the ^12^C_6_-Lys and ^13^C_6_-Lys containing peptides from the qualified peptides were isolated and quantified based on the area of peaks by XPRESS. Protein levels were approximated by the median level of unique peptides. A total of 507 proteins with a ratio between light (CHRAP-prep) and heavy (WCE) protein levels were listed in Supplementary Table S2.

### 4.6. Non-SILAC Proteomic Data Analysis

To test whether the CHRAP candidates (e.g., nucleus-localized proteins) could be dominated in the most abundant proteins (i.e., based on the absolute abundance) in the CHRAP-prep, proteomic analysis of the CHRAP-prep proteins without SILAC was performed. In determination of the absolute abundance, the PeptideProphet-qualified peptides (probability ≥ 90%) were quantified by PepQuan and the abundance of proteins was estimated by the median abundance of the respective unique peptides. A total of 376 proteins whose abundance level was approximated by the median level of their unique peptides were listed in Supplementary Table S3. Based on the absolute abundance, nucleus-localized proteins were not overrepresented in the top 10% most abundant proteins (Supplementary Figure S1). Notably, on the other hand, the ribosomal proteins were dominated in the top 10% most abundant proteins, suggesting that the highly abundant non-CHRAP ribosomal proteins are not effectively depleted in the CHRAP-prep.

### 4.7. Miniculture Growth Curve Assay

Of 507 proteins identified in the SILAC-mediated analysis, 413 were either enriched or depleted (i.e., ratio ≥ ±1.5 fold) in CHRAP-prep when compared to WCE. Out of 413 deletion strains, 188 (45.5%) were found in the Bioneer deletion strain collection (Version 1, Bioneer Corporation). Therefore, 188 deletion mutant strains (see Supplementary Table S1) were subjected to minigrowth curve assays using the Bioscreen miniculture growth curve system (Growth Curves USA, Piscataway, NJ) for growth fitness in 1 mM MMS stress. All tests were done in triplicate. Minigrowth curve assay settings used were identical to the previous study [24].

Growth fitness score in MMS (GFS_MMS_) is calculated based on the formula (GFS_MMS_) = 100(Δ*T*_50_mut_/Δ*T*_50_wt_),  in which *T*_50_ is the time at the half-maximal cell concentration based on the growth curve, and Δ*T*_50_ is the difference of the half-maximal time between cultures supplemented with and without MMS. mut stands for mutant strains and wt for the wild type strain. The average GFS_MMS_ of tested strains is listed in Supplementary Table S4.

### 4.8. Statistical Analysis

Binomial test was used to test the nonrandom distribution. In analysis of enrichment or depletion of the nuclear or cytoplasmic proteins, the protein localization information in a genome-wide study by Matsuyama et al. [19] was used, in which 4,387 proteins whose subcellular localization has been characterized. Localization of additional 321 proteins is based on the GO terms (i.e., cellular components; http://www.genedb.org/ or http://amigo.geneontology.org/). The subcellular localizations are briefly categorized into three types: the nucleus (it includes the nucleus, nucleolus, spindle pole body, and nuclear envelope), cytoplasm (it includes cytosol, mitochondrion, Golgi, ER, cytoskeleton, plasma membrane, and other cytoplasmic organelles), or both. Thus, 4,708 proteins (i.e., ~93% of *S. pombe* proteome) have well defined localization types: 763 (~16.2%) are localized at the nucleus, 2180 (46.3%) at the cytoplasm, and 1765 (37.5%) at both the nucleus and cytoplasm.

Biological process terms were only considered in gene ontology analysis when the occurrence was 15 or greater in the group of 300–500 proteins.

Unpaired two-sample *t*-test was used to test the difference of GFS_MMS_ between the SILAC-enriched and depleted proteins with nucleus or cytoplasm or both localizations.

Original mass spectrometric data are deposited in Tranche database (https://proteomecommons.org/) and can be accessed by a hash “ap/3Mk3fjF/4aY8SslwNzqzdi9FDeoAWmmB9oZ3th0cnj807UrbPmSG/s+HWzM4MAzdrmmMhQxi10xe5jO2jaQtfSsEAAAAAAAAEMQ==” or at the author's website at http://pombe.gis.a-star.edu.sg/. 

## Supplementary Material

Figure S1: Non-SILAC proteomic analysis of CHRAP-prep.
Table S1: List of the Bioneer deletion strains used in this study.
Table S2: List of proteins and their SILAC-ratio in CHRAP-prep versus WCE.
Table S3: List of proteins and their abundances in CHRAP-prep.
Table S4: List of growth fitness scores of deletion strains tested under MMS stress.

## Figures and Tables

**Table 1:** List of strains used in this study^a^.

ID	Relevant genotype	Comment
LJY3766	*lys1-131 ura4-D18h-*	Laboratory stock
LJY188	*leu1-32 ura4-D18h-*	Laboratory stock
LJY4383	*hsp16* ^ +^ *-3HA-6His::ura4+ ura4-D18 leu1-* *32 h-*	This study
LJY4384	*1c8.05c* ^ +^ *-3HA-6His::ura4+ ura4-D18 leu1-* *32 h-*	This study
LJY4385	*msh3* ^ +^ *-3HA-6His::ura4+ ura4-D18 leu1-* *32 h-*	This study
LJY4386	*uve1* ^ +^ *-3HA-6His::ura4+ ura4-D18 leu1-* *32 h-*	This study
LJY3236	*c530.05* ^+^ *-3HA-6His::ura4+ ura4-D18 leu1-* *32 h-*	This study
LJY4224	*prp16* ^ +^ *-3HA-6His::ura4+ ura4-D18 leu1-* *32 h-*	This study
LJY4429	*kap95* ^ +^ *-HA-6His::ura4+ ura4-D18 leu1-* *32 h-*	This study
LJY4430	*srp2* ^ +^ *-HA-6His::ura4+ ura4-D18 leu1-* *32 h-*	This study

Note: ^a^Bioneer deletion strains used in this study are listed in the Supplementary Table S1.

## List of Abbreviations

ChIP: Chromatin immunoprecipitation
CHRAP: Chromatin-associated non-histone proteins
CHRAP-prep: Preparation of proteins enriched for CHRAPs
GFS: Growth fitness score
GO: Gene ontology
LC: Liquid chromatography
LTQ: Linear trap quadrupole
MS/MS: Tandem mass spectrometry
SILAC: Stable isotope labeling with amino acids in cell culture
WCE: Whole cell extract.
