# Supplementary material for: Chromatin-Associated Proteins Revealed by SILAC-Proteomic Analysis Exhibit a High Likelihood of Requirement for Growth Fitness under DNA Damage Stress
Source: Int J Proteomics. 2012 Aug 1;2012:630409. doi: 10.1155/2012/630409 (PMC3415198; doi:10.1155/2012/630409)
Supplement: Supplementary file 1 — Figure S1: Non-SILAC proteomic analysis of CHRAP-prep. Table S1: List of the Bioneer deletion strains used in this study. Table S2: List of proteins and their SILAC-ratio in CHRAP-prep versus WCE. Table S3: List of proteins and their abundances in CHRAP-prep. Table S4: List of growth fitness scores of deletion strains tested under MMS stress. [file 630409.f1.pdf]

Supplemental Materials

Chromatin-associated proteins revealed by SILAC-proteomic analysis exhibit a high likelihood of requirement for growth fitness under DNA damage stress

Han Wang,[1†] Pornpimol Tipthara,[2†] Lei Zhu,[2] Suk Yean Poon,[2] Kai Tang,[1*] and Jianhua Liu[2,3*]

[1]School of Biological Science, Nanyang Technological University

[2]Systems Biology, Genome Institute of Singapore

[3]Department of Biochemistry, Yong Loo Lin School of Medicine, National University of Singapore

[†]These authors contributed equally (HW, PT)

[*]Corresponding authors (TK, JL)

Send all correspondence to:

Jianhua Liu, Group Leader, Systems Biology, Genome Institute of Singapore, 60 Biopolis street #02-01, Singapore 138672, Tel: (65) 6808 8123, Fax: (65) 6808 8306, Email: liujh@gis.a-star.edu.sg

Email addresses of all authors

Han Wang	S070057@e.ntu.edu.sg
Pornpimol Tipthara	tiptharap@gis.a-star.edu.sg
Lei Zhu	zhul@gis.a-star.edu.sg
Suk Yean Poon	poonsy@gis.a-star.edu.sg
Kai Tang	ktang@pmail.ntu.edu.sg
Jianhua Liu	liujh@gis.a-star.edu.sg

List of supplemental files

Supplemental figure S1. Non-SILAC proteomic analysis of CHRAP-prep.

Supplemental table S1. List of the Bioneer deletion strains used in this study.

Supplemental table S2. List of proteins and their SILAC-ratio in CHRAP-prep.

Supplemental table S3. List of proteins and their abundances in CHRAP-prep.

Supplemental table S4. List of growth fitness scores of deletion strains tested in MMS stress.

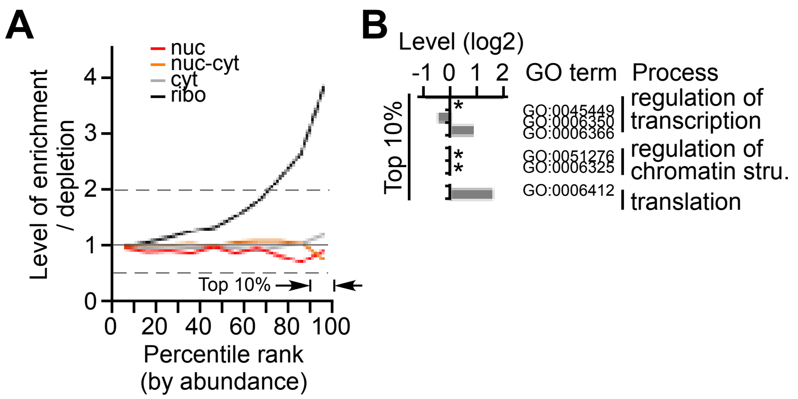

Figure S1. Analysis of CHRAP-prep via non-SILAC throughput proteomic analysis. (A) Ribosomal proteins are overrepresented in the top 10% most abundant proteins in CHRAP-prep. X-axis indicates the percentile ranks by protein abundance. Enrichment of various types of proteins indicated is based on the observed number over the background level. (B) Gene ontology analysis of the top10% abundant proteins in CHRAP-prep. Asterisk indicates the occurrence (i.e., < 5) of the relevant GO-term associated genes is too low to perform statistic analysis.

Supplemental Table S1. The list of Bioneer gene deletion strains used in this study

Order	Relevant genotype	Order	Relevant genotype	Order	Relevant genotype
1	urg3::KanR	64	pabp::KanR	127	hmt2::KanR
2	SPAC1071.02::KanR	65	adh4::KanR	128	eri1::KanR
3	get3::KanR	66	SPAC607.06c::KanR	129	SPBC30D10.05c::KanR
4	swr1::KanR	67	acp2::KanR	130	pdb1::KanR
5	rpl32::KanR	68	cit1::KanR	131	rpl12::KanR
6	SPAC13A11.06::KanR	69	SPAC6C3.08::KanR	132	gpx1::KanR
7	rps11::KanR	70	idp1::KanR	133	tif512::KanR
8	gcv2::KanR	71	ndk1::KanR	134	cho1::KanR
9	rps61::KanR	72	SPAC821.05::KanR	135	SPBC365.01::KanR
10	ssa1::KanR	73	dmc1::KanR	136	SPBC3B8.03::KanR
11	ade2::KanR	74	SPAC8E11.10::KanR	137	hsp16::KanR
12	SPAC1486.01::KanR	75	msh3::KanR	138	leu3::KanR
13	atp1::KanR	76	fas1::KanR	139	SPBC3F6.01c::KanR
14	sdh1::KanR	77	rps43::KanR	140	ade7::KanR
15	cdd1::KanR	78	SPAC977.14c::KanR	141	eca39::KanR
16	pmm1::KanR	79	SPAC9G1.05::KanR	142	arg12::KanR
17	SPAC15A10.07::KanR	80	SPAC9G1.10c::KanR	143	meu6::KanR
18	SPAC1687.21::KanR	81	SPACUNK4.10::KanR	144	SPBC428.11::KanR
19	SPAC17G6.15c::KanR	82	SPACUNK4.16c::KanR	145	SPBC530.05::KanR
20	SPAC17H9.14c::KanR	83	tpp2::KanR	146	utp2::KanR
21	cki3::KanR	84	SPAPB24D3.08c::KanR	147	pob3::KanR
22	shm2::KanR	85	lys4::KanR	148	sds23::KanR
23	rps7::KanR	86	SPBC1198.08::KanR	149	SPBC713.03::KanR
24	SPAC19G12.09::KanR	87	mrt4::KanR	150	ura5::KanR
25	plb1::KanR	88	spf38::KanR	151	ste13::KanR
26	kap95::KanR	89	SPBC12C2.07c::KanR	152	atb2::KanR
27	wis2::KanR	90	dnm1::KanR	153	rpl432::KanR
28	SPAC1F12.02c::KanR	91	glo1::KanR	154	hhf2::KanR
29	mug161::KanR	92	fkbp39::KanR	155	SPBC8D2.18c::KanR
30	fio1::KanR	93	SPBC13G1.02::KanR	156	SPBC947.08c::KanR
31	SPAC20G8.09c::KanR	94	myo1::KanR	157	ilv1::KanR
32	lys3::KanR	95	kap123::KanR	158	SPBP4H10.12::KanR
33	dak1::KanR	96	pho2::KanR	159	rps232::KanR
34	SPAC22A12.16::KanR	97	rps122::KanR	160	rpp21::KanR
35	rps12::KanR	98	SPBC16A3.02c::KanR	161	SPBP8B7.11::KanR
36	SPAC23A1.17::KanR	99	vps132::KanR	162	cyp4::KanR
37	cdb4::KanR	100	qcr6::KanR	163	gpt1::KanR
38	SPAC24H6.11c::KanR	101	SPBC16D10.08c::KanR	164	SPCC1183.02::KanR
39	his1::KanR	102	SPBC16G5.05c::KanR	165	nmt1::KanR
40	SPAC26H5.02c::KanR	103	bag11::KanR	166	rps182::KanR
41	SPAC27D7.06::KanR	104	rps3::KanR	167	SPCC1281.07c::KanR
42	SPAC27F1.06c::KanR	105	sks2::KanR	168	SPCC132.01c::KanR
43	SPAC29A4.02c::KanR	106	SPBC1711.08::KanR	169	rpl233::KanR
44	pom1::KanR	107	his2::KanR	170	SPCC1450.12::KanR
45	SPAC2F7.09c::KanR	108	SPBC17D1.07c::KanR	171	arz1::KanR
46	SPAC30C2.04::KanR	109	rga5::KanR	172	SPCC16C4.10::KanR
47	gef2::KanR	110	tif213::KanR	173	SPCC1827.03c::KanR
48	rps111::KanR	111	rpl112::KanR	174	bgs4::KanR
49	SPAC31G5.04::KanR	112	ape2::KanR	175	SPCC1840.09::KanR
50	gcv1::KanR	113	ubr1::KanR	176	SPCC18B5.05c::KanR
51	tps1::KanR	114	uve1::KanR	177	SPCC191.02c::KanR
52	SPAC32A11.02c::KanR	115	leu1::KanR	178	SPCC24B10.18::KanR
53	SPAC3A11.07::KanR	116	SPBC1E8.03c::KanR	179	rpl122::KanR
54	rpl21::KanR	117	arg4::KanR	180	SPCC330.06c::KanR
55	SPAC3C7.07c::KanR	118	SPBC215.11c::KanR	181	SPCC338.14::KanR
56	obr1::KanR	119	rad3::KanR	182	nap1::KanR
57	abc2::KanR	120	car2::KanR	183	SPCC584.15c::KanR
58	rpl72::KanR	121	SPBC21C3.19::KanR	184	snd1::KanR
59	spo2::KanR	122	his5::KanR	185	lat1::KanR
60	isp6::KanR	123	egd2::KanR	186	ef1a-a::KanR
61	itr1::KanR	124	sua1::KanR	187	rps121::KanR
62	rpl91::KanR	125	fib1::KanR	188	ade1::KanR
63	SPAC513.07::KanR	126	clr1::KanR		

Supplemenal Table S2. Relative abundance (ratio) of proteins in CHRAP-prep versus WCE

Uniprot ID	Sysname|symbol	Rank[a]	Ratio (log2)[b]	# of unique peptide[c]	# of spectrum[d]	Localization (Matsuyama et al, 2006)[e]	Localization (GO terms)[f]	Localization (combined)[g]	nuc[h]	cyt; nuc[i]	cyt[j]	ribo[k]	GO:0045449[l]	GO:0051276[m]	GO:0006260[n]	GO:0006350[o]	GO:0006281[p]	GO:0016568[q]	GO:0006325[r]	GO:0006355[s]	GO:0006366[t]	GO:0031497[u]	GO:0006412[v]	product[w]
P00332	SPCC13B11.01|adh1	1	7.94	14	470	NA	cyt	cyt			1													alcohol dehydrogenase Adh1
Q9Y7T3	SPCC63.07	2	7.88	1	3	cyt; nuc	cyt; nuc	cyt; nuc		1														tRNA guanylyltransferase (predic
O42649	SPAC10F6.09c|psm3	3	6.64	1	1	cyt; nuc	cyt; nuc	cyt; nuc		1				1										mitotic cohesin complex subunit
Q08I43	SPBC30B4.08|eri1	4	6.54	1	1	cyt	cyt	cyt			1		1			1				1				double-strand siRNA ribonucleas
Q8TFF9	SPBPB8B6.03	5	6.50	1	4	cyt	cyt	cyt			1													acetamidase (predicted)
O13807	SPAC17H9.10c|ddb1	6	6.10	1	1	cyt; nuc	cyt; nuc	cyt; nuc		1					1		1							damaged DNA binding protein D(
P26359	SPAC8F11.03|msh3	7	5.86	1	1	nuc	nuc	nuc	1					1			1							MutS protein homolog 3
O14215	SPAC6B12.10c|spp1	8	5.73	1	1	nuc	nuc	nuc	1					1	1									DNA primase catalytic subunit Sp
O42854	SPAC23A1.17	9	5.52	2	2	cyt	cyt	cyt			1													WIP (Wiskott-Aldrich syndrome p
O60108	SPBC14F5.12c|cbh2	10	5.17	1	1	cyt	nuc	cyt			1		1	1		1		1	1	1				CENP-B homolog Cbh2
Q9USM6	SPCC16A11.10c|oca8	11	4.91	1	5	cyt	cyt	cyt			1													cytochrome b5 (predicted)
Q10201	SPBC17D1.07c	12	4.91	1	1	nuc	nuc	nuc	1															GTPase regulator (predicted)
O43083	SPBC947.08c	13	4.90	1	1	nuc	nuc	nuc	1				1	1		1		1	1	1	1	1		histone promoter control proteir
Q10988	SPBC19C7.09c|uve1	14	4.83	1	1	cyt; nuc	cyt; nuc	cyt; nuc		1							1							endonuclease Uve1
P36594	SPBC28F2.12|rpb1	15	4.78	3	7	cyt; nuc	cyt; nuc	cyt; nuc		1						1					1			RNA polymerase II large subunit
Q9P774	SPBC1711.17|prp16	16	4.72	1	3	nuc	nuc	nuc	1															ATP-dependent RNA helicase Prp
Q9UUK7	SPCC569.08c|ade5	17	4.60	1	1	cyt; nuc	nuc	cyt; nuc		1														phosphoribosylglycinamide form
Q09140	SPBPJ4664.06|gpt1	18	4.54	1	27	ND	cyt	cyt			1													UDP-glucose-glycoprotein glucos
Q10286	SPAC4F8.15|itr1	19	4.51	1	2	cyt	cyt	cyt			1													myo-inositol transporter Itr1
Q9UT05	SPAP8A3.12c|tpp2	20	4.51	2	3	ND	cyt	cyt			1													tripeptidyl-peptidase II Tpp2
P27584	SPBC24C6.06|gpa1	21	4.50	1	1	cyt	cyt	cyt			1													G-protein alpha subunit
Q10163	SPAC26A3.08|smb1	22	4.46	1	2	cyt; nuc	cyt; nuc	cyt; nuc		1														Sm snRNP core protein Smb1
O94265	SPBP8B7.17c	23	4.44	1	1	cyt; nuc	cyt; nuc	cyt; nuc		1														TENA/THI family protein
O74429	SPCC1672.06c|asp1	24	4.44	1	1	cyt; nuc	cyt; nuc	cyt; nuc		1														inositol hexakisphosphate kinase
Q09737	SPAC13A11.06	25	4.34	1	2	cyt; nuc	cyt; nuc	cyt; nuc		1														pyruvate decarboxylase (predicte
O42870	SPAC3G9.06|frs2	26	4.28	1	1	cyt	cyt	cyt			1												1	cytoplasmic phenylalanine-tRNA
O94486	SPCC417.05c|chr2	27	4.26	2	2	cyt; nuc	cyt; nuc	cyt; nuc		1														chitin synthase regulatory factor
O74855	SPCC18.05c	28	4.19	1	1	nuc	nuc	nuc	1															notchless-like protein
P15398	SPBC4C3.05c|nuc1	29	4.14	7	14	cyt	cyt; nuc	cyt			1					1								DNA-directed RNA polymerase I (
O14368	SPBC3E7.02c|hsp16	30	4.12	4	20	cyt; nuc	cyt; nuc	cyt; nuc		1														heat shock protein Hsp16

Q10185	SPAC3F10.11c|abc2	31	4.08	1	4	cyt	cyt	cyt			1							glutathione S-conjugate-exportin
P41887	SPAC926.04c|hsp90	32	3.97	23	220	cyt	cyt	cyt			1		1	1	1			Hsp90 chaperone
O60152	SPBC19C7.02|ubr1	33	3.95	1	1	NA	nuc	nuc	1									N-end-recognizing protein Ubr1
O94260	SPBP8B7.11	34	3.91	1	1	cyt	cyt	cyt			1							ubiquitin protease cofactor (prec
O59741	SPBC530.05	35	3.90	1	1	cyt; nuc	cyt; nuc	cyt; nuc		1			1	1	1	1		transcription factor (predicted)
Q10488	SPAC26F1.04c|etr1	36	3.79	1	1	cyt	cyt	cyt			1							enoyl-[acyl-carrier protein] reduc
Q9P7E0	SPBP4H10.08|qcr1	37	3.79	1	1	cyt	cyt	cyt			1							Reiske ISP-associated protein, ub
O13793	SPAC17G6.15c	38	3.76	1	1	cyt	cyt	cyt			1							MTC tricarboxylate transporter
O74475	SPCC1840.02c|bgs4	39	3.76	1	1	cyt	cyt	cyt			1							1,3-beta-glucan synthase subunit
O60081	SPCC1494.07	40	3.63	2	2	cyt; nuc	cyt; nuc	cyt; nuc		1								human THADA ortholog
P40921	SPAC29A4.02c	41	3.62	12	65	cyt	cyt	cyt			1						1	translation elongation factor EF-1
Q10414	SPAC1F3.09|mug161	42	3.54	1	1	nuc	nuc	nuc	1									CwfJ family protein, splicing facto
O14062	SPCC962.04|rps121	43	3.52	1	11	NA	cyt	cyt			1	1					1	40S ribosomal protein S12
Q9UT32	SPAC8F11.04	44	3.43	4	5	nuc	nuc	nuc	1			1					1	U3 snoRNP-associated protein Ci
Q9Y7Z8	SPBC146.13c|myo1	45	3.32	7	8	cyt	cyt	cyt			1							myosin type I
Q10175	SPAC27F1.06c	46	3.26	1	1	nuc	nuc	nuc	1									FKBP-type peptidyl-prolyl cis-trar
O42936	SPBC16C6.12c	47	3.25	1	1	nuc	nuc	nuc	1									Las1-like protein
O94676	SPBC776.08c	48	3.25	1	1	nuc	nuc	nuc	1									Nrap
Q9UTP0	SPAC1250.05|rpl32	49	3.23	2	3	cyt	cyt	cyt			1	1					1	60S ribosomal protein L30
P36612	SPBC4.07c|rpt2	50	3.17	1	1	cyt; nuc	cyt; nuc	cyt; nuc		1								19S proteasome regulatory subu
O94686	SPBC83.02c|rpl432	51	3.15	1	1	cyt	cyt	cyt			1	1					1	60S ribosomal protein L37a (prec
Q09916	SPAC1F7.02c|has1	52	2.98	2	3	nuc	nuc	nuc	1									ATP-dependent RNA helicase Has
P36597	SPCC1223.02|nmt1	53	2.94	1	1	cyt; nuc	cyt; nuc	cyt; nuc		1								no message in thiamine Nmt1
P78820	SPAC56E4.04c|cut6	54	2.93	6	8	cyt	cyt	cyt			1							acetyl-CoA/biotin carboxylase
O74322	SPBC1685.02c|rps122	55	2.93	1	6	cyt; nuc	cyt; nuc	cyt; nuc		1		1					1	40S ribosomal protein S12 (predi
Q9USI5	SPCC645.14c|sti1	56	2.84	1	1	cyt; nuc	cyt; nuc	cyt; nuc		1								chaperone activator Sti1 (predict
Q9P7S7	SPAC23G3.06	57	2.79	4	11	nuc	nuc	nuc	1									U3 snoRNP protein Nop58 (predi
O59816	SPCC794.07|lat1	58	2.65	3	8	cyt	cyt	cyt			1							dihydrolipoamide S-acetyltransfe
Q9UT76	SPAC343.14c	59	2.64	1	1	cyt; nuc	cyt	cyt; nuc		1							1	translation initiation factor eIF2B
Q02061	SPAC23G3.01|rpb2	60	2.60	7	9	cyt; nuc	cyt; nuc	cyt; nuc		1			1	1	1	1		RNA polymerase II complex subu
O94238	SPAC1805.13|rpl14	61	2.56	3	79	cyt; nuc	cyt; nuc	cyt; nuc		1		1					1	60S ribosomal protein L14 (predi
Q09764	SPAC24H6.11c	62	2.51	1	1	cyt	cyt	cyt			1							sulfate transporter (predicted)
Q9UTH5	SPAC1786.02	63	2.43	1	2	cyt	cyt	cyt			1							phospholipase (predicted)
Q9P377	SPAC19B12.03|bgs3	64	2.42	1	3	cyt		cyt			1							1,3-beta-glucan synthase subunit
Q9UTM4	SPAC1420.02c|cct5	65	2.32	2	4	cyt	cyt	cyt			1							chaperonin-containing T-complex
Q9URX2	SPAC922.04	66	2.32	1	5	cyt	cyt	cyt			1							sequence orphan
P36621	SPCC306.09c|cap1	67	2.30	5	10	cyt	cyt	cyt			1							adenylyl cyclase-associated prote
P87177	SPBC3D6.12	68	2.28	1	1	cyt; nuc	cyt; nuc	cyt; nuc		1								U3 snoRNA associted protein Dip
O94514	SPBC646.10c	69	2.27	1	1	nuc	nuc	nuc	1									U3 snoRNP protein Nop56 (predi
O14363	SPBC30D10.18c|rpl12	70	2.26	2	8	cyt	cyt	cyt			1	1					1	60S ribosomal protein L10a
Q11004	SPAC1B3.03c|wis2	71	2.25	3	15	cyt; nuc	cyt; nuc	cyt; nuc		1								cyclophilin family peptidyl-prolyl

O74191	SPBC1347.02|fkbp39	72	2.24	1	1	nuc	nuc	nuc	1															FKBP-type peptidyl-prolyl cis-trar
Q9P7X8	SPBP23A10.07|rpa2	73	2.20	7	12	cyt	nuc	cyt			1					1								DNA-directed RNA polymerase I
Q10066	SPAC3H1.07|aru1	74	2.18	3	9	cyt; nuc	cyt; nuc	cyt; nuc		1														arginase Aru1
Q10265	SPAC13G7.02c|ssa1	75	2.16	4	13	cyt	cyt	cyt			1												1	heat shock protein Ssa1 (predicte
Q09130	SPBC17G9.09|tif213	76	2.12	5	14	cyt	cyt	cyt			1												1	translation initiation factor eIF2 g
Q9USZ6	SPBC11G11.03|mrt4	77	2.10	3	4	nuc	nuc	nuc	1															mRNA turnover and ribosome as
O14250	SPAC6G10.04c	78	2.08	1	5	cyt; nuc	cyt; nuc	cyt; nuc		1														20S proteasome component alph
O74135	SPAC1805.05|cki3	79	2.04	1	1	cyt		cyt			1													serine/threonine protein kinase (
Q9Y7N9	SPCC1450.12	80	2.00	1	1	cyt; nuc	cyt; nuc	cyt; nuc		1														conserved fungal protein
Q9P3A7	SPAC1565.08|cdc48	81	2.00	11	89	cyt; nuc	cyt; nuc	cyt; nuc		1														AAA family ATPase Cdc48
Q09912	SPCC830.07c|psi1	82	1.98	1	1	cyt; nuc	cyt; nuc	cyt; nuc		1		1											1	DNAJ domain protein, involved ir
Q10425	SPAC25G10.08	83	1.95	3	6	cyt	cyt	cyt			1												1	translation initiation factor eIF3b
P78761	SPCC613.10|qcr2	84	1.95	4	5	cyt	cyt	cyt			1													ubiquinol-cytochrome-c reductas
Q09717	SPBC1773.07c|sbp1	85	1.93	1	1	cyt	cyt	cyt			1													Ran GTPase binding protein Sbp1
Q96WV5	SPBPJ4664.04	86	1.92	5	6	cyt	cyt	cyt			1													coatomer alpha subunit (predicte
O13907	SPAC22A12.16	87	1.90	3	3	cyt; nuc	cyt; nuc	cyt; nuc		1														ATP-citrate synthase subunit 2 (p
Q09890	SPCC584.16c	88	1.88	1	2	cyt		cyt			1													sequence orphan
O74402	SPBC4F6.17c	89	1.88	1	1	NA	cyt	cyt			1												1	mitochondrial heatshock protein
Q9UUF7	SPBC146.14c|sec26	90	1.87	1	1	cyt	cyt	cyt			1													coatomer beta subunit (predicted
P09322	SPBC8D2.03c|hhf2	91	1.86	5	142	nuc	nuc	nuc	1					1					1			1		histone H4 h4.2
P28189	SPAC6F6.07c|rps13	92	1.86	3	11	cyt	cyt	cyt			1	1											1	40S ribosomal protein S13 (predi
O13960	SPAC1705.03c|ecm33	93	1.86	1	6	cyt	cyt	cyt			1													cell wall protein Ecm33
P41838	SPBC16A3.05c|rae1	94	1.83	2	3	nuc	nuc	nuc	1															RNA export factor, nucleoporin R
O42945	SPBC16H5.10c|prp43	95	1.80	4	8	nuc	nuc	nuc	1															ATP-dependent RNA helicase Prp
Q9USN8	SPCC132.01c	96	1.80	1	1	cyt	cyt	cyt			1													DUF814 family protein
O94616	SPBC1289.07c|rpc4	97	1.79	4	6	cyt; nuc	cyt; nuc	cyt; nuc		1						1								DNA-directed RNA polymerase I a
O59797	SPCC364.06|nap1	98	1.77	1	2	cyt	cyt; nuc	cyt			1			1		1			1		1	1		nucleosome assembly protein Na
O74340	SPBC1A4.07c	99	1.76	3	3	cyt; nuc	nuc	cyt; nuc		1														U3 snoRNP-associated protein Sc
Q9P4W9	SPAC959.07|rps43	100	1.76	1	1	cyt	cyt	cyt			1	1											1	40S ribosomal protein S4
Q10251	SPAC56F8.03	101	1.75	2	2	cyt	cyt	cyt			1	1											1	translation initiation factor IF2 (p
Q09181	SPBC776.09|ste13	102	1.74	1	2	cyt		cyt			1													ATP-dependent RNA helicase Ste
Q09689	SPAC2F7.05c	103	1.73	1	2	cyt	cyt	cyt			1	1											1	translation initiation factor eIF5 (
Q9P782	SPBC1711.08	104	1.71	4	6	cyt	cyt	cyt			1													chaperone activator Aha1 (predic
Q09184	SPAC23H4.09|cdb4	105	1.68	2	3	cyt; nuc	cyt; nuc	cyt; nuc		1														curved DNA-binding protein Cdb
Q09330	SPBC1D7.04|mlo3	106	1.68	1	2	nuc	nuc	nuc	1															RNA binding protein Mlo3
Q10161	SPAC26A3.05|chc1	107	1.67	6	16	cyt	cyt	cyt			1													clathrin heavy chain Chc1 (predic
Q9UUG1	SPAC926.08c	108	1.65	1	3	cyt; nuc	cyt; nuc	cyt; nuc		1														Brix domain protein Rpf2 (predic
O14007	SPAC29A4.04c	109	1.64	4	18	cyt	cyt	cyt			1													pseudouridylate synthase (predic
O60163	SPBC19F5.04	110	1.62	2	3	cyt	cyt	cyt			1													aspartate kinase (predicted)
Q9P7U2	SPAC977.14c	111	1.61	14	43	cyt; nuc	cyt; nuc	cyt; nuc		1														aldo/keto reductase, unknown bi
Q09864	SPAC12G12.04|mcp6	112	1.60	25	156	cyt	cyt	cyt			1													mitochondrial heat shock proteir

O14301	SPAC9G1.05	113	1.58	1	1	cyt; nuc	cyt; nuc	cyt; nuc		1														actin cortical patch component A
Q1K9C2	SPAC10F6.01c|sir1	114	1.58	4	6	cyt	cyt	cyt			1													sulfite reductase Sir1
O94641	SPBC16D10.08c	115	1.57	13	42	cyt; nuc	cyt; nuc	cyt; nuc		1														heat shock protein Hsp104 (pred
O60119	SPBC16G5.05c	116	1.57	1	3	cyt	cyt	cyt			1													MSP domain
O74836	SPCC1183.08c|rpl11	117	1.51	5	48	cyt	cyt	cyt			1	1											1	60S ribosomal protein L10a
O94264	SPBP8B7.15c	118	1.50	1	1	nuc	nuc	nuc	1															ubiquitin-protein ligase E3 RBBP6
Q9UUF2	SPBC17A3.04c|rar1	119	1.48	1	1	cyt	cyt	cyt			1												1	cytoplasmic methionine-tRNA lig
P37382	SPCC1442.10c|rpb3	120	1.47	1	2	cyt; nuc	cyt; nuc	cyt; nuc		1						1					1			RNA polymerase II subunit 3
Q9P6K7	SPAC30C2.04	121	1.46	3	7	NA	cyt	cyt			1												1	cofactor for methionyl-and gluta
P17937	SPAC664.06|rpl73	122	1.46	1	1	nuc	cyt; nuc	nuc	1			1											1	60S ribosomal protein L7
Q09826	SPBC646.13|sds23	123	1.44	2	2	cyt; nuc	cyt; nuc	cyt; nuc		1														inducer of sexual development S
Q02099	SPBC216.05|rad3	124	1.42	1	1	cyt	cyt; nuc	cyt			1				1									ATR checkpoint kinase Rad3
P36620	SPBP35G2.07|ilv1	125	1.41	2	2	cyt	cyt	cyt			1													acetolactate synthase catalytic su
Q9UUA2	SPBC887.14c|pfh1	126	1.38	1	1	cyt; nuc	cyt; nuc	cyt; nuc		1				1	1		1							5' to 3' DNA helicase, involved in
O74864	SPCC18.14c|rpp	127	1.36	6	116	cyt	cyt	cyt			1	1											1	60S acidic ribosomal protein Rpp
O60143	SPBC18H10.12c|rpl71	128	1.35	5	86	nuc	cyt; nuc	nuc	1			1											1	60S ribosomal protein L7
O13672	SPBC29A3.04|rpl8	129	1.34	12	148	NA	cyt	cyt			1	1											1	60S ribosomal protein L7a/L8 (pr
O94620	SPBC1289.11|spf38	130	1.33	2	2	nuc	nuc	nuc	1															splicing factor Spf38
O94681	SPBC776.15c|kgd2	131	1.33	2	6	cyt	cyt	cyt			1													dihydrolipoamide S-succinyltrans
O94524	SPCC1281.07c	132	1.32	1	1	cyt; nuc	cyt; nuc	cyt; nuc		1														glutathione S-transferase Gst3
O74873	SPCC31H12.07|sec23	133	1.31	1	1	NA	cyt	cyt			1													COPII cargo receptor subunit Sec
O42932	SPBC16C6.08c|qcr6	134	1.28	1	1	cyt	cyt	cyt			1													ubiquinol-cytochrome-c reductas
O43036	SPBC3B9.07c|rpa43	135	1.24	2	3	cyt; nuc	cyt; nuc	cyt; nuc		1						1								DNA-directed RNA polymerase I
O74752	SPBC1734.11	136	1.19	1	7	cyt; nuc	cyt; nuc	cyt; nuc		1														DNAJ domain protein Mas5 (pred
Q9USS7	SPBC4.03c	137	1.17	2	2	cyt	cyt	cyt			1													COPII-coated vesicle component
P05732	SPAC3A12.10|rpl21	138	1.17	3	14	cyt	cyt	cyt			1	1											1	60S ribosomal protein L20a
Q9P6I5	SPBC1198.05	139	1.14	1	1	NA	nuc	nuc	1															guanylate kinase (predicted)
P08094	SPBP8B7.06|rpp21	140	1.13	1	2	cyt	cyt	cyt			1	1											1	60S acidic ribosomal protein P2A
Q9P7H0	SPAC1782.10c|nhp2	141	1.11	2	3	nuc	nuc	nuc	1															RNA-binding protein Nhp2 (predi
P40231	SPAC23C11.11|cka1	142	1.11	4	8	cyt; nuc	cyt; nuc	cyt; nuc		1														serine/threonine protein kinase
Q9C0Y6	SPAPB24D3.08c	143	1.07	2	3	cyt; nuc	cyt; nuc	cyt; nuc		1														NADP-dependent oxidoreductase
O75000	SPCC31H12.04c|rpl12	144	1.06	4	36	cyt	cyt	cyt			1	1											1	60S ribosomal protein L12.1/L12
O14362	SPBC30D10.17c|smi1	145	1.04	1	2	cyt		cyt			1													cell wall biosynthesis/ cell cycle r
P31209	SPAC57A7.04c|pabp	146	1.04	3	11	cyt	cyt; nuc	cyt			1												1	mRNA export shuttling protein
O14081	SPACUNK4.16c	147	1.02	1	1	cyt	cyt	cyt			1													alpha,alpha-trehalose-phosphate
O94459	SPAC1687.18c|ssl3	148	0.98	1	1	cyt; nuc	cyt; nuc	cyt; nuc		1				1										cohesin loading factor Ssl3
Q9USZ2	SPBC11G11.07	149	0.98	1	1	cyt; nuc	cyt; nuc	cyt; nuc		1														karyopherin
O74760	SPBC17D11.05|tif32	150	0.97	6	13	cyt	cyt	cyt			1												1	translation initiation factor eIF3a
Q9URY0	SPAC890.04c	151	0.97	4	5	nuc	nuc	nuc	1															ribosome biogenesis protein Ytm
Q10147	SPAC1D4.04|cct2	152	0.95	1	1	cyt; nuc	cyt; nuc	cyt; nuc		1														chaperonin-containing T-complex
O14133	SPAC3C7.07c	153	0.91	1	1	cyt; nuc	cyt; nuc	cyt; nuc		1														arginine-tRNA protein transferase

Q10335	SPBC582.07c|rpn7	154	0.90	1	1	cyt; nuc	cyt; nuc	cyt; nuc		1				1										19S proteasome regulatory subu
Q06975	SPBC20F10.01|gar1	155	0.88	1	2	nuc	nuc	nuc	1															snoRNP pseudouridylase comple
P32390	SPAC630.03|arp3	156	0.86	2	3	cyt	cyt	cyt			1													actin-like protein Arp3
P78827	SPBC56F2.12|ilv5	157	0.85	7	23	cyt	cyt	cyt			1													acetohydroxyacid reductoisomer
O13831	SPAC19A8.15|trp2	158	0.85	1	3	cyt; nuc	cyt; nuc	cyt; nuc		1														tryptophan synthase (predicted)
O59838	SPAC110.04c|pss1	159	0.83	14	64	cyt; nuc	cyt; nuc	cyt; nuc		1														heat shock protein Pss1
O13775	SPAC17A5.15c	160	0.82	2	7	cyt; nuc	cyt; nuc	cyt; nuc		1													1	glutamate-tRNA ligase (predicted
Q9Y7U7	SPCC645.08c|snd1	161	0.81	1	1	cyt	cyt	cyt			1													RNA-binding protein Snd1
Q9UT97	SPAC323.02c|pup2	162	0.81	2	3	cyt; nuc	cyt; nuc	cyt; nuc		1														20S proteasome component alph
P78811	SPCC1322.04	163	0.80	14	60	cyt; nuc	cyt; nuc	cyt; nuc		1														UTP-glucose-1-phosphate uridyly
O94529	SPBC609.05|pob3	164	0.77	2	2	cyt; nuc	cyt; nuc	cyt; nuc		1			1	1	1	1	1	1	1	1	1	1		FACT complex component Pob3
O94374	SPBC1604.08c|imp1	165	0.76	1	2	cyt; nuc	cyt; nuc	cyt; nuc		1														importin alpha
O94267	SPBP8B7.19|spt16	166	0.76	4	6	nuc	nuc	nuc	1					1	1	1	1	1	1		1	1		FACT complex component Spt16
P87140	SPAC57A7.10c|sec21	167	0.76	5	8	cyt	cyt	cyt			1													coatomer gamma subunit Sec21
O94315	SPBC215.11c	168	0.75	2	2	cyt; nuc	cyt; nuc	cyt; nuc		1														aldo/keto reductase, unknown bi
P78854	SPAC1A6.04c|plb1	169	0.73	2	45	cyt; nuc	cyt; nuc	cyt; nuc		1														phospholipase B homolog Plb1
O74538	SPCC74.05|rpl272	170	0.73	1	14	cyt	cyt	cyt			1	1											1	60S ribosomal protein L27
O94515	SPBC646.11|cct6	171	0.70	2	4	cyt; nuc	cyt; nuc	cyt; nuc		1														chaperonin-containing T-comple
Q10289	SPAC4A8.11c|fas2	172	0.69	27	83	cyt	cyt	cyt			1													fatty acid synthase alpha subunit
Q9UQW6	SPBC215.09c|erg1	173	0.66	4	7	cyt	cyt	cyt			1													acetyl-CoA C-acetyltransferase E
P13681	SPBC776.02c|dis2	174	0.65	1	1	cyt; nuc	cyt; nuc	cyt; nuc		1				1										serine/threonine protein phosph
P09627	SPAC1071.10c|pma1	175	0.65	11	61	cyt	cyt	cyt			1													P-type proton ATPase, P3-type P
Q9USX1	SPBC1921.05|ape2	176	0.64	12	21	cyt	cyt	cyt			1													aminopeptidase Ape2
O14459	SPACUNK4.06c|rpb7	177	0.63	2	3	cyt; nuc	cyt; nuc	cyt; nuc		1						1					1			DNA-directed RNA polymerase c
O59677	SPBC29A3.15c|rsm23	178	0.63	1	3	cyt	cyt	cyt			1	1											1	mitochondrial ribosomal protein
Q10205	SPBC17D1.03c	179	0.59	1	1	cyt; nuc	cyt; nuc	cyt; nuc		1														exosome subunit Rrp43 (predicte
P36604	SPAC22A12.15c|bip1	180	0.58	20	82	cyt	cyt; nuc	cyt			1													ER heat shock protein BiP
O13637	SPBC8D2.20c|sec31	181	0.57	3	3	cyt	cyt	cyt			1													COPII-coated vesicle component
Q9USU5	SPBC29A10.08|gas2	182	0.56	2	3	cyt	cyt	cyt			1													1,3-beta-glucanosyltransferase G
O59855	SPCC1739.13|ssa2	183	0.55	11	141	cyt; nuc	cyt; nuc	cyt; nuc		1														heat shock protein Ssa2
Q09740	SPBC12C2.11	184	0.54	2	2	cyt	cyt	cyt			1													glutamine-fructose-6-phosphate
Q9P6L5	SPAC688.11|end4	185	0.52	1	1	cyt	cyt	cyt			1													Huntingtin-interacting protein hc
O42661	SPAC27D7.07c|smd1	186	0.48	2	3	cyt; nuc	cyt; nuc	cyt; nuc		1														Sm snRNP core protein Smd1
O94316	SPBC215.12|cwf1	187	0.48	1	1	cyt; nuc	cyt; nuc	cyt; nuc		1														GTPase Cwf10 (predicted)
P24782	SPBP8B7.16c|dbp2	188	0.46	4	4	cyt; nuc	cyt; nuc	cyt; nuc		1														ATP-dependent RNA helicase Db
Q09878	SPCC584.01c	189	0.45	1	1	cyt	cyt	cyt			1													sulfite reductase NADPH flavopro
O14086	SPAC2F3.03c|rpa49	190	0.44	1	1	nuc	nuc	nuc	1							1								DNA-directed RNA polymerase I
O14164	SPAC4A8.16c|tif33	191	0.41	3	7	cyt	cyt	cyt			1												1	translation initiation factor eIF3c
Q10494	SPAC26F1.07	192	0.38	4	6	cyt; nuc	cyt; nuc	cyt; nuc		1														glucose 1-dehydrogenase (NADP
Q9P7R0	SPAC1834.02|aro1	193	0.37	5	5	cyt	cyt	cyt			1													pentafunctional aromatic polype
Q9US14	SPAC607.04	194	0.36	1	1	cyt; nuc	cyt; nuc	cyt; nuc		1														inositol polyphosphate kinase (p

P78965	SPBC17A3.07|pgr1	195	0.35	2	4	cyt	cyt; nuc	cyt			1													mitochondrial glutathione reduct
O59770	SPCC1795.04c|pre1	196	0.35	3	4	cyt; nuc	cyt; nuc	cyt; nuc		1														20S proteasome component alph
O74407	SPCC1223.07c|drs1	197	0.33	1	1	cyt	cyt	cyt			1												1	cytoplasmic aspartate-tRNA ligas
O94501	SPBC12D12.03|cct1	198	0.31	1	1	cyt; nuc	cyt; nuc	cyt; nuc		1														chaperonin-containing T-complex
P31318	SPAC4G9.09c|arg11	199	0.31	4	5	cyt	cyt	cyt			1													N-acetyl-gamma-glutamyl-phosp
O60124	SPBC16G5.10	200	0.30	1	2	cyt; nuc	cyt; nuc	cyt; nuc		1														exosome subunit Rrp42 (predicte
O94579	SPCC1442.06|pre8	201	0.29	4	11	cyt; nuc	cyt; nuc	cyt; nuc		1														20S proteasome component alph
O42937	SPBC16C6.13c|sec27	202	0.29	2	2	cyt; nuc	cyt; nuc	cyt; nuc		1														coatomer beta' subunit (predicte
Q9UUI5	SPAC22F8.08|sec24	203	0.26	2	3	cyt	cyt	cyt			1													COPII cargo receptor Sec24/Sfb2
Q92399	SPBC14C8.12|rpb8	204	0.25	1	1	cyt; nuc	cyt; nuc	cyt; nuc		1						1					1			DNA-directed RNA polymerase I,
Q9USQ9	SPBC577.10	205	0.23	2	2	cyt; nuc	cyt; nuc	cyt; nuc		1														20S proteasome component beta
O74690	SPAC607.03c|snu13	206	0.22	1	7	nuc	nuc	nuc	1															U3 snoRNP-associated protein Sn
Q9UUM7	SPBC725.09c|hob3	207	0.19	1	2	NA	cyt	cyt			1													BAR adaptor protein Hob3
Q09841	SPAC23D3.07|pup1	208	0.18	1	1	cyt; nuc	cyt; nuc	cyt; nuc		1														20S proteasome component beta
P40235	SPBC3H7.15|hhp1	209	0.18	1	1	cyt; nuc	cyt; nuc	cyt; nuc		1							1							serine/threonine protein kinase H
O14388	SPBC685.07c|rpl271	210	0.17	4	28	cyt; nuc	cyt; nuc	cyt; nuc		1		1											1	60S ribosomal protein L27
P78804	SPCC622.12c|gdh1	211	0.17	8	14	cyt	cyt; nuc	cyt			1													NADP-specific glutamate dehydro
O94634	SPBC1677.03c	212	0.15	2	2	cyt	cyt	cyt			1													mitochondrial threonine ammon
Q09682	SPAC13C5.01c	213	0.15	2	4	cyt; nuc	cyt; nuc	cyt; nuc		1														20S proteasome component alph
O74341	SPBC1A4.08c|cct3	214	0.14	6	7	cyt; nuc	cyt; nuc	cyt; nuc		1														chaperonin-containing T-complex
Q9Y7T8	SPCC63.12c|pup3	215	0.10	1	2	cyt; nuc	cyt; nuc	cyt; nuc		1														20S proteasome component beta
Q9URM2	SPBC2G5.05	216	0.08	15	33	cyt; nuc	cyt; nuc	cyt; nuc		1														transketolase (predicted)
O14021	SPAC29A4.18|prw1	217	0.07	2	3	cyt; nuc	cyt; nuc	cyt; nuc		1			1	1		1		1	1	1				Clr6 histone deacetylase complex
Q09794	SPAC22G7.06c|ura1	218	0.07	6	9	cyt	cyt	cyt			1													carbamoyl-phosphate synthase (
Q9P7B4	SPAC521.03	219	0.06	1	2	cyt	cyt	cyt			1													short chain dehydrogenase (pred
P87317	SPBC31F10.16	220	0.05	1	1	cyt; nuc	cyt; nuc	cyt; nuc		1														ChAPs family protein
Q9UUE1	SPBC17G9.11c|pyr1	221	0.02	14	34	cyt	cyt	cyt			1													pyruvate carboxylase Pyr1
O59827	SPCC965.07c|gst2	222	0.01	2	2	cyt; nuc	cyt; nuc	cyt; nuc		1														glutathione S-transferase Gst2
O94517	SPBC646.16	223	0.01	8	18	cyt; nuc	cyt; nuc	cyt; nuc		1														20S proteasome component alph
O59945	SPBC1778.06c|fim1	224	0.01	6	10	cyt	cyt	cyt			1													fimbrin
Q09883	SPCC188.12|spn6	225	0.01	1	1	cyt	cyt	cyt			1													septin Spn6
Q10329	SPBC106.16|pre6	226	-0.01	2	3	cyt; nuc	cyt; nuc	cyt; nuc		1														20S proteasome component alph
O74374	SPBC32F12.10	227	-0.03	2	3	cyt; nuc	cyt; nuc	cyt; nuc		1														phosphoglucomutase (predicted)
Q09720	SPAC31A2.04c	228	-0.04	1	5	cyt; nuc	cyt; nuc	cyt; nuc		1														20S proteasome component beta
O14460	SPCP31B10.07|eft22	229	-0.05	4	17	cyt	cyt	cyt			1	1											1	translation elongation factor 2 (E
O43063	SPBC4C3.10c|pre3	230	-0.05	3	13	cyt; nuc	cyt; nuc	cyt; nuc		1														20S proteasome component beta
P78921	SPBC337.05c|cct8	231	-0.06	6	12	cyt	cyt	cyt			1													chaperonin-containing T-complex
P30655	SPAC4A8.13c|pts1	232	-0.07	3	13	cyt; nuc	cyt; nuc	cyt; nuc		1														20S proteasome component beta
O14463	SPAC7D4.07c|trx1	233	-0.08	2	5	cyt; nuc	cyt; nuc	cyt; nuc		1					1									cytosolic thioredoxin Trx1
Q9UQY2	SPAC22F8.06|pam1	234	-0.09	3	7	cyt; nuc	cyt; nuc	cyt; nuc		1														20S proteasome component beta
O94242	SPCC736.06	235	-0.09	1	6	cyt	cyt	cyt			1												1	mitochondrial aspartate-tRNA lig

O14295	SPAC9E9.11|plr1	236	-0.10	6	16	cyt; nuc	cyt; nuc	cyt; nuc		1						pyridoxal reductase Plr1
O94337	SPBC1271.04c	237	-0.13	2	4	cyt; nuc	cyt; nuc	cyt; nuc		1					1	eIF-5A-deoxyhypusine synthase
P52808	SPAC9G1.03c|rpl31	238	-0.13	3	17	cyt	cyt	cyt			1	1			1	60S ribosomal protein L30
O43030	SPBC3B9.01	239	-0.14	7	18	cyt; nuc	cyt; nuc	cyt; nuc		1					1	Hsp70 nucleotide exchange facto
O74766	SPBC24C6.04	240	-0.15	9	37	cyt	cyt	cyt			1					delta-1-pyrroline-5-carboxylate d
Q9C102	SPAPB1E7.07|glt1	241	-0.25	2	3	cyt	cyt	cyt			1					glutamate synthase Glt1 (predict
O60105	SPBC14F5.09c|ade8	242	-0.26	8	14	cyt; nuc	cyt; nuc	cyt; nuc		1						adenylosuccinate lyase Ade8
O74891	SPCC576.07|ret3	243	-0.28	3	6	cyt; nuc	cyt; nuc	cyt; nuc		1						coatomer zeta subunit (predictec
O94319	SPBC215.15|sec13	244	-0.29	3	4	cyt; nuc	cyt; nuc	cyt; nuc		1						COPII-coated vesicle component
O94444	SPAC637.10c|rpn1	245	-0.29	1	3	cyt; nuc	cyt; nuc	cyt; nuc		1						19S proteasome regulatory subu
P87153	SPBC25H2.12c|cct7	246	-0.29	4	7	cyt; nuc	cyt; nuc	cyt; nuc		1						chaperonin-containing T-complex
Q10305	SPAC22H10.12c|gdi1	247	-0.29	3	3	cyt	cyt	cyt			1					GDP dissociation inhibitor Gdi1 (p
O43060	SPBC4C3.07	248	-0.33	2	3	cyt; nuc	cyt; nuc	cyt; nuc		1					1	translation initiation factor eIF3f
O74767	SPBC24C6.05|sec28	249	-0.33	2	3	cyt	cyt	cyt			1					coatomer epsilon subunit (predic
O94567	SPBC1773.10c|nrs1	250	-0.34	2	2	cyt	cyt	cyt			1				1	cytoplasmic asparagine-tRNA liga
O74489	SPCC1442.16c|zta1	251	-0.35	7	18	cyt; nuc	cyt; nuc	cyt; nuc		1						NADPH quinone oxidoreductase/
P78780	SPCC1827.06c	252	-0.35	3	4	cyt; nuc	cyt; nuc	cyt; nuc		1						aspartate semialdehyde dehydro
P78774	SPBC14C8.06|arc1	253	-0.36	1	2	cyt	cyt	cyt			1					ARP2/3 actin-organizing complex
P50999	SPBC106.06|cct4	254	-0.37	1	2	cyt; nuc	cyt; nuc	cyt; nuc		1						chaperonin-containing T-complex
Q9P7X1	SPBP23A10.15c|qcr1	255	-0.41	3	8	cyt	cyt	cyt			1					mitochondrial processing peptida
Q09795	SPAC22G7.01c	256	-0.42	2	2	cyt; nuc	cyt; nuc	cyt; nuc		1						iron responsive trasncriptional re
O13877	SPAC1B3.12c|rpb1	257	-0.42	1	1	cyt; nuc	cyt; nuc	cyt; nuc		1			1	1		DNA-directed RNA polymerase I,
O42938	SPBC16H5.02|pfk1	258	-0.43	5	17	cyt	cyt	cyt			1					6-phosphofructokinase (predicte
O14241	SPAC6F6.10c|arc2	259	-0.44	1	1	cyt; nuc	cyt; nuc	cyt; nuc		1						ARP2/3 actin-organizing complex
O74893	SPCC576.09|rps2	260	-0.44	1	7	cyt	cyt	cyt			1	1			1	40S ribosomal protein S20
Q92341	SPAC1F8.03c|str3	261	-0.44	1	1	cyt		cyt			1					siderophore-iron transporter Str3
P78814	SPAC16.02c|srp2	262	-0.46	1	1	NA	nuc	nuc	1							mRNA export factor Srp2
P31406	SPAC343.05|vma1	263	-0.46	4	7	cyt	cyt	cyt			1					V-type ATPase V1 domain, subun
O59804	SPCC550.06c|hsp1	264	-0.47	1	2	cyt	cyt	cyt			1					mitochondrial heat shock proteir
P21696	SPBC215.05|gpd1	265	-0.47	9	25	cyt; nuc	cyt; nuc	cyt; nuc		1						glycerol-3-phosphate dehydroger
Q09191	SPAC23C4.15|rpb5	266	-0.49	2	6	cyt; nuc	cyt; nuc	cyt; nuc		1			1	1		DNA-directed RNA polymerase I,
Q9URX7	SPAC890.07c|rmt1	267	-0.51	1	1	cyt; nuc	cyt; nuc	cyt; nuc		1						type I protein arginine N-methylt
Q9P546	SPAPJ698.02c|rps2	268	-0.51	3	9	cyt	cyt	cyt			1	1			1	40S ribosomal protein S0B
Q9USP8	SPBC902.05c|idh2	269	-0.52	1	2	cyt	cyt	cyt			1					isocitrate dehydrogenase (NAD+)
Q09845	SPAC23D3.04c|gpd2	270	-0.52	1	2	cyt		cyt			1					glycerol-3-phosphate dehydroger
O60085	SPBC14C8.03|fma2	271	-0.54	4	5	cyt	cyt	cyt			1				1	methionine aminopeptidase Fma
Q9P772	SPAP7G5.02c|gua2	272	-0.56	2	2	cyt	cyt	cyt			1					GMP synthase [glutamine-hydrol
Q10318	SPAC17G8.06c	273	-0.57	4	10	cyt	cyt	cyt			1					dihydroxy-acid dehydratase (prec
Q9Y7R8	SPCC306.08c	274	-0.59	6	10	cyt	cyt	cyt			1					malate dehydrogenase
O13848	SPAC19G12.09	275	-0.59	5	15	cyt; nuc	cyt; nuc	cyt; nuc		1						NADH/NADPH dependent indole-
Q9UT48	SPAC821.05	276	-0.60	3	3	cyt; nuc	cyt; nuc	cyt; nuc		1					1	translation initiation factor eIF3h

O13792	SPAC17G6.14c|uap56	277	-0.62	1	3	nuc	nuc	nuc	1									ATP-dependent RNA helicase Ua
O00087	SPAC1002.09c|dld1	278	-0.62	11	14	cyt	cyt	cyt			1							dihydrolipoamide dehydrogenas
P78773	SPCC191.02c	279	-0.63	3	3	cyt; nuc	cyt; nuc	cyt; nuc		1								acetyl-CoA ligase (predicted)
P05219	SPBC26H8.07c|nda3	280	-0.64	4	4	cyt	cyt	cyt			1							tubulin beta Nda3
Q9UUE3	SPBC17G9.06c	281	-0.65	4	5	cyt; nuc	cyt; nuc	cyt; nuc		1								siderophore-iron biosynthesis pr
P78917	SPBC1604.05|pgi1	282	-0.65	12	43	cyt	cyt	cyt			1							glucose-6-phosphate isomerase (
Q9Y6Z9	SPAC8E11.10	283	-0.65	1	1	cyt	cyt	cyt			1							sorbose reductase (predicted)
O74173	SPCC1450.04|tef5	284	-0.66	2	4	cyt	cyt	cyt			1						1	translation elongation factor EF-
O13727	SPAC15A10.07	285	-0.67	1	1	cyt; nuc	cyt; nuc	cyt; nuc		1								sequence orphan
Q10311	SPAC6C3.08	286	-0.69	1	1	cyt; nuc	cyt; nuc	cyt; nuc		1								proteasome regulatory particle,
P04689	SPBC800.05c|atb2	287	-0.70	1	1	cyt; nuc	cyt; nuc	cyt; nuc		1								tubulin alpha 2
O13696	SPAC11G7.03|idh1	288	-0.74	5	7	cyt	cyt	cyt			1							isocitrate dehydrogenase (NAD+)
O60077	SPCC1494.03|arz1	289	-0.76	1	1	cyt; nuc	cyt; nuc	cyt; nuc		1								armadillo repeat containing, Zfs1
O94680	SPBC776.14|plh1	290	-0.78	1	1	cyt	cyt	cyt			1							phospholipid-diacylglycerol acylt
Q9P7F8	SPAC1142.06|get3	291	-0.79	1	1	cyt; nuc	cyt; nuc	cyt; nuc		1								GET complex ATPase subunit Get
Q9UT59	SPAC513.07	292	-0.82	3	3	cyt; nuc	cyt; nuc	cyt; nuc		1								flavonol reductase/cinnamoyl-Co
Q9P7K6	SPBC21C3.19	293	-0.83	1	1	cyt; nuc	cyt; nuc	cyt; nuc		1								SBDS family protein Rtc3 (predict
O74484	SPCC1906.01|mpg1	294	-0.86	1	2	cyt	cyt	cyt			1							mannose-1-phosphate guanyltra
P22774	SPAC664.11|ssc1	295	-0.88	10	46	cyt	cyt	cyt			1							mitochondrial heat shock protei
P78812	SPBC660.16	296	-0.97	10	56	cyt	cyt	cyt			1							phosphogluconate dehydrogena
Q9UUG0	SPAC926.09c|fas1	297	-0.97	10	21	cyt	cyt	cyt			1							fatty acid synthase beta subunit
O42849	SPAC23A1.12c|frs1	298	-0.97	5	5	cyt; nuc	cyt; nuc	cyt; nuc		1							1	phenylalanine-tRNA ligase beta s
Q10137	SPAC3H8.10|spo2	299	-0.99	2	4	cyt	cyt; nuc	cyt			1							sec14 cytosolic factor family Sec
O74413	SPCC1223.14	300	-1.04	1	1	cyt; nuc	cyt; nuc	cyt; nuc		1								chorismate synthase (predicted)
O94273	SPBP8B7.25|cyp4	301	-1.06	1	2	cyt	cyt	cyt			1							cyclophilin family peptidyl-prolyl
O94609	SPBC1604.21c|ptr3	302	-1.06	1	2	cyt; nuc	cyt; nuc	cyt; nuc		1								ubiquitin activating enzyme E1
O13902	SPAC22A12.11|dak1	303	-1.08	5	11	cyt	cyt	cyt			1							dihydroxyacetone kinase Dak1
O94675	SPBC776.07	304	-1.08	1	2	cyt	cyt	cyt			1							mitochondrial Mam33 family pro
P40374	SPBC21H7.07c|his5	305	-1.11	2	4	cyt; nuc	cyt; nuc	cyt; nuc		1								imidazoleglycerol-phosphate de
Q9UUA1	SPCC23B6.01c	306	-1.12	1	2	cyt; nuc	cyt; nuc	cyt; nuc		1								oxysterol binding protein (predic
O14254	SPAC6G10.08|idp1	307	-1.12	1	2	cyt	cyt	cyt			1							isocitrate dehydrogenase Idp1
P87147	SPBC25H2.05|egd2	308	-1.17	2	6	cyt	cyt	cyt			1	1						nascent polypeptide-associated
Q10280	SPCC1902.01|gaf1	309	-1.17	1	2	cyt	cyt; nuc	cyt			1		1	1	1	1		transcription factor Gaf1
Q92352	SPAC6G9.07c|arc4	310	-1.20	1	5	cyt; nuc	cyt; nuc	cyt; nuc		1								ARP2/3 actin-organizing complex
P17610	SPAC18G6.03|ypt3	311	-1.21	1	1	cyt; nuc	cyt; nuc	cyt; nuc		1								GTPase Ypt3
P18253	SPBC28F2.03|ppi1	312	-1.23	2	9	cyt; nuc	cyt; nuc	cyt; nuc		1								cyclophilin family peptidyl-prolyl
Q10490	SPAC26F1.13c|lrs1	313	-1.25	2	2	cyt	cyt	cyt			1						1	cytoplasmic leucine-tRNA ligase
O14293	SPAC9E9.09c	314	-1.26	12	38	cyt; nuc	cyt; nuc	cyt; nuc		1								aldehyde dehydrogenase (predic
P55306	SPCC757.07c|ctt1	315	-1.28	4	6	cyt; nuc	cyt; nuc	cyt; nuc		1								catalase
O13864	SPAC1B1.03c|kap95	316	-1.30	1	2	nuc	nuc	nuc	1									karyopherin Kap95
Q9UUC2	SPBC365.01	317	-1.31	2	2	cyt; nuc	cyt; nuc	cyt; nuc		1								sec14 cytosolic factor family

P87048	SPBP19A11.03c|mts4	318	-1.37	2	3	cyt; nuc	cyt; nuc	cyt; nuc	1														19S proteasome regulatory subu
Q9Y823	SPBC1105.02c|lys4	319	-1.37	1	2	cyt	cyt; nuc	cyt		1													homocitrate synthase (predicted
P40370	SPBC1815.01|eno11	320	-1.41	18	424	cyt; nuc	cyt; nuc	cyt; nuc	1														enolase
O94284	SPBC2G5.06c|hmt2	321	-1.41	1	1	cyt	cyt	cyt		1													sulfide-quinone oxidoreductase
Q9P7W3	SPBC1703.07	322	-1.42	11	23	cyt; nuc	cyt; nuc	cyt; nuc	1														ATP citrate synthase subunit 1 (p
P36580	SPBC19C2.07|fba1	323	-1.42	11	145	cyt; nuc	cyt; nuc	cyt; nuc	1														fructose-bisphosphate aldolase F
Q09179	SPAC23H4.06|gln1	324	-1.43	2	4	cyt; nuc	cyt; nuc	cyt; nuc	1														glutamate-ammonia ligase Gln1
Q9P7L5	SPBC21C3.08c|car2	325	-1.44	5	12	cyt; nuc	cyt; nuc	cyt; nuc	1														ornithine transaminase Car2
O60128	SPBC16G5.14c|rps3	326	-1.45	2	10	cyt	cyt	cyt		1	1											1	40S ribosomal protein S3
Q9UQX0	SPAC1486.01	327	-1.46	1	2	cyt	cyt	cyt		1													manganese superoxide dismutas
P50514	SPBC1539.03c	328	-1.49	1	2	cyt	cyt	cyt		1													argininosuccinate lyase
P40387	SPAC328.03|tps1	329	-1.50	1	1	cyt	cyt	cyt		1													alpha,alpha-trehalose-phosphate
Q9P7T6	SPAC694.04c	330	-1.51	1	1	cyt; nuc	cyt; nuc	cyt; nuc	1														conserved eukaryotic protein
O94552	SPCC18.18c|fum1	331	-1.52	1	2	cyt	cyt	cyt		1													fumarate hydratase
Q10057	SPAC1F5.02	332	-1.53	9	23	cyt	cyt	cyt		1													protein disulfide isomerase (prec
O74790	SPBC26H8.06|grx4	333	-1.54	1	1	cyt; nuc	cyt; nuc	cyt; nuc	1														glutaredoxin Grx4
P35551	SPBC2D10.10c|fib1	334	-1.55	3	8	cyt; nuc	nuc	cyt; nuc	1														fibrillarin
P30821	SPAC3C7.14c|obr1	335	-1.59	3	13	cyt; nuc	cyt; nuc	cyt; nuc	1			1			1				1				ubiquitinated histone-like protein
O94354	SPBC428.05c|arg12	336	-1.60	1	2	cyt	cyt	cyt		1													argininosuccinate synthase (pred
P78937	SPBC27.08c|sua1	337	-1.61	9	20	cyt	cyt	cyt		1													sulfate adenylyltransferase
P49740	SPAC806.07|ndk1	338	-1.64	1	2	cyt; nuc	cyt; nuc	cyt; nuc	1														nucleoside diphosphate kinase N
P40373	SPAC25G10.05c|his1	339	-1.65	3	8	cyt	cyt	cyt		1													ATP phosphoribosyltransferase
O13966	SPAC24C9.06c	340	-1.65	1	4	cyt	cyt	cyt		1													aconitate hydratase
P10989	SPBC32H8.12c|act1	341	-1.68	2	8	cyt	cyt; nuc	cyt		1			1				1	1					actin Act1
O59711	SPBC3B8.03	342	-1.68	1	1	cyt	cyt	cyt		1													saccharopine dehydrogenase (pr
O42909	SPBC16A3.02c	343	-1.69	2	4	cyt	cyt	cyt		1													mitochondrial peptidase (predict
O60101	SPBC14F5.04c|pgk1	344	-1.69	24	164	cyt; nuc	cyt; nuc	cyt; nuc	1														phosphoglycerate kinase Pgk1 (p
O60125	SPBC16G5.11c|bag11	345	-1.72	1	1	cyt; nuc	nuc	cyt; nuc	1														BAG family molecular chaperone
Q9UTJ7	SPAC1556.02c|sdh1	346	-1.72	4	7	cyt	cyt	cyt		1													succinate dehydrogenase Sdh1
Q09741	SPBC12C2.07c	347	-1.73	5	7	cyt; nuc	cyt; nuc	cyt; nuc	1														spermidine synthase (predicted)
P42656	SPAC8E11.02c|rad24	348	-1.74	7	17	cyt; nuc	cyt	cyt; nuc	1			1			1				1	1			14-3-3 protein Rad24
O13849	SPAC19G12.10c|cpy1	349	-1.76	2	6	cyt	cyt	cyt		1													vacuolar carboxypeptidase Y
P87168	SPBC3D6.03c|trzl	350	-1.76	1	1	cyt	cyt	cyt		1													3'-tRNA processing endonuclease
Q03392	SPBC16D10.09|pcn1	351	-1.78	2	4	cyt; nuc	cyt; nuc	cyt; nuc	1					1		1							PCNA
O74976	SPCC1827.03c	352	-1.79	2	4	cyt	cyt	cyt		1													acetyl-CoA ligase (predicted)
O74548	SPCC777.09c|arg1	353	-1.81	3	8	cyt; nuc	cyt; nuc	cyt; nuc	1														acetylornithine aminotransferase
O14122	SPAC3A11.08|pcu4	354	-1.81	1	1	cyt; nuc	cyt; nuc	cyt; nuc	1			1	1	1	1	1	1	1	1		1		cullin 4
O74827	SPBC337.16|cho1	355	-1.83	1	1	cyt	cyt	cyt		1													phosphatidyl-N-methylethanolar
O94331	SPBC725.15|ura5	356	-1.87	1	1	cyt; nuc	cyt; nuc	cyt; nuc	1														orotate phosphoribosyltransfera
Q9UTR1	SPAC1071.02	357	-1.90	1	1	cyt; nuc	cyt; nuc	cyt; nuc	1						1	1				1			TFIIH regulator (predicted)
O14055	SPCC1672.05c|yrs1	358	-1.91	1	1	cyt	cyt	cyt		1												1	cytoplasmic tyrosine-tRNA ligase

O14255	SPAC6G10.09	359	-1.92	1	1	cyt	cyt	cyt			1						alpha glucosidase I Gls1 (predicte
P25457	SPAC3H5.07|rpl72	360	-1.93	2	2	cyt; nuc	cyt; nuc	cyt; nuc		1		1				1	60S ribosomal protein L7
O13651	SPBC8D2.06|irs1	361	-1.94	1	1	cyt	cyt	cyt			1					1	cytoplasmic isoleucine-tRNA ligas
Q10104	SPAC18G6.04c|shm2	362	-2.01	1	3	cyt	cyt	cyt			1						serine hydroxymethyltransferase
Q10489	SPAC26F1.03|pda1	363	-2.05	1	1	cyt	cyt	cyt			1						pyruvate dehydrogenase e1 com
Q9P777	SPBC1711.13|his2	364	-2.06	1	1	cyt; nuc	cyt; nuc	cyt; nuc		1							histidinol dehydrogenase His2 (p
O14027	SPAC29B12.04|snz1	365	-2.09	4	11	cyt	cyt	cyt			1						pyridoxine biosynthesis protein
Q10208	SPAC4H3.10c|pyk1	366	-2.09	11	68	cyt	cyt	cyt			1						pyruvate kinase (predicted)
Q9P7D4	SPBP4H10.15	367	-2.10	1	1	cyt	cyt	cyt			1	1				1	aconitate hydratase/mitochondri
O94505	SPCC1753.04|tol1	368	-2.11	1	2	cyt; nuc	cyt; nuc	cyt; nuc		1							3'(2'),5'-bisphosphate nucleotida
Q09781	SPAC13G6.02c|rps11	369	-2.12	1	1	cyt	cyt	cyt			1	1				1	40S ribosomal protein S3a
Q09694	SPAC227.18|lys3	370	-2.14	2	6	cyt		cyt			1						saccharopine dehydrogenase Lys
P56286	SPAC3G9.09c|tif211	371	-2.16	4	15	cyt; nuc	cyt; nuc	cyt; nuc		1						1	translation initiation factor eIF2 a
P78929	SPAC20G4.06c|adf1	372	-2.17	2	6	cyt	cyt; nuc	cyt			1						actin depolymerizing factor, cofil
Q10179	SPAC3F10.03|grs1	373	-2.18	1	1	cyt	cyt	cyt			1					1	mitochondrial and cytoplasmic gl
Q9UST4	SPBC336.10c|tif512	374	-2.19	1	1	cyt; nuc	cyt; nuc	cyt; nuc		1		1				1	translation elongation factor eIF5
Q92375	SPBC3F6.03|trr1	375	-2.21	3	6	cyt; nuc	cyt; nuc	cyt; nuc		1							thioredoxin reductase Trr1
O60100	SPBC14F5.03c|kap123	376	-2.23	1	1	cyt; nuc	cyt; nuc	cyt; nuc		1							karyopherin Kap123
O13963	SPAC24C9.03|mvd1	377	-2.25	2	2	cyt; nuc	cyt; nuc	cyt; nuc		1							diphosphomevalonate decarboxy
O42926	SPBC16C6.02c|vps132	378	-2.25	1	3	NA	cyt	cyt			1						chorein homolog
O59736	SPBC3E7.16c|leu3	379	-2.27	2	2	cyt	cyt	cyt			1						2-isopropylmalate synthase (pred
P40903	SPAC4A8.04|isp6	380	-2.28	1	2	cyt	cyt	cyt			1						vacuolar serine protease Isp6
O14306	SPAC9G1.10c	381	-2.29	1	2	cyt	cyt	cyt			1						inositol polyphosphate phosphat
O13682	SPAC11E3.01c|swr1	382	-2.29	1	1	nuc	nuc	nuc	1				1	1	1		SNF2 family helicase Swr1
O60110	SPBC15C4.01c|oca3	383	-2.29	1	1	cyt; nuc	cyt; nuc	cyt; nuc		1							TPR repeat protein Oca3/ ER mer
Q9P7D6	SPBP4H10.12	384	-2.29	1	1	cyt; nuc	cyt; nuc	cyt; nuc		1							conserved protein
Q09748	SPBC12C2.08|dnm1	385	-2.31	2	3	cyt	cyt	cyt			1						dynamin Dnm1
O60052	SPAPB1A10.04c|cwp1	386	-2.33	1	1	nuc	nuc	nuc	1								geranylgeranyltransferase I alpha
O42867	SPCC1322.11|rpl232	387	-2.37	2	6	cyt; nuc	cyt; nuc	cyt; nuc		1		1				1	60S ribosomal protein L23
Q9P6I2	SPBC1198.08	388	-2.40	1	1	cyt; nuc	cyt; nuc	cyt; nuc		1							dipeptidase Dug1 (predicted)
O13642	SPBC8D2.15|lip5	389	-2.41	1	1	cyt	cyt	cyt			1						mitochondrial lipoic acid synthet
O13899	SPAC22A12.08c	390	-2.41	2	3	cyt	cyt	cyt			1						cardiolipin synthase/ hydrolase fu
P50522	SPCC794.09c|ef1a-a	391	-2.44	1	3	cyt	cyt	cyt			1					1	translation elongation factor EF-1
Q09751	SPBC12C2.12c|glo1	392	-2.44	1	1	cyt; nuc	cyt; nuc	cyt; nuc		1							glyoxalase I
P4H10.	SPBP4H10.12	393	-2.45	1	1	cyt; nuc	cyt; nuc	cyt; nuc		1							conserved protein
O74830	SPCC1183.02	394	-2.46	1	1	cyt	cyt	cyt			1					1	glutathione S-transferase (predic
P50521	SPAC4F8.07c|hxk2	395	-2.48	1	1	cyt; nuc	cyt; nuc	cyt; nuc		1							hexokinase 2
P47943	SPAC1006.07	396	-2.49	5	10	cyt	cyt	cyt			1					1	translation initiation factor eIF4A
Q09733	SPAC31A2.16|gef2	397	-2.50	1	1	cyt; nuc	cyt	cyt; nuc		1							RhoGEF Gef2
Q9C1X2	SPBC713.03	398	-2.50	1	2	cyt	cyt	cyt			1						mitochondrial D-lactate dehydro
O14370	SPBC428.02c|eca39	399	-2.55	2	4	cyt; nuc	cyt; nuc	cyt; nuc		1							branched chain amino acid amin

P36579	SPBC4F6.18c|arf1	400	-2.55	4	19	cyt	cyt	cyt			1								ADP-ribosylation factor, Arf famil
O94313	SPBC215.08c|arg4	401	-2.55	4	6	cyt	cyt	cyt			1								arginine specific carbamoyl-phos
P18869	SPBC1A4.02c|leu1	402	-2.56	4	10	cyt	cyt	cyt			1								3-isopropylmalate dehydrogenas
O74781	SPBC25B2.09c|mrs1	403	-2.57	5	7	cyt	cyt	cyt			1							1	mitochondrial and cytoplasmic ar
Q02787	SPAC144.03|ade2	404	-2.58	2	6	cyt; nuc	cyt; nuc	cyt; nuc		1									adenylosuccinate synthetase Ade
O60198	SPBC14F5.05c|sam1	405	-2.58	4	7	cyt; nuc	cyt; nuc	cyt; nuc		1									S-adenosylmethionine synthetase
O14351	SPBC30D10.05c	406	-2.58	2	3	cyt; nuc	cyt; nuc	cyt; nuc		1									sepiapterin reductase (predicted
P19117	SPAC23C11.05	407	-2.58	6	18	cyt; nuc	cyt; nuc	cyt; nuc		1									inorganic pyrophosphatase (pred
Q10306	SPAC6C3.04|cit1	408	-2.61	2	3	cyt	cyt	cyt			1								citrate synthase Cit1
P35679	SPBP8B7.03c|rpl42	409	-2.62	1	1	cyt	cyt	cyt			1	1						1	60S ribosomal protein L2
P49731	SPBC211.04c|mcm6	410	-2.65	1	1	cyt	cyt; nuc	cyt			1				1				MCM complex subunit Mcm6
O74960	SPCC736.15	411	-2.70	2	3	cyt		cyt			1								protein kinase inhibitor associate
O13326	SPBC428.11	412	-2.70	1	1	cyt; nuc	cyt; nuc	cyt; nuc		1									homocysteine synthase Met17
O14104	SPAC31G5.04	413	-2.72	2	3	cyt	cyt	cyt			1								homoisocitrate dehydrogenase
O74482	SPCC1840.09	414	-2.74	1	1	cyt	cyt	cyt			1								NAD dependent epimerase/dehy
Q9UUB4	SPBC409.10|ade7	415	-2.75	1	1	cyt; nuc	cyt; nuc	cyt; nuc		1									phosphoribosylamidoimidazolesu
P40375	SPCC794.12c|mae2	416	-2.76	6	13	cyt	cyt	cyt			1								malic enzyme, malate dehydroge
P78825	SPCC338.14	417	-2.76	4	4	cyt; nuc	cyt; nuc	cyt; nuc		1									adenosine kinase (predicted)
O59858	SPBC32F12.03c|gpx1	418	-2.78	2	2	cyt; nuc	cyt; nuc	cyt; nuc		1									glutathione peroxidase Gpx1
P78753	SPBC119.10|asn1	419	-2.79	1	2	cyt; nuc	cyt; nuc	cyt; nuc		1									asparagine synthetase
P07669	SPCC24B10.21|tpi1	420	-2.82	5	24	cyt; nuc	cyt; nuc	cyt; nuc		1									triosephosphate isomerase
Q9HGP5	SPAC631.01c|acp2	421	-2.83	2	5	cyt; nuc	cyt; nuc	cyt; nuc		1									F-actin capping protein beta subu
O13834	SPAC19D5.04|ptr1	422	-2.85	1	1	nuc	nuc	nuc	1										HECT-type ubiquitin ligase E3 Ptr
P22068	SPAC222.12c|atp2	423	-2.85	5	8	cyt	cyt	cyt			1								F1-ATPase beta subunit Atp2
Q00472	SPBC15D4.15|pho2	424	-2.86	2	3	cyt; nuc	cyt; nuc	cyt; nuc		1									4-nitrophenylphosphatase
P33075	SPAC4G9.03|adk1	425	-2.86	2	2	cyt; nuc	cyt; nuc	cyt; nuc		1									adenylate kinase Adk1
O94438	SPAC22H12.04c|rps1	426	-2.86	1	2	cyt	cyt	cyt			1	1						1	40S ribosomal protein S3a (predi
O60064	SPBC13G1.02	427	-2.88	1	1	cyt; nuc	cyt; nuc	cyt; nuc		1									mannose-1-phosphate guanyltra
O74928	SPCPB16A4.03c|ade1	428	-2.88	7	14	cyt	cyt	cyt			1								IMPcyclohydrolase/phosphoribo
P36623	SPAC26F1.06|gpm1	429	-2.89	10	39	cyt; nuc	cyt; nuc	cyt; nuc		1									monomeric 2,3-bisphosphoglyce
Q9US42	SPAC1002.18|urg3	430	-2.93	1	1	cyt; nuc	cyt; nuc	cyt; nuc		1									DUF1688 family protein
O74887	SPCC576.03c|tpx1	431	-2.94	4	35	cyt; nuc	cyt; nuc	cyt; nuc		1								1	thioredoxin peroxidase Tpx1
P30776	SPCC338.17c|rad21	432	-2.98	1	1	cyt; nuc	nuc	cyt; nuc		1				1			1		mitotic cohesin complex, non-SM
Q8WZJ7	SPBC839.16	433	-2.99	6	11	cyt	cyt	cyt			1								C1-5,6,7,8-tetrahydrofolate (THF
P21535	SPMIT.07|meu15	434	-3.01	1	1	cyt	cyt	cyt			1								sequence orphan
P79071	SPCC622.18|rpl6	435	-3.03	1	3	cyt; nuc	cyt; nuc	cyt; nuc		1		1						1	60S ribosomal protein L6
O43049	SPBC3F6.01c	436	-3.05	1	1	NA	nuc	nuc	1										serine/threonine protein phosph
O60155	SPBC19C7.06|prs1	437	-3.05	1	1	cyt	cyt	cyt			1							1	cytoplasmic proline-tRNA ligase F
Q10232	SPAC4G9.16c|rpl91	438	-3.06	1	3	cyt; nuc	cyt; nuc	cyt; nuc		1		1						1	60S ribosomal protein L9
Q09736	SPAC13A11.02c|erg11	439	-3.08	1	1	cyt	cyt	cyt			1								sterol 14-demethylase
P87144	SPBC25H2.02|trs1	440	-3.09	1	1	cyt	cyt	cyt			1							1	cytoplasmic threonine-tRNA ligas

Q09171	SPBC30D10.13c|pdb1	441	-3.12	1	2	cyt	cyt	cyt			1													pyruvate dehydrogenase e1 com
O59722	SPBC3E7.01|fab1	442	-3.12	1	5	cyt	cyt	cyt			1													1-phosphatidylinositol-3-phospha
O14313	SPCC330.06c	443	-3.17	2	4	cyt; nuc	cyt; nuc	cyt; nuc		1														thioredoxin peroxidase
Q09700	SPAC2F7.09c	444	-3.17	1	1	cyt	cyt	cyt			1													mitochondrial GTPase related pro
O75005	SPBC1709.02c|vrs1	445	-3.19	4	5	cyt	cyt	cyt			1												1	cytoplasmic valine-tRNA ligase Vr
Q42598	SPAC9E9.06c	446	-3.19	1	2	cyt; nuc	cyt; nuc	cyt; nuc		1														threonine synthase (predicted)
P05752	SPAC13G6.07c|rps61	447	-3.19	1	2	cyt	cyt	cyt			1	1											1	40S ribosomal protein S6
O42634	SPAC8E11.03c|dmc1	448	-3.20	1	1	cyt; nuc	cyt; nuc	cyt; nuc		1							1							RecA family ATPase Dmc1
Q9Y7T1	SPCC63.05	449	-3.20	1	1	cyt; nuc	cyt; nuc	cyt; nuc		1														TAP42 family protein (predicted)
Q10284	SPBC1709.05|sks2	450	-3.21	5	5	cyt	cyt	cyt			1												1	heat shock protein, ribosome ass
O74728	SPBC1709.03	451	-3.27	1	1	cyt	cyt	cyt			1													conserved fungal protein
O74455	SPCC16C4.10	452	-3.43	4	4	cyt; nuc	cyt; nuc	cyt; nuc		1														6-phosphogluconolactonase (pre
O74335	SPBC17F3.01c|rga5	453	-3.43	1	1	cyt	cyt	cyt			1													Rho-type GTPase activating prote
O94489	SPCC417.08|tef3	454	-3.44	13	36	cyt	cyt	cyt			1	1											1	translation elongation factor eEF
P78958	SPBC32F12.11|tdh1	455	-3.49	3	18	cyt	cyt	cyt			1													glyceraldehyde-3-phosphate deh
O14018	SPAC29A4.15	456	-3.51	1	1	cyt	cyt	cyt			1												1	cytoplasmic serine-tRNA ligase Sr
Q9USL6	SPCC18B5.05c	457	-3.55	1	1	cyt; nuc	cyt; nuc	cyt; nuc		1														phosphomethylpyrimidine kinase
O42700	SPCC1020.06c|tal1	458	-3.57	3	3	cyt; nuc	cyt; nuc	cyt; nuc		1														transaldolase (predicted)
Q9C0Z7	SPAPB1E7.12|rps62	459	-3.59	1	2	cyt	cyt	cyt			1	1											1	40S ribosomal protein S6
Q9UTJ2	SPAC1556.07|pmm1	460	-3.63	1	1	cyt; nuc	cyt; nuc	cyt; nuc		1														phosphomannomutase Pmm1
Q10344	SPAC1F12.02c	461	-3.66	1	3	cyt; nuc	cyt; nuc	cyt; nuc		1													1	translationally controlled tumor p
O74892	SPCC576.08c|rps2	462	-3.78	3	6	cyt	cyt	cyt			1	1											1	40S ribosomal protein S2
P79057	SPBP4H10.13|rps232	463	-3.81	1	3	nuc	cyt; nuc	nuc	1			1											1	40S ribosomal protein S23 (predi
P78790	SPAC27D7.06	464	-3.81	1	1	cyt	cyt	cyt			1													electron transfer flavoprotein alp
Q9HDY4	SPAPB1A10.06c	465	-3.90	1	1	nuc	nuc	nuc	1															ATP-dependent RNA helicase Dhr
O13811	SPAC17H9.14c	466	-3.90	1	1	NA	cyt	cyt			1													protein disulfide isomerase
O14110	SPAC31G5.14|gcv1	467	-3.91	1	1	cyt	cyt	cyt			1													glycine decarboxylase T subunit
Q09920	SPAC1F7.08|fio1	468	-3.98	1	1	cyt	cyt	cyt			1													iron transport multicopper oxida
Q09889	SPCC584.15c	469	-4.01	1	1	cyt	cyt	cyt			1													arrestin/PY protein 2
Q9UTA6	SPAC25B8.12c	470	-4.05	3	5	cyt; nuc	cyt; nuc	cyt; nuc		1														nucleotide-sugar phosphatase (p
O94754	SPCC1259.01c|rps182	471	-4.15	2	6	cyt; nuc	cyt; nuc	cyt; nuc		1		1											1	40S ribosomal protein S18 (predi
Q9USG6	SPCP31B10.08c|rpl35	472	-4.15	1	1	NA	cyt	cyt			1	1											1	60S ribosomal protein L35a
Q9US12	SPAC607.06c	473	-4.20	1	1	cyt; nuc	cyt; nuc	cyt; nuc		1														metallopeptidase
O14075	SPACUNK4.10	474	-4.28	1	1	cyt; nuc	cyt; nuc	cyt; nuc		1														glyoxylate reductase (predicted)
O94639	SPBC16D10.06|zrt1	475	-4.30	1	1	cyt	cyt	cyt			1		1			1				1	1			ZIP zinc transporter Zrt1
O13639	SPBC8D2.18c	476	-4.40	8	24	cyt	cyt	cyt			1													adenosylhomocysteinase (predic
Q10101	SPAC18G6.14c|rps7	477	-4.41	3	7	cyt; nuc	cyt; nuc	cyt; nuc		1		1											1	40S ribosomal protein S7 (predict
O13660	SPBC27B12.09c	478	-4.43	1	1	cyt	cyt	cyt			1													mitochondrial FAD transporter (p
Q09669	SPAC5H10.06c|adh4	479	-4.44	2	3	cyt	cyt	cyt			1													alcohol dehydrogenase Adh4
Q10429	SPCC188.03|cnd3	480	-4.46	1	1	cyt; nuc	cyt; nuc	cyt; nuc		1				1										condensin complex non-SMC sub
Q09785	SPAC13G6.06c|gcv2	481	-4.53	3	3	cyt	cyt	cyt			1													glycine cleavage complex subunit

Q09690	SPAC2F7.03c|pom1	482	-4.57	1	2	cyt	cyt	cyt			1													DYRK family protein kinase Pom1
Q9UT19	SPAC9.09|met26	483	-4.57	13	59	cyt; nuc	cyt; nuc	cyt; nuc		1														homocysteine methyltransferase
P87112	SPAC20G8.06|not1	484	-4.66	1	2	cyt; nuc	cyt; nuc	cyt; nuc		1						1								CCR4-Not complex subunit Not1
Q8NIL3	SPBPB21E7.07|aes1	485	-4.70	1	1	cyt; nuc	cyt; nuc	cyt; nuc		1			1			1				1				enhancer of RNA-mediated gene
O43003	SPBC2G2.04c|mmf1	486	-4.71	1	2	cyt; nuc	cyt; nuc	cyt; nuc		1														YjgF family protein Mmf1
Q9P7I8	SPCC24B10.18	487	-4.77	1	2	nuc	nuc	nuc	1															human Leydig cell tumor 10 kDa
O14230	SPAC6F12.13c|fps1	488	-4.84	1	1	cyt; nuc	cyt; nuc	cyt; nuc		1														geranyltranstransferase Fps1
O94461	SPAC1687.21	489	-4.87	1	1	cyt; nuc	cyt; nuc	cyt; nuc		1														phosphoglycerate mutase family
O94356	SPBC428.07|meu6	490	-4.92	1	3	cyt; nuc	nuc	cyt; nuc		1														meiotic chromosome segregation
Q10157	SPBC17G9.10|rpl112	491	-5.14	2	4	cyt	cyt	cyt			1	1											1	60S ribosomal protein L11
P79013	SPAC31G5.03|rps111	492	-5.27	1	1	cyt; nuc	cyt; nuc	cyt; nuc		1		1											1	40S ribosomal protein S11 (predi
Q9US51	SPAC1002.08c|mtf1	493	-5.28	1	1	cyt	cyt	cyt			1					1								mitochondrial RNA polymerase s
P78946	SPBC29B5.03c|rpl26	494	-5.29	1	1	cyt; nuc	cyt; nuc	cyt; nuc		1		1											1	60S ribosomal protein L26 (predi
P87115	SPAC20G8.09c	495	-5.30	1	1	nuc	nuc	nuc	1															ribosome biogenesis ATPase (pre
O14121	SPAC3A11.07	496	-5.31	1	1	cyt	cyt	cyt			1													NADH dehydrogenase
Q10327	SPAC32A11.02c	497	-5.35	1	1	cyt		cyt			1													conserved fungal protein
O13984	SPAC26H5.02c	498	-5.44	1	1	NA	nuc	nuc	1						1		1							DNA replication ATPase (predicte
P24487	SPAC14C4.14|atp1	499	-5.50	1	1	NA	cyt	cyt			1													F1-ATPase alpha subunit
O60055	SPBC56F2.04|utp2	500	-5.75	1	3	nuc	nuc	nuc	1															U3 snoRNP protein Utp20
O42968	SPBC1E8.03c	501	-5.81	1	2	cyt	cyt	cyt			1													conserved fungal protein
Q8TFG6	SPAPB18E9.02c|ppk1ٍ	502	-5.87	1	2	cyt	cyt; nuc	cyt			1		1			1				1				serine/threonine protein kinase I
O74808	SPBC2D10.17|clr1	503	-6.13	1	1	nuc	nuc	nuc	1				1	1		1		1	1	1	1			cryptic loci regulator Clr1
Q9USI4	SPCC70.06	504	-6.41	1	1	cyt; nuc	cyt; nuc	cyt; nuc		1														nuclear export factor (predicted)
Q09190	SPAC1556.04c|cdd1	505	-6.79	1	1	cyt; nuc	cyt; nuc	cyt; nuc		1														cytidine deaminase Ccd1
O74764	SPBC24C6.02	506	-7.69	1	1	nuc	nuc	nuc	1															ATP-dependent RNA helicase Spb
P87243	SPCC4G3.18	507	-9.44	1	1	nuc	nuc	nuc	1															ribosome biogenesis protein Rix1

Note: a, rank is based on the ratio between light protein level (ChAP-extract) and heavy protein level (WCE); b, ratio in log2 scale; c, number of unique peptides identified; d, number of spectra identified; e,localization based on the study by Matsuyama et al (2006); f, localization based on the gene ontolog (CC); g, localization based on Matsuyama's study and GO terms; h, nuclear localization; i, nuclear and cytoplasmic localization; j, cytoplasmic localization; k, ribosomal proteins; l, regulation of transcription; m, chromosome organization; n, DNA replication; o, transcription; p, DNA repair; q, chromatin modification; r, chromatin organization; s, regulation of DNA-dependent transcription; t, type II transcription; u, chromatin assembly; v, translation; w, gene product based on the genome annotation at www.genedb.org.

Supplemenal Table S3. Absolute abundance of proteins in CHRAP-prep

Uniprot ID	Sysname|symbol	Rank[a]	Abundance (log2)[b]	# of unique peptide[c]	# of spectrum[d]	Localization (Matsuyama et al 2006)[e]	Localization (GO terms)[f]	Localization (combined)[g]	nuc[h]	cyt; nuc[i]	cyt[j]	ribo[k]	GO:0045449[l]	GO:0051276[m]	GO:0006350[n]	GO:0006325[o]	GO:0006366[p]	GO:0006412[q]	product[r]
O74836	SPCC1183.08c|rpl101	1	20.51	6	156	cyt	cyt	cyt			1	1						1	60S ribosomal protein L10a
O74538	SPCC74.05|rpl2702	2	18.61	4	37	cyt	cyt	cyt			1	1						1	60S ribosomal protein L27
O75000	SPCC31H12.04c|rpl1202	3	18.32	4	235	cyt	cyt	cyt			1	1						1	60S ribosomal protein L12.1/L12A
O74864	SPCC18.14c|rpp0	4	18.15	9	201	cyt	cyt	cyt			1	1						1	60S acidic ribosomal protein Rpp0
O14007	SPAC29A4.04c	5	18.03	8	33	cyt	cyt	cyt			1								pseudouridylate synthase (predict
O14469	SPAC5D6.01|rps2202	6	17.99	4	19		cyt	cyt			1	1						1	40S ribosomal protein S15a
O94238	SPAC1805.13|rpl14	7	17.62	5	56	cyt; nuc	cyt; nuc	cyt; nuc		1		1						1	60S ribosomal protein L14 (predict
Q09191	SPAC23C4.15|rpb5	8	16.50	2	6	cyt; nuc	cyt; nuc	cyt; nuc		1					1		1		DNA-directed RNA polymerase I, II
Q9UTP0	SPAC1250.05|rpl3002	9	16.31	3	7	cyt	cyt	cyt			1	1						1	60S ribosomal protein L30
O74322	SPBC1685.02c|rps1202	10	16.02	2	7	cyt; nuc	cyt; nuc	cyt; nuc		1		1						1	40S ribosomal protein S12 (predict
P40231	SPAC23C11.11|cka1	11	15.77	4	8	cyt; nuc	cyt; nuc	cyt; nuc		1									serine/threonine protein kinase Ck
P17937	SPAC664.06|rpl703	12	15.69	2	3	nuc	cyt; nuc	nuc	1			1						1	60S ribosomal protein L7
P52808	SPAC9G1.03c|rpl3001	13	15.41	5	49	cyt	cyt	cyt			1	1						1	60S ribosomal protein L30
O14062	SPCC962.04|rps1201	14	15.35	5	20	NA	cyt	cyt			1	1						1	40S ribosomal protein S12
P24487	SPAC14C4.14|atp1	15	15.27	10	18	NA	cyt	cyt			1								F1-ATPase alpha subunit
P31209	SPAC57A7.04c|pabp	16	15.17	8	33	cyt	cyt; nuc	cyt			1							1	mRNA export shuttling protein
Q9USZ6	SPBC11G11.03|mrt4	17	15.16	4	9	nuc	nuc	nuc	1										mRNA turnover and ribosome asse
P79013	SPAC31G5.03|rps1101	18	15.11	4	14	cyt; nuc	cyt; nuc	cyt; nuc		1		1						1	40S ribosomal protein S11 (predict
Q9P7H0	SPAC1782.10c|nhp2	19	15.10	2	16	nuc	nuc	nuc	1										RNA-binding protein Nhp2 (predic
P08094	SPBP8B7.06|rpp201	20	15.07	1	4	cyt	cyt	cyt			1	1						1	60S acidic ribosomal protein P2A s
P40370	SPBC1815.01|eno101	21	15.05	5	15	cyt; nuc	cyt; nuc	cyt; nuc		1									enolase
P78761	SPCC613.10|qcr2	22	15.04	13	70	cyt	cyt	cyt			1								ubiquinol-cytochrome-c reductase
O60101	SPBC14F5.04c|pgk1	23	14.99	11	27	cyt; nuc	cyt; nuc	cyt; nuc		1									phosphoglycerate kinase Pgk1 (pre
P56286	SPAC3G9.09c|tif211	24	14.96	1	3	cyt; nuc	cyt; nuc	cyt; nuc		1								1	translation initiation factor eIF2 al
Q9P6K7	SPAC30C2.04	25	14.94	3	7	NA	cyt	cyt			1							1	cofactor for methionyl-and glutam
O13672	SPBC29A3.04|rpl8	26	14.94	10	387	NA	cyt	cyt			1	1						1	60S ribosomal protein L7a/L8 (pre
Q09864	SPAC12G12.04|mcp60	27	14.93	13	113	cyt	cyt	cyt			1								mitochondrial heat shock protein I
Q10192	SPBC11C11.07|rpl1801	28	14.75	2	14	cyt	cyt	cyt			1	1						1	60S ribosomal protein L18
Q9UTJ7	SPAC1556.02c|sdh1	29	14.74	3	4	cyt	cyt	cyt			1								succinate dehydrogenase Sdh1
Q10289	SPAC4A8.11c|fas2	30	14.74	51	449	cyt	cyt	cyt			1								fatty acid synthase alpha subunit L
Q9P546	SPAPJ698.02c|rps002	31	14.72	1	3	cyt	cyt	cyt			1	1						1	40S ribosomal protein S0B

Q09757	SPAC24H6.07|rps901	32	14.71	1	5	cyt	cyt	cyt			1	1						1	40S ribosomal protein S9
P78811	SPCC1322.04	33	14.71	8	16	cyt; nuc	cyt; nuc	cyt; nuc		1									UTP-glucose-1-phosphate uridylylt
O94754	SPCC1259.01c|rps1802	34	14.68	4	19	cyt; nuc	cyt; nuc	cyt; nuc		1		1						1	40S ribosomal protein S18 (predict
P87140	SPAC57A7.10c|sec21	35	14.67	7	15	cyt	cyt	cyt			1								coatomer gamma subunit Sec21 (p
P08093	SPBC839.04|rpl803	36	14.66	2	8	cyt; nuc	cyt; nuc	cyt; nuc		1		1						1	60S ribosomal protein L8 (predicte
P78814	SPAC16.02c|srp2	37	14.66	7	58	NA	nuc	nuc	1										mRNA export factor Srp2
P21911	SPAC140.01|sdh2	38	14.65	6	9	cyt	cyt	cyt			1								succinate dehydrogenase (ubiquin
O14036	SPAC2C4.03c|smd2	39	14.65	2	2	nuc	nuc	nuc	1										Sm snRNP core protein Smd2
O43060	SPBC4C3.07	40	14.63	2	6	cyt; nuc	cyt; nuc	cyt; nuc		1								1	translation initiation factor eIF3f
O42661	SPAC27D7.07c|smd1	41	14.56	2	3	cyt; nuc	cyt; nuc	cyt; nuc		1									Sm snRNP core protein Smd1
O13775	SPAC17A5.15c	42	14.55	6	24	cyt; nuc	cyt; nuc	cyt; nuc		1								1	glutamate-tRNA ligase (predicted)
O14460	SPCP31B10.07|eft202	43	14.48	1	2	cyt	cyt	cyt			1	1						1	translation elongation factor 2 (EF-
Q9UTM3	SPAPB8E5.02c|rpn502	44	14.47	1	2		nuc	nuc	1										19S proteasome regulatory subuni
O14317	SPAC1071.08|rpp203	45	14.44	1	2	cyt	cyt	cyt			1	1						1	60S acidic ribosomal protein P2C s
O59736	SPBC3E7.16c|leu3	46	14.42	3	5	cyt	cyt	cyt			1								2-isopropylmalate synthase (predi
P05933	SPAC3A12.14|cam1	47	14.41	3	7	cyt; nuc	cyt; nuc	cyt; nuc		1									calmodulin Cam1
Q9P7U2	SPAC977.14c	48	14.41	5	12	cyt; nuc	cyt; nuc	cyt; nuc		1									aldo/keto reductase, unknown bio
Q9USX8	SPBC1861.08c|lea1	49	14.36	3	5	cyt; nuc	cyt; nuc	cyt; nuc		1									U2 snRNP-associated protein Lea1
Q09179	SPAC23H4.06|gln1	50	14.34	3	4	cyt; nuc	cyt; nuc	cyt; nuc		1									glutamate-ammonia ligase Gln1
Q10157	SPBC17G9.10|rpl1102	51	14.31	3	10	cyt	cyt	cyt			1	1						1	60S ribosomal protein L11
P30655	SPAC4A8.13c|pts1	52	14.30	3	11	cyt; nuc	cyt; nuc	cyt; nuc		1									20S proteasome component beta
Q09682	SPAC13C5.01c	53	14.29	2	5	cyt; nuc	cyt; nuc	cyt; nuc		1									20S proteasome component alpha
P56329	SPAC32A11.04c|tif212	54	14.29	2	3	cyt	cyt	cyt			1							1	translation initiation factor eIF2 be
O14363	SPBC30D10.18c|rpl102	55	14.24	5	18	cyt	cyt	cyt			1	1						1	60S ribosomal protein L10a
Q9HEQ9	SPBC660.11|tcg1	56	14.16	1	2	cyt	cyt	cyt			1			1					single-stranded telomeric binding
P08092	SPBC19C2.05|ran1	57	14.15	1	4	cyt; nuc	cyt; nuc	cyt; nuc		1									serine/threonine protein kinase Ra
Q10163	SPAC26A3.08|smb1	58	14.09	3	10	cyt; nuc	cyt; nuc	cyt; nuc		1									Sm snRNP core protein Smb1
Q96WV5	SPBPJ4664.04	59	14.08	5	16	cyt	cyt	cyt			1								coatomer alpha subunit (predicted
P36620	SPBP35G2.07|ilv1	60	14.06	2	10	cyt	cyt	cyt			1								acetolactate synthase catalytic sub
O60143	SPBC18H10.12c|rpl701	61	14.06	2	14	nuc	cyt; nuc	nuc	1			1						1	60S ribosomal protein L7
Q9HGL8	SPBC800.04c|rpl4301	62	14.03	1	6	cyt	cyt	cyt			1	1						1	60S ribosomal protein L37a (predic
Q9P3A9	SPAC1565.05	63	14.02	2	6	cyt	cyt	cyt			1								sequence orphan
P40921	SPAC29A4.02c	64	13.96	6	11	cyt	cyt	cyt			1							1	translation elongation factor EF-1 ;
P78937	SPBC27.08c|sua1	65	13.94	8	26	cyt	cyt	cyt			1								sulfate adenylyltransferase
O14293	SPAC9E9.09c	66	13.87	8	20	cyt; nuc	cyt; nuc	cyt; nuc		1									aldehyde dehydrogenase (predicte
O14164	SPAC4A8.16c|tif33	67	13.87	5	9	cyt	cyt	cyt			1							1	translation initiation factor eIF3c
Q09184	SPAC23H4.09|cdb4	68	13.83	6	14	cyt; nuc	cyt; nuc	cyt; nuc		1									curved DNA-binding protein Cdb4
P31411	SPAC637.05c|vma2	69	13.83	5	7	cyt	cyt	cyt			1								V-type ATPase V1 subunit B
O74825	SPBC337.14|rpb4	70	13.77	2	6	cyt; nuc	nuc	cyt; nuc		1					1		1		DNA-directed RNA polymerase II c

Q9UT32	SPAC8F11.04	71	13.74	2	5	nuc	nuc	nuc	1			1						1	U3 snoRNP-associated protein Cic1
O14049	SPAC2C4.16c|rps801	72	13.64	1	1	cyt	cyt	cyt			1	1						1	40S ribosomal protein S8 (predicte
O94438	SPAC22H12.04c|rps102	73	13.63	2	6	cyt	cyt	cyt			1	1						1	40S ribosomal protein S3a (predict
O94681	SPBC776.15c|kgd2	74	13.61	3	8	cyt	cyt	cyt			1								dihydrolipoamide S-succinyltransf
O14388	SPBC685.07c|rpl2701	75	13.56	4	106	cyt; nuc	cyt; nuc	cyt; nuc		1		1						1	60S ribosomal protein L27
O74887	SPCC576.03c|tpx1	76	13.55	3	13	cyt; nuc	cyt; nuc	cyt; nuc		1								1	thioredoxin peroxidase Tpx1
Q9US22	SPAC1783.08c|rpl1502	77	13.54	1	2	nuc	cyt; nuc	nuc	1			1						1	60S ribosomal protein L15b
O74760	SPBC17D11.05|tif32	78	13.51	2	9	cyt	cyt	cyt			1							1	translation initiation factor eIF3a
O59734	SPBC3E7.14|smf1	79	13.48	1	4	cyt; nuc	cyt; nuc	cyt; nuc		1									Sm snRNP core protein Smf1
O14065	SPCC962.01	80	13.45	2	5	cyt		cyt			1								C2 domain protein
O74855	SPCC18.05c	81	13.43	2	2	nuc	nuc	nuc	1										notchless-like protein
O60163	SPBC19F5.04	82	13.43	2	5	cyt	cyt	cyt			1								aspartate kinase (predicted)
P15398	SPBC4C3.05c|nuc1	83	13.33	13	26	cyt	cyt; nuc	cyt			1				1				DNA-directed RNA polymerase I co
P78854	SPAC1A6.04c|plb1	84	13.32	3	20	cyt; nuc	cyt; nuc	cyt; nuc		1									phospholipase B homolog Plb1
O94305	SPCC5E4.05c	85	13.31	2	2	cyt	cyt	cyt			1								mitochondrial serine hydrolase (pr
O74893	SPCC576.09|rps20	86	13.30	2	4	cyt	cyt	cyt			1	1						1	40S ribosomal protein S20
Q9C0X6	SPAPB8E5.09|rvb1	87	13.23	2	2	cyt; nuc	cyt; nuc	cyt; nuc		1				1		1			AAA family ATPase Rvb1
P30821	SPAC3C7.14c|obr1	88	13.23	2	3	cyt; nuc	cyt; nuc	cyt; nuc		1			1		1				ubiquitinated histone-like protein
O42867	SPCC1322.11|rpl2302	89	13.14	1	2	cyt; nuc	cyt; nuc	cyt; nuc		1		1						1	60S ribosomal protein L23
P78812	SPBC660.16	90	13.11	2	4	cyt	cyt	cyt			1								phosphogluconate dehydrogenase
O14008	SPAC29A4.05|cam2	91	13.09	1	2	cyt; nuc	cyt; nuc	cyt; nuc		1									myosin I light chain Cam2
O14459	SPACUNK4.06c|rpb7	92	13.00	1	2	cyt; nuc	cyt; nuc	cyt; nuc		1					1		1		DNA-directed RNA polymerase cor
Q9UTG4	SPAC1805.11c|rps2602	93	12.94	1	1	cyt	cyt	cyt			1	1						1	40S ribosomal protein S26 (predict
O75002	SPCC1840.01c|mog1	94	12.84	1	1	cyt; nuc	cyt; nuc	cyt; nuc		1									Ran GTPase binding protein Mog1
O74891	SPCC576.07|ret3	95	12.82	2	8	cyt; nuc	cyt; nuc	cyt; nuc		1									coatomer zeta subunit (predicted)
Q9UUG1	SPAC926.08c	96	12.81	4	10	cyt; nuc	cyt; nuc	cyt; nuc		1									Brix domain protein Rpf2 (predicte
O42699	SPCC1682.14|rpl1902	97	12.79	1	5	cyt	cyt	cyt			1	1						1	60S ribosomal protein L19
Q9URX2	SPAC922.04	98	12.77	1	9	cyt	cyt	cyt			1								sequence orphan
Q9P6P5	SPAC644.18c|bet3	99	12.76	1	2	cyt; nuc	cyt; nuc	cyt; nuc		1									TRAPP complex subunit Bet3 (prec
O13687	SPAC11E3.07|vma4	100	12.75	2	4	cyt	cyt	cyt			1								V-type ATPase V1 subunit E (predi
Q10353	SPAC22E12.13c|rpl2403	101	12.74	1	2	nuc	cyt; nuc	nuc	1			1						1	60S ribosomal protein L24-3 (L30)
O14206	SPAC1B9.03c	102	12.74	2	5	nuc	nuc	nuc	1										RNA-binding protein involved in ri
Q10193	SPBC11C11.08|srp1	103	12.71	1	2	nuc	nuc	nuc	1										SR family protein Srp1
Q9UQY2	SPAC22F8.06|pam1	104	12.66	3	5	cyt; nuc	cyt; nuc	cyt; nuc		1									20S proteasome component beta
O14241	SPAC6F6.10c|arc2	105	12.66	1	1	cyt; nuc	cyt; nuc	cyt; nuc		1									ARP2/3 actin-organizing complex s
Q9UT48	SPAC821.05	106	12.64	1	1	cyt; nuc	cyt; nuc	cyt; nuc		1								1	translation initiation factor eIF3h (
Q9USN3	SPCC16A11.02|utp13	107	12.59	4	10	NA	nuc	nuc	1										U3 snoRNP-associated protein Utp
O14362	SPBC30D10.17c|smi1	108	12.58	3	5	cyt		cyt			1								cell wall biosynthesis/ cell cycle re
Q09878	SPCC584.01c	109	12.54	1	1	cyt	cyt	cyt			1								sulfite reductase NADPH flavoprot

P78773	SPCC191.02c	110	12.52	1	2	cyt; nuc	cyt; nuc	cyt; nuc		1									acetyl-CoA ligase (predicted)
O74496	SPCC285.08|ret2	111	12.49	2	5	cyt; nuc	cyt; nuc	cyt; nuc		1									coatomer delta subunit Ret2 (prec
O42945	SPBC16H5.10c|prp43	112	12.47	9	17	nuc	nuc	nuc	1										ATP-dependent RNA helicase Prp4
O13849	SPAC19G12.10c|cpy1	113	12.46	1	2	cyt	cyt	cyt			1								vacuolar carboxypeptidase Y
Q9P7C0	SPAC2E1P5.05	114	12.43	3	4	nuc	nuc	nuc	1										U3 snoRNP-associated protein Rrp
O74895	SPCC576.11|rpl15	115	12.42	1	3	nuc	cyt; nuc	nuc	1			1						1	60S ribosomal protein L15
P78804	SPCC622.12c|gdh1	116	12.42	3	5	cyt	cyt; nuc	cyt			1								NADP-specific glutamate dehydrog
O74862	SPCC18.12c	117	12.39	2	8	nuc	nuc	nuc	1										rRNA processing protein (predicte
O42707	SPBC4F6.05c	118	12.38	1	3	cyt	cyt	cyt			1								lectin family glycoprotein receptor
O94517	SPBC646.16	119	12.38	7	18	cyt; nuc	cyt; nuc	cyt; nuc		1									20S proteasome component alpha
P31406	SPAC343.05|vma1	120	12.37	4	6	cyt	cyt	cyt			1								V-type ATPase V1 domain, subunit
P00332	SPCC13B11.01|adh1	121	12.37	8	27	NA	cyt	cyt			1								alcohol dehydrogenase Adh1
O94641	SPBC16D10.08c	122	12.36	6	9	cyt; nuc	cyt; nuc	cyt; nuc		1									heat shock protein Hsp104 (predic
P40232	SPAC1851.03|ckb1	123	12.31	1	3	nuc	nuc	nuc	1						1				CK2 family regulatory subunit Ckb:
P78831	SPCC1235.14|ght5	124	12.28	3	3	cyt		cyt			1								hexose transporter Ght5
Q09181	SPBC776.09|ste13	125	12.27	5	13	cyt		cyt			1								ATP-dependent RNA helicase Ste1
O74341	SPBC1A4.08c|cct3	126	12.26	8	16	cyt; nuc	cyt; nuc	cyt; nuc		1									chaperonin-containing T-complex
O60135	SPBC18H10.02|lcf1	127	12.26	4	13	cyt	cyt	cyt			1								long-chain-fatty-acid-CoA ligase Lc
O94268	SPBP8B7.20c	128	12.25	5	5	nuc	nuc	nuc	1										RNA methyltransferase Nop2 (pre
Q9Y7T8	SPCC63.12c|pup3	129	12.24	3	5	cyt; nuc	cyt; nuc	cyt; nuc		1									20S proteasome component beta
Q10055	SPAC1F5.10	130	12.23	1	1	cyt; nuc	cyt; nuc	cyt; nuc		1									ATP-dependent RNA helicase, eIF4
P07669	SPCC24B10.21|tpi1	131	12.17	4	6	cyt; nuc	cyt; nuc	cyt; nuc		1									triosephosphate isomerase
Q9URM2	SPBC2G5.05	132	12.17	4	6	cyt; nuc	cyt; nuc	cyt; nuc		1									transketolase (predicted)
P24782	SPBP8B7.16c|dbp2	133	12.16	4	4	cyt; nuc	cyt; nuc	cyt; nuc		1									ATP-dependent RNA helicase Dbp:
P35551	SPBC2D10.10c|fib1	134	12.15	5	14	cyt; nuc	nuc	cyt; nuc		1									fibrillarin
Q9P7W3	SPBC1703.07	135	12.10	5	22	cyt; nuc	cyt; nuc	cyt; nuc		1									ATP citrate synthase subunit 1 (pre
O14150	SPBC18H10.13|rps1402	136	12.10	1	7	nuc	cyt; nuc	nuc	1			1						1	40S ribosomal protein S14
O60128	SPBC16G5.14c|rps3	137	12.10	6	41	cyt	cyt	cyt			1	1						1	40S ribosomal protein S3
Q9Y7R8	SPCC306.08c	138	12.08	7	22	cyt	cyt	cyt			1								malate dehydrogenase
Q9P6N8	SPAC823.08c	139	12.07	2	3	cyt; nuc	cyt; nuc	cyt; nuc		1									ATP-dependent RNA helicase Rrp3
P42656	SPAC8E11.02c|rad24	140	12.06	4	8	cyt; nuc	cyt	cyt; nuc		1			1		1		1		14-3-3 protein Rad24
O74976	SPCC1827.03c	141	12.05	7	15	cyt	cyt	cyt			1								acetyl-CoA ligase (predicted)
Q9P6R6	SPBC13E7.04|atp16	142	12.05	2	2	cyt	cyt	cyt			1								F1-ATPase delta subunit (predicte
Q9USW5	SPBC21B10.10|rps402	143	12.03	1	1	cyt; nuc	cyt; nuc	cyt; nuc		1		1						1	40S ribosomal protein S4 (predicte
Q9USP0	SPCC11E10.07c	144	12.03	1	3	cyt; nuc	cyt; nuc	cyt; nuc		1								1	translation initiation factor eIF2B a
O74922	SPCC757.12	145	12.02	1	1	cyt	cyt	cyt			1								alpha-amylase homolog (predicte
O13907	SPAC22A12.16	146	12.02	5	8	cyt; nuc	cyt; nuc	cyt; nuc		1									ATP-citrate synthase subunit 2 (pr
O94620	SPBC1289.11|spf38	147	12.01	1	2	nuc	nuc	nuc	1										splicing factor Spf38
Q9HE11	SPAC20H4.01|utp5	148	12.01	6	15	cyt; nuc	cyt; nuc	cyt; nuc		1									U3 snoRNP-associated protein Utp

Q09154	SPBC16H5.06|rip1	149	12.00	2	5	cyt	cyt	cyt			1							ubiquinol-cytochrome-c reductase
P05732	SPAC3A12.10|rpl2001	150	11.97	5	25	cyt	cyt	cyt			1	1					1	60S ribosomal protein L20a
Q9UT95	SPAC323.04	151	11.96	1	3	cyt	cyt	cyt			1							mitochondrial ATPase (predicted)
O42938	SPBC16H5.02|pfk1	152	11.96	8	26	cyt	cyt	cyt			1							6-phosphofructokinase (predicted
Q9UUN5	SPCC306.02c	153	11.96	1	2	ND	cyt	cyt			1							Rab GTPase binding (predicted)
Q10208	SPAC4H3.10c|pyk1	154	11.96	5	19	cyt	cyt	cyt			1							pyruvate kinase (predicted)
O14180	SPAC4F8.04	155	11.95	1	3	nuc	nuc	nuc	1									Brix domain protein Rpf1 (predicte
P50999	SPBC106.06|cct4	156	11.93	4	11	cyt; nuc	cyt; nuc	cyt; nuc		1								chaperonin-containing T-complex
Q9P7B4	SPAC521.03	157	11.93	3	6	cyt	cyt	cyt			1							short chain dehydrogenase (predi
Q09171	SPBC30D10.13c|pdb1	158	11.92	1	3	cyt	cyt	cyt			1							pyruvate dehydrogenase e1 comp
O74407	SPCC1223.07c|drs1	159	11.91	2	4	cyt	cyt	cyt			1						1	cytoplasmic aspartate-tRNA ligase
Q10318	SPAC17G8.06c	160	11.91	2	3	cyt	cyt	cyt			1							dihydroxy-acid dehydratase (predi
P36579	SPBC4F6.18c|arf1	161	11.91	3	7	cyt	cyt	cyt			1							ADP-ribosylation factor, Arf family
O14027	SPAC29B12.04|snz1	162	11.90	2	4	cyt	cyt	cyt			1							pyridoxine biosynthesis protein
Q9P775	SPBC1711.16	163	11.90	1	2	cyt; nuc	cyt; nuc	cyt; nuc		1								WD repeat protein (predicted)
O74873	SPCC31H12.07|sec231	164	11.89	1	1	NA	cyt	cyt			1							COPII cargo receptor subunit Sec2
Q10161	SPAC26A3.05|chc1	165	11.89	25	123	cyt	cyt	cyt			1							clathrin heavy chain Chc1 (predict
Q9P7X8	SPBP23A10.07|rpa2	166	11.89	7	10	cyt	nuc	cyt			1				1			DNA-directed RNA polymerase I co
O14053	SPCC1672.07	167	11.89	3	7	cyt; nuc	cyt; nuc	cyt; nuc		1								U3 snoRNP-associated protein Utp
Q92352	SPAC6G9.07c|arc4	168	11.88	1	1	cyt; nuc	cyt; nuc	cyt; nuc		1								ARP2/3 actin-organizing complex s
O59866	SPCC338.15|wbp1	169	11.88	3	5	cyt	cyt	cyt			1							dolichyl-di-phosphooligosaccharid
O42937	SPBC16C6.13c|sec27	170	11.87	7	14	cyt; nuc	cyt; nuc	cyt; nuc		1								coatomer beta' subunit (predicted
Q09916	SPAC1F7.02c|has1	171	11.87	5	10	nuc	nuc	nuc	1									ATP-dependent RNA helicase Has1
P10989	SPBC32H8.12c|act1	172	11.87	4	9	cyt	cyt; nuc	cyt			1			1		1		actin Act1
Q10349	SPAC1F12.07	173	11.86	4	7	cyt	cyt	cyt			1							phosphoserine aminotransferase (
O74966	SPBC4B4.05|smg1	174	11.86	1	3	cyt; nuc	cyt; nuc	cyt; nuc		1								Sm snRNP core protein Smg1
O74548	SPCC777.09c|arg1	175	11.86	1	1	cyt; nuc	cyt; nuc	cyt; nuc		1								acetylornithine aminotransferase
Q9UT97	SPAC323.02c|pup2	176	11.85	1	4	cyt; nuc	cyt; nuc	cyt; nuc		1								20S proteasome component alpha
O43079	SPBC947.02|apl2	177	11.85	1	1	ND	cyt	cyt			1							AP-1 adaptor complex subunit bet
Q9P7X7	SPBP23A10.08|alp5	178	11.85	1	2	NA	nuc	nuc	1				1	1	1	1		actin-like protein Arp4
P87147	SPBC25H2.05|egd2	179	11.83	1	2	cyt	cyt	cyt			1	1						nascent polypeptide-associated co
Q9HGL6	SPBC800.06|brx1	180	11.83	6	13	cyt; nuc	cyt; nuc	cyt; nuc		1								ribosome biogenesis protein Brx1
O74752	SPBC1734.11	181	11.81	5	16	cyt; nuc	cyt; nuc	cyt; nuc		1								DNAJ domain protein Mas5 (predi
Q9US39	SPAC1039.02	182	11.81	3	5	cyt	cyt	cyt			1							phosphoprotein phosphatase (pre
P40375	SPCC794.12c|mae2	183	11.81	2	2	cyt	cyt	cyt			1							malic enzyme, malate dehydrogen
O94365	SPBC428.19c	184	11.81	6	17	cyt; nuc	cyt; nuc	cyt; nuc		1								U3 snoRNP protein Utp15 (predict
Q9P784	SPBC1711.06|rpl401	185	11.80	1	1	cyt	cyt	cyt			1	1					1	60S ribosomal protein L2
Q9UUI5	SPAC22F8.08|sec24	186	11.80	3	4	cyt	cyt	cyt			1							COPII cargo receptor Sec24/Sfb2 s
P28189	SPAC6F6.07c|rps13	187	11.79	4	17	cyt	cyt	cyt			1	1					1	40S ribosomal protein S13 (predict

O60198	SPBC14F5.05c|sam1	188	11.79	2	7	cyt; nuc	cyt; nuc	cyt; nuc		1							S-adenosylmethionine synthetase
Q9UTH5	SPAC1786.02	189	11.78	6	17	cyt	cyt	cyt			1						phospholipase (predicted)
P56287	SPAC8C9.15c|tif225	190	11.77	1	1	cyt	cyt	cyt			1					1	translation initiation factor eIF2B e
O14344	SPBC2F12.14c|gua1	191	11.77	4	18	cyt	cyt	cyt			1						IMP dehydrogenase Gua1 (predict
O13823	SPAC19A8.07c	192	11.76	2	7	nuc	nuc	nuc	1								U3 snoRNP-associated protein Imp
Q9P7X1	SPBP23A10.15c|qcr1	193	11.76	4	8	cyt	cyt	cyt			1						mitochondrial processing peptidas
Q9C1X1	SPBC713.04c	194	11.76	4	11	cyt; nuc	cyt; nuc	cyt; nuc		1							U3 snoRNP-associated protein Utp
Q06975	SPBC20F10.01|gar1	195	11.75	2	7	nuc	nuc	nuc	1								snoRNP pseudouridylase complex
P41887	SPAC926.04c|hsp90	196	11.75	7	33	cyt	cyt	cyt			1		1		1		Hsp90 chaperone
P41390	SPAC4D7.08c|ade4	197	11.72	8	13	cyt		cyt			1						amidophosphoribosyltransferase A
O74400	SPBC4F6.14	198	11.72	3	3	nuc	nuc	nuc	1								RNA-binding protein
O74766	SPBC24C6.04	199	11.71	1	1	cyt	cyt	cyt			1						delta-1-pyrroline-5-carboxylate de
P78965	SPBC17A3.07|pgr1	200	11.69	3	3	cyt	cyt; nuc	cyt			1						mitochondrial glutathione reducta
Q9UTT2	SPAPJ698.03c|prp12	201	11.69	7	12	ND	nuc	nuc	1								U2 snRNP-associated protein Sap1
P19117	SPAC23C11.05	202	11.69	1	1	cyt; nuc	cyt; nuc	cyt; nuc		1							inorganic pyrophosphatase (predic
O94514	SPBC646.10c	203	11.68	4	6	nuc	nuc	nuc	1								U3 snoRNP protein Nop56 (predict
O94444	SPAC637.10c|rpn10	204	11.65	2	6	cyt; nuc	cyt; nuc	cyt; nuc		1							19S proteasome regulatory subuni
O59816	SPCC794.07|lat1	205	11.65	4	31	cyt	cyt	cyt			1						dihydrolipoamide S-acetyltransfera
Q9P7R0	SPAC1834.02|aro1	206	11.65	21	39	cyt	cyt	cyt			1						pentafunctional aromatic polypept
Q9Y7R7	SPCC306.07c	207	11.63	1	1	nuc	nuc	nuc	1								U3 snoRNP-associated protein Cic1
Q9URY0	SPAC890.04c	208	11.63	5	12	nuc	nuc	nuc	1								ribosome biogenesis protein Ytm1
Q9UTM4	SPAC1420.02c|cct5	209	11.63	2	8	cyt	cyt	cyt			1						chaperonin-containing T-complex
Q09694	SPAC227.18|lys3	210	11.63	3	13	cyt		cyt			1						saccharopine dehydrogenase Lys3
O00087	SPAC1002.09c|dld1	211	11.62	11	24	cyt	cyt	cyt			1						dihydrolipoamide dehydrogenase
O14250	SPAC6G10.04c	212	11.62	4	5	cyt; nuc	cyt; nuc	cyt; nuc		1							20S proteasome component alpha
Q09826	SPBC646.13|sds23	213	11.61	3	3	cyt; nuc	cyt; nuc	cyt; nuc		1							inducer of sexual development Sds
P09627	SPAC1071.10c|pma1	214	11.61	12	137	cyt	cyt	cyt			1						P-type proton ATPase, P3-type Pm
P87243	SPCC4G3.18	215	11.60	1	1	nuc	nuc	nuc	1								ribosome biogenesis protein Rix1 (
Q10335	SPBC582.07c|rpn7	216	11.60	2	3	cyt; nuc	cyt; nuc	cyt; nuc		1				1			19S proteasome regulatory subuni
O74340	SPBC1A4.07c	217	11.60	5	8	cyt; nuc	nuc	cyt; nuc		1							U3 snoRNP-associated protein Sof
Q9P7D4	SPBP4H10.15	218	11.60	7	18	cyt	cyt	cyt			1	1				1	aconitate hydratase/mitochondria
O59826	SPCC965.06	219	11.59	1	1	cyt; nuc	cyt; nuc	cyt; nuc		1							potassium channel subunit (predic
P36623	SPAC26F1.06|gpm1	220	11.59	2	3	cyt; nuc	cyt; nuc	cyt; nuc		1							monomeric 2,3-bisphosphoglycera
O42931	SPBC16C6.07c|rpt1	221	11.58	1	2	cyt; nuc	cyt; nuc	cyt; nuc		1							19S proteasome regulatory subuni
P22194	SPAC4A8.12c|sds22	222	11.57	1	2	cyt; nuc	cyt; nuc	cyt; nuc		1				1			protein phosphatase regulatory su
O94273	SPBP8B7.25|cyp4	223	11.57	1	2	cyt	cyt	cyt			1						cyclophilin family peptidyl-prolyl c
O13639	SPBC8D2.18c	224	11.56	1	4	cyt	cyt	cyt			1						adenosylhomocysteinase (predicte
O74943	SPCC553.06|swp1	225	11.56	1	1	cyt	cyt	cyt			1						oligosaccharyltransferase delta sul
Q9P7S7	SPAC23G3.06	226	11.55	6	28	nuc	nuc	nuc	1								U3 snoRNP protein Nop58 (predict

Q9P7T6	SPAC694.04c	227	11.54	2	2	cyt; nuc	cyt; nuc	cyt; nuc	1									conserved eukaryotic protein
P35679	SPBP8B7.03c|rpl402	228	11.53	2	2	cyt	cyt	cyt		1	1						1	60S ribosomal protein L2
O60085	SPBC14C8.03|fma2	229	11.53	1	2	cyt	cyt	cyt		1							1	methionine aminopeptidase Fma2
P79071	SPCC622.18|rpl6	230	11.53	4	15	cyt; nuc	cyt; nuc	cyt; nuc	1		1						1	60S ribosomal protein L6
Q10232	SPAC4G9.16c|rpl901	231	11.53	1	3	cyt; nuc	cyt; nuc	cyt; nuc	1		1						1	60S ribosomal protein L9
Q9P3A7	SPAC1565.08|cdc48	232	11.51	16	51	cyt; nuc	cyt; nuc	cyt; nuc	1									AAA family ATPase Cdc48
O94489	SPCC417.08|tef3	233	11.50	3	4	cyt	cyt	cyt		1	1						1	translation elongation factor eEF3
O59770	SPCC1795.04c|pre10	234	11.50	3	7	cyt; nuc	cyt; nuc	cyt; nuc	1									20S proteasome component alpha
P36604	SPAC22A12.15c|bip1	235	11.48	10	40	cyt	cyt; nuc	cyt		1								ER heat shock protein BiP
Q9UUG0	SPAC926.09c|fas1	236	11.48	52	444	cyt	cyt	cyt		1								fatty acid synthase beta subunit Fa
O59852	SPCC191.11|inv1	237	11.48	1	2	NA	cyt	cyt		1								beta-fructofuranosidase
Q9UT05	SPAP8A3.12c|tpp2	238	11.44	10	34	ND	cyt	cyt		1								tripeptidyl-peptidase II Tpp2
Q9UT19	SPAC9.09|met26	239	11.43	3	6	cyt; nuc	cyt; nuc	cyt; nuc	1									homocysteine methyltransferase M
O94709	SPCC1259.09c|pdx1	240	11.41	1	2	cyt	cyt	cyt		1								pyruvate dehydrogenase protein x
Q10147	SPAC1D4.04|cct2	241	11.38	3	8	cyt; nuc	cyt; nuc	cyt; nuc	1									chaperonin-containing T-complex
Q02061	SPAC23G3.01|rpb2	242	11.38	11	23	cyt; nuc	cyt; nuc	cyt; nuc	1			1		1		1		RNA polymerase II complex subun
Q10329	SPBC106.16|pre6	243	11.38	4	9	cyt; nuc	cyt; nuc	cyt; nuc	1									20S proteasome component alpha
O60160	SPBC19C7.12c|omh1	244	11.38	1	1	cyt	cyt	cyt		1								alpha-1,2-mannosyltransferase Or
O94385	SPBC29A10.07	245	11.34	7	8	cyt; nuc	nuc	cyt; nuc	1									nucleoporin Pom152
Q9UTB4	SPAC25B8.04c	246	11.33	2	3	cyt	cyt	cyt		1							1	mitochondrial splicing suppressor
Q09820	SPAC16C9.06c|upf1	247	11.33	6	8	cyt	cyt	cyt		1							1	ATP-dependent RNA helicase Upf1
P05734	SPBC56F2.02|rpl1901	248	11.31	1	20	cyt	cyt	cyt		1	1						1	60S ribosomal protein L19
O74960	SPCC736.15	249	11.30	2	3	cyt		cyt		1								protein kinase inhibitor associated
Q10425	SPAC25G10.08	250	11.28	5	8	cyt	cyt	cyt		1							1	translation initiation factor eIF3b (
P78921	SPBC337.05c|cct8	251	11.28	8	16	cyt	cyt	cyt		1								chaperonin-containing T-complex
O94692	SPBC83.08|rvb2	252	11.28	3	5	cyt; nuc	cyt; nuc	cyt; nuc	1				1		1			AAA family ATPase Rvb2
Q9USP8	SPBC902.05c|idh2	253	11.28	1	1	cyt	cyt	cyt		1								isocitrate dehydrogenase (NAD+) s
Q09740	SPBC12C2.11	254	11.26	2	3	cyt	cyt	cyt		1								glutamine-fructose-6-phosphate tr
P36591	SPCC1223.08c|dfr1	255	11.26	1	1	cyt	cyt	cyt		1								dihydrofolate reductase Dfr1
P22774	SPAC664.11|ssc1	256	11.26	4	9	cyt	cyt	cyt		1								mitochondrial heat shock protein H
O74892	SPCC576.08c|rps2	257	11.25	4	14	cyt	cyt	cyt		1	1						1	40S ribosomal protein S2
O94501	SPBC12D12.03|cct1	258	11.25	7	10	cyt; nuc	cyt; nuc	cyt; nuc	1									chaperonin-containing T-complex
Q11004	SPAC1B3.03c|wis2	259	11.23	1	1	cyt; nuc	cyt; nuc	cyt; nuc	1									cyclophilin family peptidyl-prolyl c
Q92370	SPBC1539.09c|trp1	260	11.22	1	9	cyt	cyt	cyt		1								anthranilate synthase component
O42700	SPCC1020.06c|tal1	261	11.21	1	1	cyt; nuc	cyt; nuc	cyt; nuc	1									transaldolase (predicted)
Q10284	SPBC1709.05|sks2	262	11.20	4	7	cyt	cyt	cyt		1							1	heat shock protein, ribosome asso
O13848	SPAC19G12.09	263	11.19	1	1	cyt; nuc	cyt; nuc	cyt; nuc	1									NADH/NADPH dependent indole-3
O94316	SPBC215.12|cwf10	264	11.19	9	12	cyt; nuc	cyt; nuc	cyt; nuc	1									GTPase Cwf10 (predicted)
P78820	SPAC56E4.04c|cut6	265	11.17	4	12	cyt	cyt	cyt		1								acetyl-CoA/biotin carboxylase

O94579	SPCC1442.06|pre8	266	11.15	2	6	cyt; nuc	cyt; nuc	cyt; nuc		1									20S proteasome component alpha
P53694	SPBC31A8.01c|rtn1	267	11.14	1	1	NA	cyt	cyt			1								reticulon-like protein Rtn1
Q9USQ9	SPBC577.10	268	11.13	1	1	cyt; nuc	cyt; nuc	cyt; nuc		1									20S proteasome component beta
Q9C106	SPAPB1E7.03|rpc82	269	11.11	2	4	nuc	nuc	nuc	1						1				DNA-directed RNA polymerase III c
Q9P7N9	SPBC1718.05|trs31	270	11.10	3	3	cyt; nuc	cyt; nuc	cyt; nuc		1									TRAPP complex subunit Trs31 (pre
Q9UUF7	SPBC146.14c|sec26	271	11.10	4	4	cyt	cyt	cyt			1								coatomer beta subunit (predicted)
O14104	SPAC31G5.04	272	11.10	4	5	cyt	cyt	cyt			1								homoisocitrate dehydrogenase
Q9Y7Y3	SPBC365.04c	273	11.08	2	5	nuc	nuc	nuc	1										RNA-binding protein, involved in ri
P04551	SPBC11B10.09|cdc2	274	11.08	2	2	cyt	cyt	cyt			1								cyclin-dependent protein kinase C
P17478	SPBC23G7.15c|rpp202	275	11.07	1	2	cyt	cyt	cyt			1	1						1	60S acidic ribosomal protein P2B s
P39964	SPAC644.12|cdc5	276	11.06	1	1	nuc	nuc	nuc	1										cell division control protein, splicir
Q10411	SPAC1F3.06c|spo15	277	11.04	3	4	nuc	cyt	nuc	1										sporulation protein Spo15
O59838	SPAC110.04c|pss1	278	11.03	7	11	cyt; nuc	cyt; nuc	cyt; nuc		1									heat shock protein Pss1
Q9USK6	SPCC4B3.17|cbp3	279	11.03	3	6	NA	cyt	cyt			1								ubiquinol cytochrome-c reductase
Q9UU76	SPCP1E11.11|puf6	280	11.01	2	3	nuc	nuc	nuc	1										Puf family RNA-binding protein Pu
Q09794	SPAC22G7.06c|ura1	281	11.01	14	24	cyt	cyt	cyt			1								carbamoyl-phosphate synthase (gl
O94698	SPBC83.15	282	11.00	5	8	nuc	nuc	nuc	1										ribosome biogenesis protein Nsa1
P78810	SPCC1322.14c|vtc4	283	11.00	4	5	cyt	cyt	cyt			1								vacuolar transporter chaperone (V
O59678	SPBC29A3.16|rrs1	284	10.99	2	3	nuc	nuc	nuc	1										ribosome biogenesis protein Rrs1
Q9UUE1	SPBC17G9.11c|pyr1	285	10.99	7	14	cyt	cyt	cyt			1								pyruvate carboxylase Pyr1
Q9Y6Z9	SPAC8E11.10	286	10.97	3	5	cyt	cyt	cyt			1								sorbose reductase (predicted)
O94462	SPAC1687.22c|puf3	287	10.97	1	2	cyt	cyt	cyt			1								RNA-binding protein Puf3 (predict
O13940	SPAC23H3.09c|gly1	288	10.95	2	2	cyt; nuc	cyt; nuc	cyt; nuc		1									threonine aldolase (predicted)
Q9UU78	SPCP1E11.09c|rpp103	289	10.94	2	4	cyt; nuc	cyt; nuc	cyt; nuc		1		1						1	60S acidic ribosomal protein Rpp1
O13692	SPAC11E3.13c|gas5	290	10.93	4	8	cyt		cyt			1								cell wall protein Gas5, 1,3-beta-glu
P36594	SPBC28F2.12|rpb1	291	10.92	7	17	cyt; nuc	cyt; nuc	cyt; nuc		1					1		1		RNA polymerase II large subunit R
Q9USL1	SPCC18B5.10c	292	10.91	2	2	NA	nuc	nuc	1										TREX complex subunit Tex1 (predi
O14081	SPACUNK4.16c	293	10.85	2	3	cyt	cyt	cyt			1								alpha,alpha-trehalose-phosphate s
O13326	SPBC428.11	294	10.81	1	1	cyt; nuc	cyt; nuc	cyt; nuc		1									homocysteine synthase Met17
P36580	SPBC19C2.07|fba1	295	10.77	1	1	cyt; nuc	cyt; nuc	cyt; nuc		1									fructose-bisphosphate aldolase Fb
O42644	SPAC10F6.03c|cts1	296	10.76	1	1	cyt	cyt	cyt			1								CTP synthase Cts1 (predicted)
O60161	SPBC19F5.02c	297	10.75	2	3	cyt; nuc	nuc	cyt; nuc		1									U3 snoRNP protein Utp4 (predicte
P40374	SPBC21H7.07c|his5	298	10.73	1	1	cyt; nuc	cyt; nuc	cyt; nuc		1									imidazoleglycerol-phosphate dehy
Q9UQX0	SPAC1486.01	299	10.71	1	1	cyt	cyt	cyt			1								manganese superoxide dismutase
P22068	SPAC222.12c|atp2	300	10.71	9	52	cyt	cyt	cyt			1								F1-ATPase beta subunit Atp2
P87048	SPBP19A11.03c|mts4	301	10.70	9	19	cyt; nuc	cyt; nuc	cyt; nuc		1									19S proteasome regulatory subuni
P78827	SPBC56F2.12|ilv5	302	10.68	7	40	cyt	cyt	cyt			1								acetohydroxyacid reductoisomera
P87153	SPBC25H2.12c|cct7	303	10.66	6	9	cyt; nuc	cyt; nuc	cyt; nuc		1									chaperonin-containing T-complex
O74928	SPCPB16A4.03c|ade10	304	10.65	3	3	cyt	cyt	cyt			1								IMPcyclohydrolase/phosphoribosy

O94616	SPBC1289.07c|rpc40	305	10.64	3	6	cyt; nuc	cyt; nuc	cyt; nuc		1					1				DNA-directed RNA polymerase I ar
O74733	SPBC1709.08|cft1	306	10.61	3	3	cyt; nuc	cyt; nuc	cyt; nuc		1									cleavage factor one Cft1 (predicte
Q8WZJ7	SPBC839.16	307	10.60	7	9	cyt	cyt	cyt			1								C1-5,6,7,8-tetrahydrofolate (THF) s
O94745	SPBC18E5.12c|mas2	308	10.56	1	1	cyt	cyt	cyt			1								mitochondrial processing peptidas
O42849	SPAC23A1.12c|frs1	309	10.53	1	1	cyt; nuc	cyt; nuc	cyt; nuc		1								1	phenylalanine-tRNA ligase beta su
O94676	SPBC776.08c	310	10.53	5	6	nuc	nuc	nuc	1										Nrap
O13831	SPAC19A8.15|trp2	311	10.53	1	1	cyt; nuc	cyt; nuc	cyt; nuc		1									tryptophan synthase (predicted)
P78946	SPBC29B5.03c|rpl26	312	10.52	1	3	cyt; nuc	cyt; nuc	cyt; nuc		1		1						1	60S ribosomal protein L26 (predict
Q9HFE5	SPBP16F5.07|apm1	313	10.52	1	1	cyt; nuc	cyt; nuc	cyt; nuc		1									AP-1 adaptor complex mu subunit
Q9Y7Z8	SPBC146.13c|myo1	314	10.52	9	14	cyt	cyt	cyt			1								myosin type I
O43036	SPBC3B9.07c|rpa43	315	10.51	1	1	cyt; nuc	cyt; nuc	cyt; nuc		1					1				DNA-directed RNA polymerase I cc
P41838	SPBC16A3.05c|rae1	316	10.49	2	2	nuc	nuc	nuc	1										RNA export factor, nucleoporin Ra
O13370	SPCC1795.11|sum3	317	10.49	1	3	cyt	cyt	cyt			1							1	ATP-dependent RNA helicase Sum
O13696	SPAC11G7.03|idh1	318	10.49	1	2	cyt	cyt	cyt			1								isocitrate dehydrogenase (NAD+) s
O59704	SPBC36.07|iki3	319	10.47	1	1	cyt	cyt	cyt			1								elongator subunit Iki3 (predicted)
O43063	SPBC4C3.10c|pre3	320	10.46	5	11	cyt; nuc	cyt; nuc	cyt; nuc		1									20S proteasome component beta
Q9P4X3	SPAC959.03c	321	10.45	3	5	nuc	nuc	nuc	1										U3 snoRNP-associated protein Utp
Q9UT24	SPAC9.03c|brr2	322	10.41	1	1	cyt; nuc	cyt; nuc	cyt; nuc		1									U5 snRNP complex subunit Brr2 (p
Q09785	SPAC13G6.06c|gcv2	323	10.41	2	2	cyt	cyt	cyt			1								glycine cleavage complex subunit I
Q10233	SPAC4G9.08c|rpc2	324	10.36	2	2	cyt	cyt; nuc	cyt			1				1				DNA-directed RNA polymerase III c
Q9Y7U7	SPCC645.08c|snd1	325	10.33	1	2	cyt	cyt	cyt			1								RNA-binding protein Snd1
Q10490	SPAC26F1.13c|lrs1	326	10.32	1	1	cyt	cyt	cyt			1							1	cytoplasmic leucine-tRNA ligase Lr
Q09177	SPAC1687.01|rpc19	327	10.31	1	3	NA	nuc	nuc	1						1				DNA-directed RNA polymerase I ar
Q9USZ1	SPBC1306.01c	328	10.28	1	1	cyt	cyt	cyt			1							1	mitochondrial translation elongati
O60179	SPBC23E6.04c|utp10	329	10.21	7	10	nuc	nuc	nuc	1										U3 snoRNP-associated protein Utp
O13641	SPBC8D2.16c	330	10.17	2	2	cyt; nuc	cyt; nuc	cyt; nuc		1									DUF171 family protein
Q9HGL4	SPBC800.08|gcd10	331	10.16	1	1	nuc	cyt; nuc	nuc	1									1	tRNA (m1A) methyltransferase, tra
Q8TFH1	SPAPB17E12.13|rpl1802	332	10.14	2	38	cyt	cyt	cyt			1	1						1	60S ribosomal protein L18
Q9UUE5	SPBC17G9.04c|nup85	333	10.13	1	1	cyt; nuc	cyt; nuc	cyt; nuc		1									nucleoporin Nup85
Q9UU81	SPCP1E11.06|apl4	334	10.11	1	1	cyt	cyt	cyt			1								AP-1 adaptor complex gamma sub
Q10101	SPAC18G6.14c|rps7	335	10.09	2	7	cyt; nuc	cyt; nuc	cyt; nuc		1		1						1	40S ribosomal protein S7 (predicte
P31318	SPAC4G9.09c|arg11	336	10.06	2	2	cyt	cyt	cyt			1								N-acetyl-gamma-glutamyl-phosph
P78963	SPBC16H5.11c|skb1	337	10.01	1	1	cyt	cyt; nuc	cyt			1								type II protein arginine N-methyltr
O14254	SPAC6G10.08|idp1	338	10.00	1	1	cyt	cyt	cyt			1								isocitrate dehydrogenase Idp1
O14156	SPAC4A8.03c|ptc4	339	9.87	1	1	cyt	cyt	cyt			1								protein phosphatase 2C Ptc4
Q09781	SPAC13G6.02c|rps101	340	9.86	1	2	cyt	cyt	cyt			1	1						1	40S ribosomal protein S3a
P37382	SPCC1442.10c|rpb3	341	9.84	1	1	cyt; nuc	cyt; nuc	cyt; nuc		1					1		1		RNA polymerase II subunit 3
P40235	SPBC3H7.15|hhp1	342	9.81	2	2	cyt; nuc	cyt; nuc	cyt; nuc		1									serine/threonine protein kinase Hl
Q9USI6	SPCC645.05c|myo2	343	9.78	2	2	nuc	cyt; nuc	nuc	1										myosin II heavy chain

O13793	SPAC17G6.15c	344	9.75	1	2	cyt	cyt	cyt			1								MTC tricarboxylate transporter
Q10480	SPAC17C9.08|pnu1	345	9.71	2	2	NA	cyt	cyt			1								mitochondrial endodeoxyribonucl
O74484	SPCC1906.01|mpg1	346	9.60	1	1	cyt	cyt	cyt			1								mannose-1-phosphate guanyltran
O13811	SPAC17H9.14c	347	9.52	1	1	NA	cyt	cyt			1								protein disulfide isomerase
O13282	SPCC5E4.03c|taf5	348	9.46	1	1	cyt; nuc	cyt; nuc	cyt; nuc		1			1	1	1	1	1		SAGA complex subunit/TATA-bind
O94284	SPBC2G5.06c|hmt2	349	9.46	1	1	cyt	cyt	cyt			1								sulfide-quinone oxidoreductase
P04913	SPCC622.09|htb1	350	9.44	2	3	nuc	nuc	nuc	1					1		1			histone H2B Htb1
Q9US13	SPAC607.05|rpn9	351	9.35	1	1	NA	nuc	nuc	1										19S proteasome regulatory subuni
Q09100	SPAC3G6.04|rnp24	352	9.31	1	1	nuc	nuc	nuc	1										RNA-binding protein Rnp24
O13981	SPAC25H1.07	353	9.25	3	5	cyt	cyt	cyt			1								ER membrane protein complex sul
Q1K9C2	SPAC10F6.01c|sir1	354	9.23	1	2	cyt	cyt	cyt			1								sulfite reductase Sir1
O60164	SPBC19F5.05c|ppp1	355	9.04	1	2	nuc	nuc	nuc	1										pescadillo-family BRCT domain pro
Q9USN5	SPCC132.04c|gdh2	356	9.01	1	1	cyt	cyt	cyt			1								NAD-dependent glutamate dehydı
Q09130	SPBC17G9.09|tif213	357	8.97	4	5	cyt	cyt	cyt			1							1	translation initiation factor eIF2 ga
Q9USU5	SPBC29A10.08|gas2	358	8.96	2	3	cyt	cyt	cyt			1								1,3-beta-glucanosyltransferase Ga
O14275	SPAC8C9.06c	359	8.96	1	1	cyt	cyt	cyt			1							1	mitochondrial translation regulato
O13637	SPBC8D2.20c|sec31	360	8.94	1	1	cyt	cyt	cyt			1								COPII-coated vesicle component S
O42932	SPBC16C6.08c|qcr6	361	8.91	1	2	cyt	cyt	cyt			1								ubiquinol-cytochrome-c reductase
O14121	SPAC3A11.07	362	8.89	1	1	cyt	cyt	cyt			1								NADH dehydrogenase
O94666	SPBC651.08c|rpc1	363	8.79	1	1	cyt	nuc	cyt			1				1				DNA-directed RNA polymerase III (
O13878	SPAC1B3.13	364	8.70	2	4	nuc	nuc	nuc	1										U3 snoRNP-associated protein Nar
Q9C102	SPAPB1E7.07|glt1	365	8.68	2	3	cyt	cyt	cyt			1								glutamate synthase Glt1 (predicte
O94515	SPBC646.11|cct6	366	8.67	1	1	cyt; nuc	cyt; nuc	cyt; nuc		1									chaperonin-containing T-complex
O13753	SPAC17A2.03c|vma6	367	8.65	1	1	nuc	cyt; nuc	nuc	1										V-type ATPase V0 subunit d (predi
O94401	SPCC126.08c	368	8.56	1	1	cyt	cyt	cyt			1								lectin family glycoprotien receptor
Q10176	SPAC27F1.07|ost1	369	8.48	4	15	cyt	cyt	cyt			1								dolichyl-diphospho-oligosaccharid
P41878	SPAC31G5.13|rpn11	370	8.40	1	1	cyt; nuc	cyt; nuc	cyt; nuc		1									19S proteasome regulatory subuni
P09322	SPBC8D2.03c|hhf2	371	8.38	5	164	nuc	nuc	nuc	1					1		1			histone H4 h4.2
P87177	SPBC3D6.12	372	8.28	6	13	cyt; nuc	cyt; nuc	cyt; nuc		1									U3 snoRNA associted protein Dip2
O59855	SPCC1739.13|ssa2	373	8.07	5	13	cyt; nuc	cyt; nuc	cyt; nuc		1									heat shock protein Ssa2
P87051	SPAC57A10.03|cyp1	374	7.82	1	1	cyt; nuc	cyt; nuc	cyt; nuc		1									cyclophilin family peptidyl-prolyl c
O14124	SPAC3A11.10c	375	7.74	1	1	cyt	cyt	cyt			1								dipeptidyl peptidase (predicted)
Q9UUC6	SPBC19C2.14|smd3	376	7.73	3	5	nuc	nuc	nuc	1										Sm snRNP core protein Smd3

Note: a, rank is based on the abundance; b, abundance is based on the median of the unique peptide abundances; c, number of unique peptides idnetified; d, number of spectra identified; e, subcellular localization based on the study by Matsuyama et al (2006); f, localization based on the gene ontology (CC); g, localization based on Matsuyama and GO terms when Matsuyama's data are not available; h, nuclear localization; i, both nuclear and cytoplasmic localization; j, cytoplasmic localization; k, riobsomal proteins; l, regulation of transcription; m, chromosome organization; n, transcription; o, chromatin organization; p, type II transcription; p, translaction; r, gene product based on the S. pombe genome annotation (www.genedb.org).

Supplemental Table S4. List of growth fitness scores of the Bioneer deletion strains

Order	Gene	Loc[a]	Ratio (log2)[b]	GFS[c]
1	SPAC1002.18|urg3	cyt; nuc	-2.93	0.79
2	SPAC1071.02	cyt; nuc	-1.90	1.98
3	SPAC1142.06|get3	cyt; nuc	-0.79	0.93
4	SPAC11E3.01c|swr1	nuc	-2.29	0.40
5	SPAC1250.05|rpl32	cyt	3.23	1.10
6	SPAC13A11.06	cyt; nuc	4.34	1.50
7	SPAC13G6.02c|rps11	cyt	-2.12	0.71
8	SPAC13G6.06c|gcv2	cyt	-4.53	0.74
9	SPAC13G6.07c|rps61	cyt	-3.19	0.76
10	SPAC13G7.02c|ssa1	cyt	2.16	0.93
11	SPAC144.03|ade2	cyt; nuc	-2.58	0.67
12	SPAC1486.01	cyt	-1.46	3.69
13	SPAC14C4.14|atp1	cyt	-5.50	0.90
14	SPAC1556.02c|sdh1	cyt	-1.72	0.83
15	SPAC1556.04c|cdd1	cyt; nuc	-6.79	1.02
16	SPAC1556.07|pmm1	cyt; nuc	-3.63	0.86
17	SPAC15A10.07	cyt; nuc	-0.67	0.86
18	SPAC1687.21	cyt; nuc	-4.87	0.64
19	SPAC17G6.15c	cyt	3.76	1.10
20	SPAC17H9.14c	cyt	-3.90	0.69
21	SPAC1805.05|cki3	cyt	2.04	1.14
22	SPAC18G6.04c|shm2	cyt	-2.01	1.14
23	SPAC18G6.14c|rps7	cyt; nuc	-4.41	0.71
24	SPAC19G12.09	cyt; nuc	-0.59	1.24
25	SPAC1A6.04c|plb1	cyt; nuc	0.73	1.24
26	SPAC1B1.03c|kap95	nuc	-1.30	0.79
27	SPAC1B3.03c|wis2	cyt; nuc	2.25	0.90
28	SPAC1F12.02c	cyt; nuc	-3.66	1.26
29	SPAC1F3.09|mug161	nuc	3.54	0.76
30	SPAC1F7.08|fio1	cyt	-3.98	0.79
31	SPAC20G8.09c	nuc	-5.30	0.62
32	SPAC227.18|lys3	cyt	-2.14	1.17
33	SPAC22A12.11|dak1	cyt	-1.08	0.90
34	SPAC22A12.16	cyt; nuc	1.90	1.31
35	SPAC22H12.04c|rps12	cyt	-2.86	0.74
36	SPAC23A1.17	cyt	5.52	1.24
37	SPAC23H4.09|cdb4	cyt; nuc	1.68	0.67
38	SPAC24H6.11c	cyt	2.51	1.26
39	SPAC25G10.05c|his1	cyt	-1.65	0.40
40	SPAC26H5.02c	nuc	-5.44	1.57
41	SPAC27D7.06	cyt	-3.81	0.71
42	SPAC27F1.06c	nuc	3.26	1.40
43	SPAC29A4.02c	cyt	3.62	0.95
44	SPAC2F7.03c|pom1	cyt	-4.57	2.93
45	SPAC2F7.09c	cyt	-3.17	1.21
46	SPAC30C2.04	cyt	1.46	1.21

47	SPAC31A2.16|gef2	cyt; nuc	-2.50	1.12
48	SPAC31G5.03|rps111	cyt; nuc	-5.27	0.98
49	SPAC31G5.04	cyt	-2.72	1.31
50	SPAC31G5.14|gcv1	cyt	-3.91	1.19
51	SPAC328.03|tps1	cyt	-1.50	0.81
52	SPAC32A11.02c	cyt	-5.35	0.86
53	SPAC3A11.07	cyt	-5.31	0.43
54	SPAC3A12.10|rpl21	cyt	1.17	0.50
55	SPAC3C7.07c	cyt; nuc	0.91	0.93
56	SPAC3C7.14c|obr1	cyt; nuc	-1.59	1.29
57	SPAC3F10.11c|abc2	cyt	4.08	1.24
58	SPAC3H5.07|rpl72	cyt; nuc	-1.93	0.67
59	SPAC3H8.10|spo2	cyt	-0.99	1.24
60	SPAC4A8.04|isp6	cyt	-2.28	1.33
61	SPAC4F8.15|itr1	cyt	4.51	0.43
62	SPAC4G9.16c|rpl91	cyt; nuc	-3.06	0.43
63	SPAC513.07	cyt; nuc	-0.82	1.33
64	SPAC57A7.04c|pabp	cyt	1.04	0.71
65	SPAC5H10.06c|adh4	cyt	-4.44	0.93
66	SPAC607.06c	cyt; nuc	-4.20	1.29
67	SPAC631.01c|acp2	cyt; nuc	-2.83	1.21
68	SPAC6C3.04|cit1	cyt	-2.61	0.86
69	SPAC6C3.08	cyt; nuc	-0.69	1.17
70	SPAC6G10.08|idp1	cyt	-1.12	1.02
71	SPAC806.07|ndk1	cyt; nuc	-1.64	0.86
72	SPAC821.05	cyt; nuc	-0.60	0.24
73	SPAC8E11.03c|dmc1	cyt; nuc	-3.20	1.26
74	SPAC8E11.10	cyt	-0.65	0.55
75	SPAC8F11.03|msh3	nuc	5.86	0.67
76	SPAC926.09c|fas1	cyt	-0.97	1.29
77	SPAC959.07|rps43	cyt	1.76	0.05
78	SPAC977.14c	cyt; nuc	1.61	1.55
79	SPAC9G1.05	cyt; nuc	1.58	1.38
80	SPAC9G1.10c	cyt	-2.29	0.81
81	SPACUNK4.10	cyt; nuc	-4.28	1.14
82	SPACUNK4.16c	cyt	1.02	0.88
83	SPAP8A3.12c|tpp2	cyt	4.51	1.31
84	SPAPB24D3.08c	cyt; nuc	1.07	1.29
85	SPBC1105.02c|lys4	cyt	-1.37	0.57
86	SPBC1198.08	cyt; nuc	-2.40	0.93
87	SPBC11G11.03|mrt4	nuc	2.10	2.69
88	SPBC1289.11|spf38	nuc	1.33	1.67
89	SPBC12C2.07c	cyt; nuc	-1.73	1.07
90	SPBC12C2.08|dnm1	cyt	-2.31	1.19
91	SPBC12C2.12c|glo1	cyt; nuc	-2.44	1.21
92	SPBC1347.02|fkbp39	nuc	2.24	1.02
93	SPBC13G1.02	cyt; nuc	-2.88	1.19
94	SPBC146.13c|myo1	cyt	3.32	1.48
95	SPBC14F5.03c|kap123	cyt; nuc	-2.23	0.05

96	SPBC15D4.15|pho2	cyt; nuc	-2.86	1.21
97	SPBC1685.02c|rps122	cyt; nuc	2.93	1.05
98	SPBC16A3.02c	cyt	-1.69	1.07
99	SPBC16C6.02c|vps132	cyt	-2.25	0.81
100	SPBC16C6.08c|qcr6	cyt	1.28	1.14
101	SPBC16D10.08c	cyt; nuc	1.57	0.43
102	SPBC16G5.05c	cyt	1.57	0.79
103	SPBC16G5.11c|bag11	cyt; nuc	-1.72	0.88
104	SPBC16G5.14c|rps3	cyt	-1.45	1.26
105	SPBC1709.05|sks2	cyt	-3.21	0.05
106	SPBC1711.08	cyt	1.71	0.83
107	SPBC1711.13|his2	cyt; nuc	-2.06	0.74
108	SPBC17D1.07c	nuc	4.91	1.24
109	SPBC17F3.01c|rga5	cyt	-3.43	1.24
110	SPBC17G9.09|tif213	cyt	2.12	0.88
111	SPBC17G9.10|rpl112	cyt	-5.14	1.00
112	SPBC1921.05|ape2	cyt	0.64	2.38
113	SPBC19C7.02|ubr1	nuc	3.95	1.45
114	SPBC19C7.09c|uve1	cyt; nuc	4.83	0.67
115	SPBC1A4.02c|leu1	cyt	-2.56	0.95
116	SPBC1E8.03c	cyt	-5.81	1.00
117	SPBC215.08c|arg4	cyt	-2.55	0.88
118	SPBC215.11c	cyt; nuc	0.75	1.05
119	SPBC216.05|rad3	cyt	1.42	0.83
120	SPBC21C3.08c|car2	cyt; nuc	-1.44	1.36
121	SPBC21C3.19	cyt; nuc	-0.83	0.83
122	SPBC21H7.07c|his5	cyt; nuc	-1.11	1.02
123	SPBC25H2.05|egd2	cyt	-1.17	0.88
124	SPBC27.08c|sua1	cyt	-1.61	0.83
125	SPBC2D10.10c|fib1	cyt; nuc	-1.55	1.14
126	SPBC2D10.17|clr1	nuc	-6.13	0.88
127	SPBC2G5.06c|hmt2	cyt	-1.41	1.02
128	SPBC30B4.08|eri1	cyt	6.54	0.90
129	SPBC30D10.05c	cyt; nuc	-2.58	1.55
130	SPBC30D10.13c|pdb1	cyt	-3.12	1.21
131	SPBC30D10.18c|rpl12	cyt	2.26	0.71
132	SPBC32F12.03c|gpx1	cyt; nuc	-2.78	1.00
133	SPBC336.10c|tif512	cyt; nuc	-2.19	0.52
134	SPBC337.16|cho1	cyt	-1.83	1.05
135	SPBC365.01	cyt; nuc	-1.31	1.60
136	SPBC3B8.03	cyt	-1.68	1.40
137	SPBC3E7.02c|hsp16	cyt; nuc	4.12	0.95
138	SPBC3E7.16c|leu3	cyt	-2.27	1.02
139	SPBC3F6.01c	nuc	-3.05	0.38
140	SPBC409.10|ade7	cyt; nuc	-2.75	0.81
141	SPBC428.02c|eca39	cyt; nuc	-2.55	1.45
142	SPBC428.05c|arg12	cyt	-1.60	1.05
143	SPBC428.07|meu6	cyt; nuc	-4.92	1.19
144	SPBC428.11	cyt; nuc	-2.70	1.07

145	SPBC530.05	cyt; nuc	3.90	2.12
146	SPBC56F2.04|utp2	nuc	-5.75	1.02
147	SPBC609.05|pob3	cyt; nuc	0.77	0.21
148	SPBC646.13|sds23	cyt; nuc	1.44	1.31
149	SPBC713.03	cyt	-2.50	1.10
150	SPBC725.15|ura5	cyt; nuc	-1.87	0.93
151	SPBC776.09|ste13	cyt	1.74	1.33
152	SPBC800.05c|atb2	cyt; nuc	-0.70	4.50
153	SPBC83.02c|rpl432	cyt	3.15	0.93
154	SPBC8D2.03c|hhf2	nuc	1.86	2.24
155	SPBC8D2.18c	cyt	-4.40	0.98
156	SPBC947.08c	nuc	4.90	1.93
157	SPBP35G2.07|ilv1	cyt	1.41	1.29
158	SPBP4H10.12	cyt; nuc	-2.29	0.48
159	SPBP4H10.13|rps232	nuc	-3.81	0.71
160	SPBP8B7.06|rpp21	cyt	1.13	0.62
161	SPBP8B7.11	cyt	3.91	1.26
162	SPBP8B7.25|cyp4	cyt	-1.06	0.79
163	SPBPJ4664.06|gpt1	cyt	4.54	0.45
164	SPCC1183.02	cyt	-2.46	1.21
165	SPCC1223.02|nmt1	cyt; nuc	2.94	0.10
166	SPCC1259.01c|rps182	cyt; nuc	-4.15	1.12
167	SPCC1281.07c	cyt; nuc	1.32	1.05
168	SPCC132.01c	cyt	1.80	1.02
169	SPCC1322.11|rpl233	cyt; nuc	-2.37	1.02
170	SPCC1450.12	cyt; nuc	2.00	1.02
171	SPCC1494.03|arz1	cyt; nuc	-0.76	1.79
172	SPCC16C4.10	cyt; nuc	-3.43	1.02
173	SPCC1827.03c	cyt	-1.79	0.88
174	SPCC1840.02c|bgs4	cyt	3.76	0.74
175	SPCC1840.09	cyt	-2.74	1.19
176	SPCC18B5.05c	cyt; nuc	-3.55	1.79
177	SPCC191.02c	cyt; nuc	-0.63	0.90
178	SPCC24B10.18	nuc	-4.77	1.12
179	SPCC31H12.04c|rpl122	cyt	1.06	0.76
180	SPCC330.06c	cyt; nuc	-3.17	1.40
181	SPCC338.14	cyt; nuc	-2.76	0.88
182	SPCC364.06|nap1	cyt	1.77	1.14
183	SPCC584.15c	cyt	-4.01	0.71
184	SPCC645.08c|snd1	cyt	0.81	0.74
185	SPCC794.07|lat1	cyt	2.65	0.90
186	SPCC794.09c|ef1a-a	cyt	-2.44	0.98
187	SPCC962.04|rps121	cyt	3.52	0.29
188	SPCPB16A4.03c|ade1	cyt	-2.88	1.12

Note: a, subcellular localization; b, ratio between normal and SILAC labeled proteins; c, growth fitness score
